# Supplementary material for: Exosomal mitochondrial tRNAs and miRNAs as potential predictors of inflammation in renal proximal tubular epithelial cells
Source: Mol Ther Nucleic Acids. 2022 May 4;28:794–813. doi: 10.1016/j.omtn.2022.04.035 (PMC9136061; doi:10.1016/j.omtn.2022.04.035)
Supplement: Document S1. Figures S1–S11 and Tables S1–S7 [file mmc1.pdf]

OMTN, Volume *28*

Supplemental information

Exosomal mitochondrial tRNAs and miRNAs as potential predictors of inflammation in renal proximal tubular epithelial cells

Glory Ranches, Maximilian Zeidler, Roman Kessler, Martina Hoelzl, Michael W. Hess, Jonathan Vosper, Paul Perco, Herbert Schramek, Kai K. Kummer, Michaela Kress, Anne Krogsdam, Michael Rudnicki, Gert Mayer, and Alexander Huettenhofer

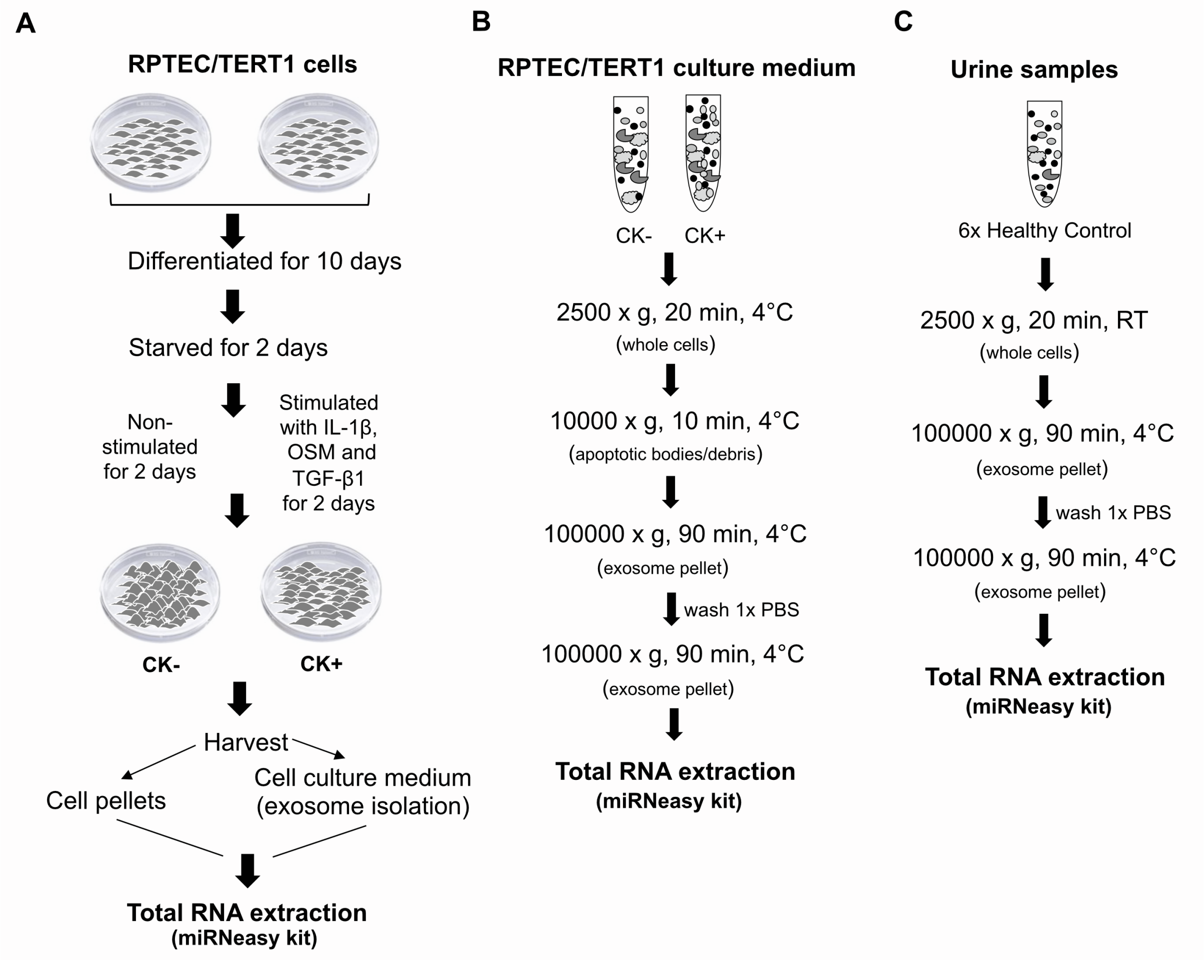

Figure S1. Schematic representation of RPTEC cytokine stimulation and exosome isolation methods employed in RPTEC culture medium and urine samples. A) An equal number of RPTECs were grown as describe in ‘Materials and Methods’. CK- indicates non-stimulated cells and CK+ represents cytokine-stimulated cells or diseased-cells. **B)** An equal volume of RPTEC culture medium from each sample (A) were processed for ultracentrifugation as indicated and the resulting exosome pellets were analysed for total RNA extraction. **C)** An equal volume of urine samples from healthy control group (n=6) were processed for ultracentrifugation as indicated and the urinary exosome pellets were analysed similar to (B).

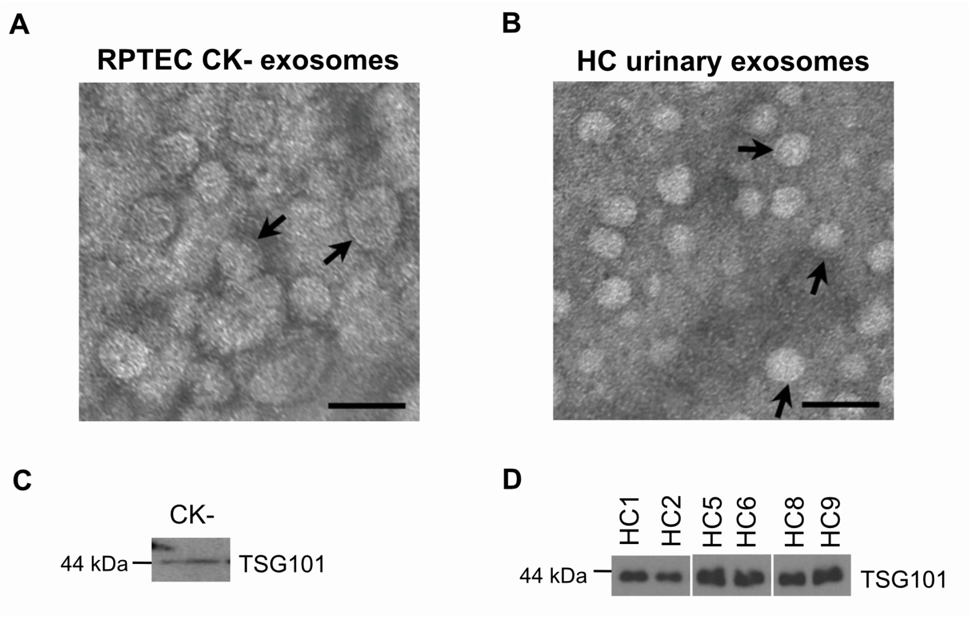

Figure S2. Characterisation of exosomes derived from non-stimulated RPTECs and healthy control urine samples. A) Transmission electron microscopy (TEM) of exosomes derived from non-stimulated RPTECs (CK-) and **B)** urinary exosomes from healthy control (HC) group; arrows point to the exosome membrane bilayer; scale bar= 50nm. **C)** Immunoblot analysis of TSG101 in exosomal lysates from CK- and **D)** urinary exosome samples obtained from HC group (n=6).

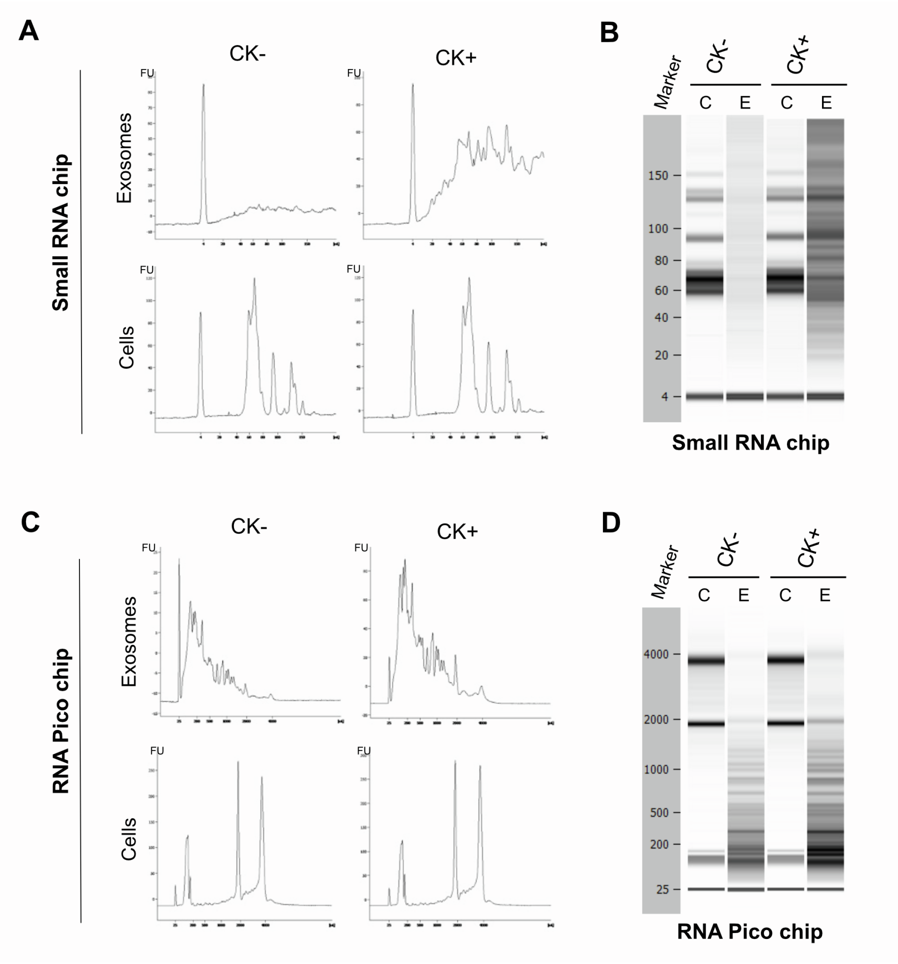

Figure S3. Bioanalyzer profile of total RNA extract from RPTEC exosomes or cells. A) Electropherogram profile of total exosomal RNA extracts (upper panels) and total cellular RNA extracts (lower panels) from either non-stimulated (CK-) or cytokine-stimulated cells (CK+) samples using small RNA chip (<200 nt). An equal amount of total cellular RNA (5 ng) and an equal volume of total exosomal RNA extract (1 µl) for each sample was used for loading. Total exosomal RNA extracts of CK- were undiluted (1:0) while CK+ was diluted in water (1:6). **B)** PAGE gel profile indicating the distribution and molecular size of total exosomal (E) and cellular (C) RNA populations of (A). **C)** Electropherogram profile of total exosomal RNA extracts (upper panels) and total cellular RNA extracts (lower panels) as in (A) using RNA pico chip. **D)** PAGE gel analysis showing the distribution and molecular size of exosomal (E) and cellular (C) RNA populations in (C).

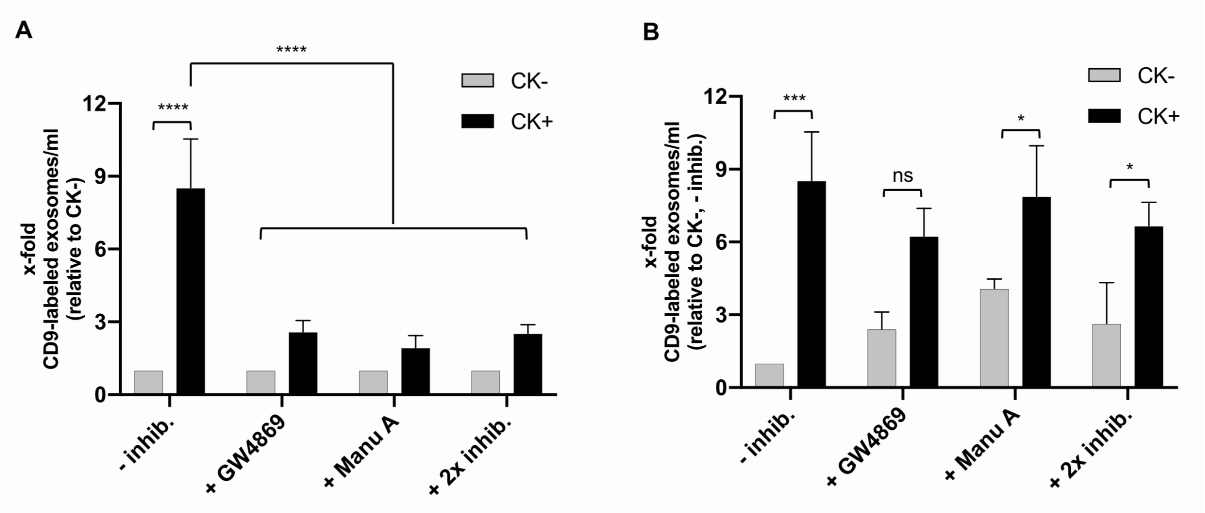

Figure S4. The effect of GW4869 and/or manumycin A on exosome release in RPTECs. A) The fold change (x-fold) of exosome release in cytokine-stimulated sample (CK+) relative to non-stimulated (CK-) sample in the absence or presence of a single inhibitor, (i.e. GW4869 or manumycin A (Manu A)) or two inhibitors (2x inhib.) was analysed by fluorescence-based detection of anti-CD9 (Alexa-647 nm)-labeled exosomes using microplate reader (see Materials and Methods). Bar graph represents mean ± SD of three independent experiments. Data were analysed using two-way ANOVA, with multiple comparisons and Bonferroni correction (GraphPad Prism, 8.0.1). **** indicates $p<0.0001$. **B)** Analysis of anti-CD9 (Alexa-647 nm)-labeled exosomes derived from CK- or CK+ RPTECs under similar conditions as in (A). The fold change (x-fold) of CD9-positive exosomes for each sample was calculated relative to CK- sample without inhibitor (-inhib.). Data were analysed using two-way ANOVA, with multiple comparisons and Bonferroni correction (GraphPad Prism, 8.0.1). * indicates $p<0.05$, *** indicates $p<0.001$ and ns represents not significant.

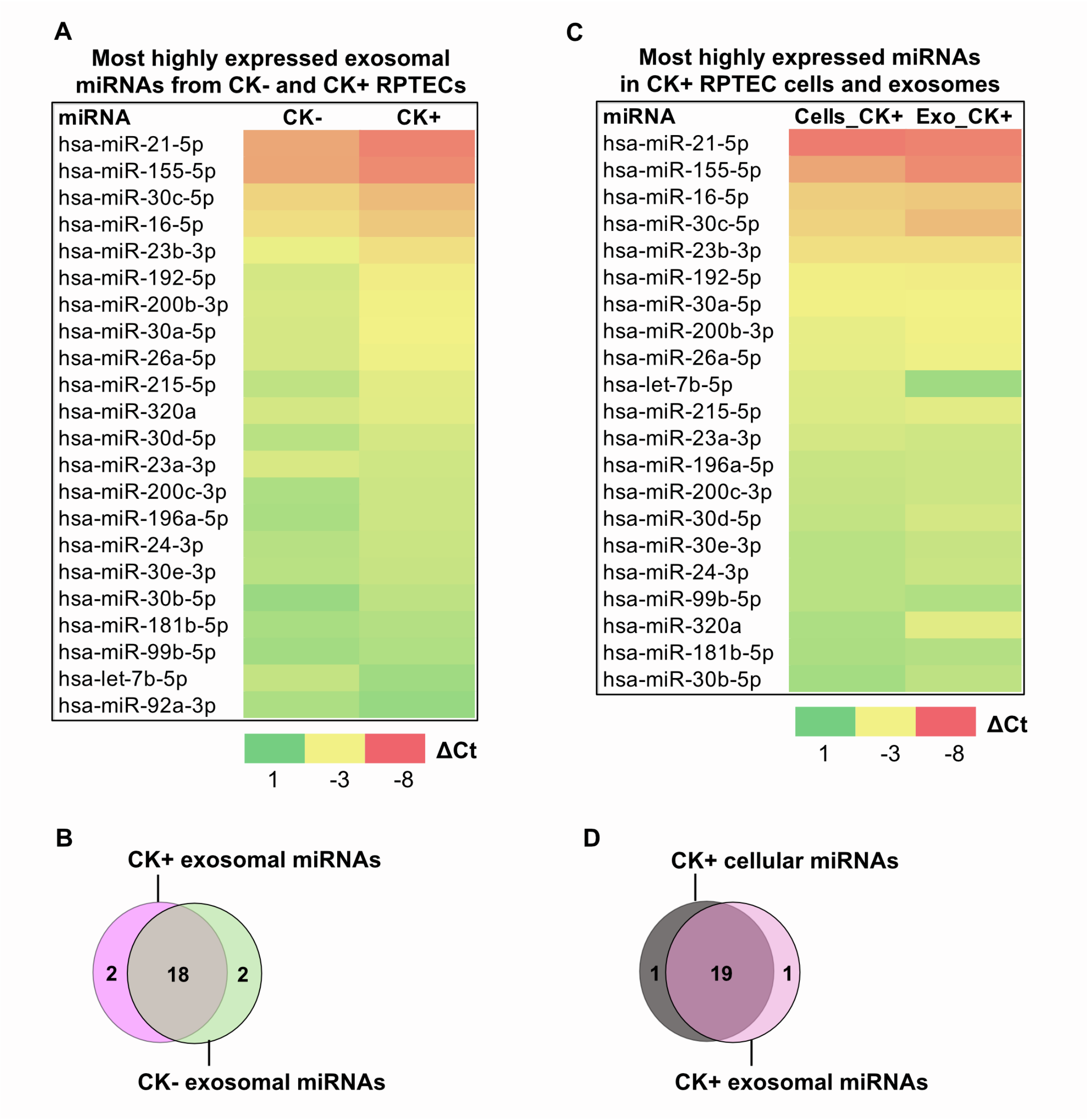

Figure S5. Most abundant exosomal miRNAs in RPTEC cells and exosomes. A) Heat map of 20 most highly abundant RPTEC exosomal miRNAs derived from non-stimulated (CK-) and cytokine-stimulated (CK+). Heat map colors indicate the normalized ΔCt ($Ct_{target\ miRNA}$ -$Ct_{reference\ 6x\ miRNAs}$) values of each target miRNA. Red indicates lower ΔCt (higher expression) and green indicates higher ΔCt (lower expression). **B)** Venn diagram of (A) showing the overlap of most highly abundant exosomal miRNAs between CK- and CK+. **C)** Most highly abundant miRNAs derived from CK+ RPTEC cells and exosomes. Similar analysis was performed as in (A). **D)** Overlap of (C) showing that the majority of most highly abundant miRNAs from CK+ RPTECs are secreted and released via exosomes.

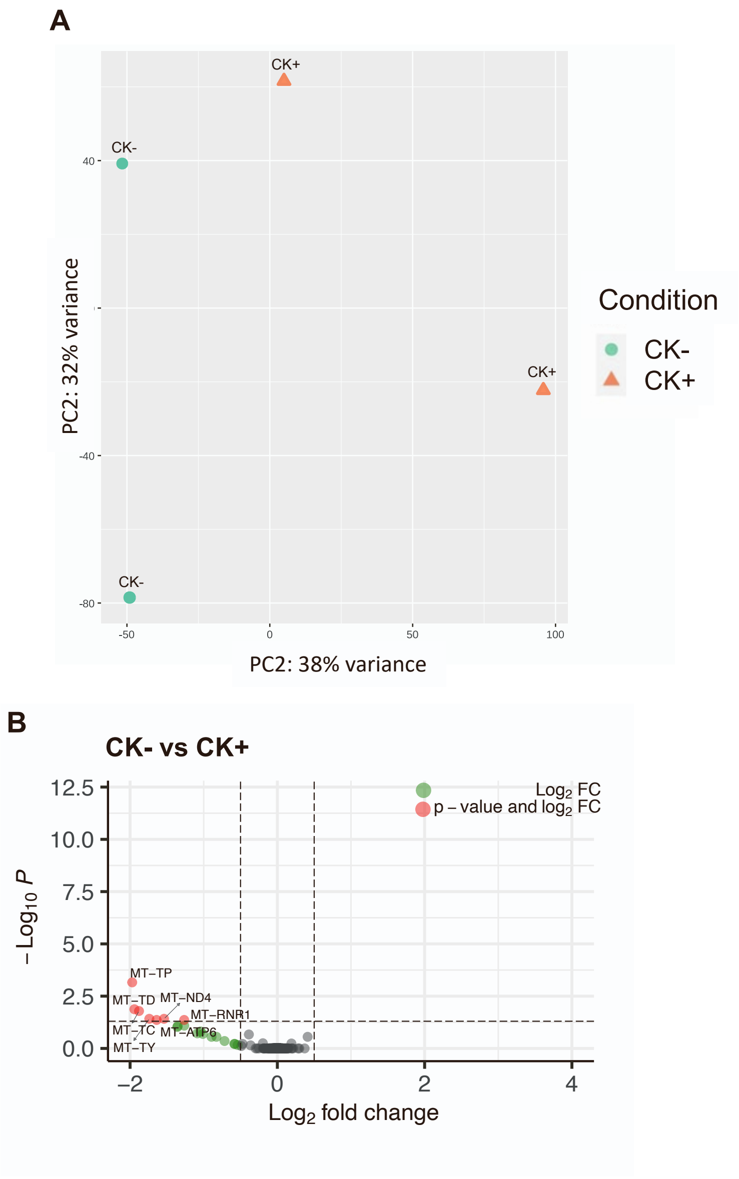

Figure S6. Analysis of differentially abundant exosomal RNAs. A) Principal component analysis (PCA) of exosomal RNA expression from cytokine-stimulated (CK+) and non-stimulated (CK-) RPTECs. **B)** Volcano plot analysis of differentially expressed exosomal mt-RNAs (adjusted $p<0.05$) derived from CK+ (red) and that from CK- RPTECs (green).

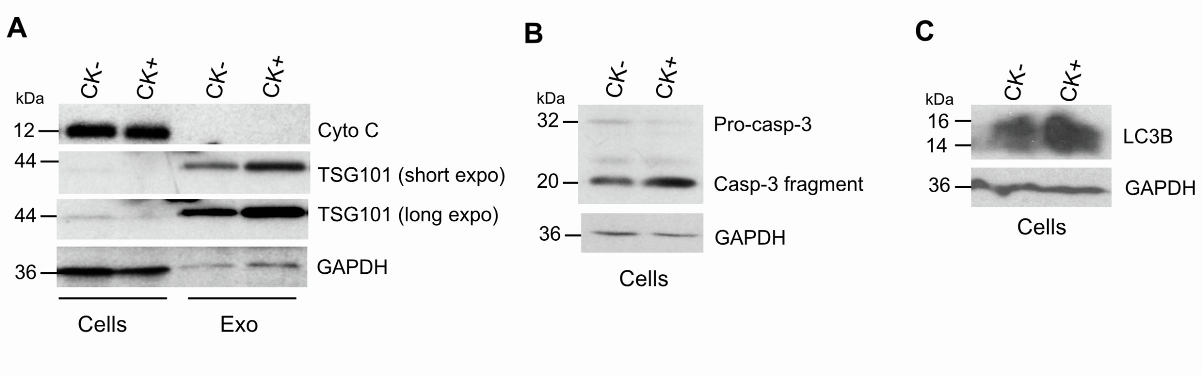

Figure S7. Immunoblot analysis of TSG10, cytochrome C, caspase-3 and LC3B.

RPTECs were either non-stimulated (CK-) or cytokine-stimulated (CK+). Total cell lysates (Cells) and exosomal lysates (Exo) under CK- or CK+ condition were analysed by SDS-PAGE and the protein expressions of **A)** exosome marker tumor suppressor gene (TSG101) and mitochondrial protein cytochrome C (Cyto C); **B)** pro-caspase-3 full length (Pro-casp-3) and caspase-3 fragment (Casp-3); and **C)** LC3B were detected by immunoblotting. GAPDH was used as a loading control. Western blot images (**A-C**) are representative of at least two independent experiments.

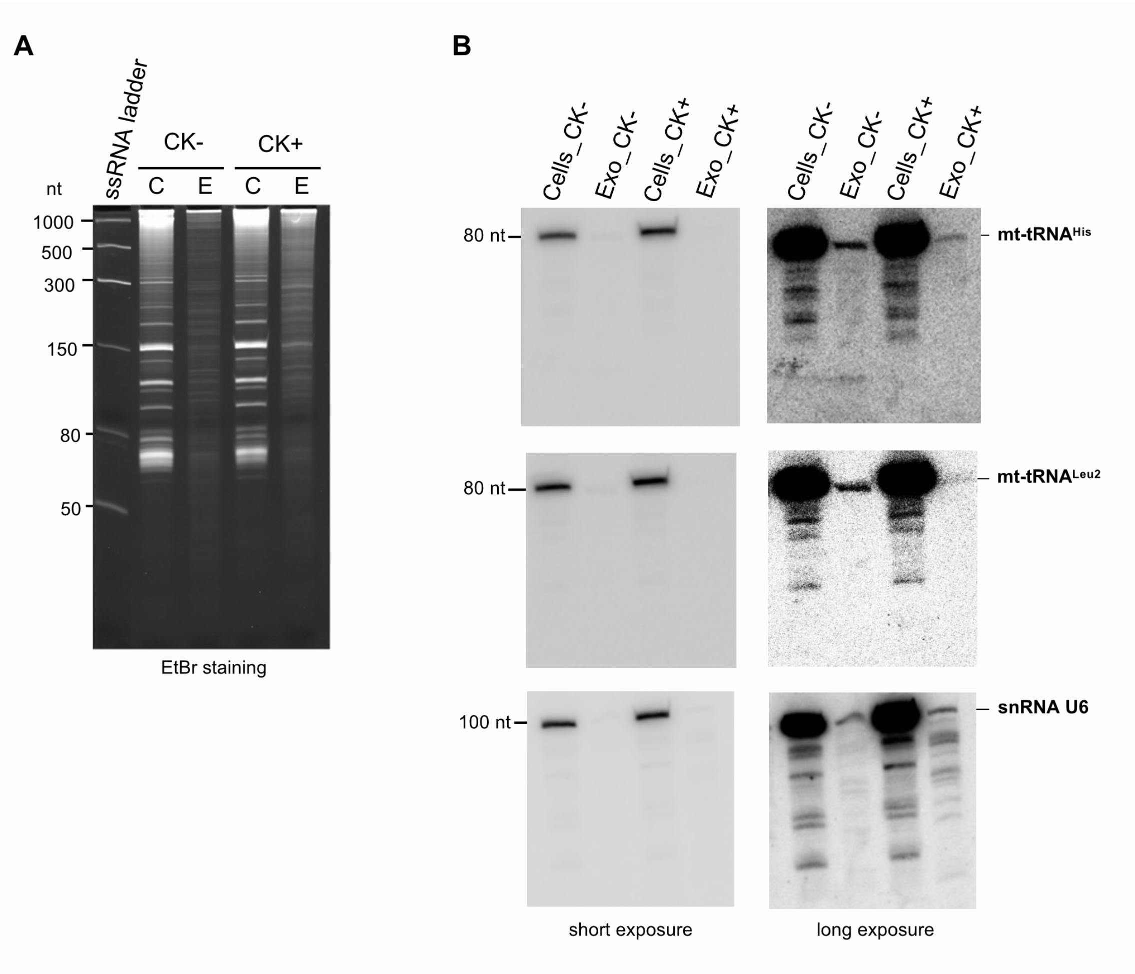

Figure S8. A) Ethidium bromide (EtBr) staining of total cellular RNA (C) and total exosomal RNA (E) derived either from non-stimulated (CK-) or cytokine-stimulated (CK+) RPTECs (see Materials and Methods). **B)** Northern blot analysis of mt-tRNA^{His}, mt-tRNA^{Leu2} and snRNA U6 (uncut membranes).

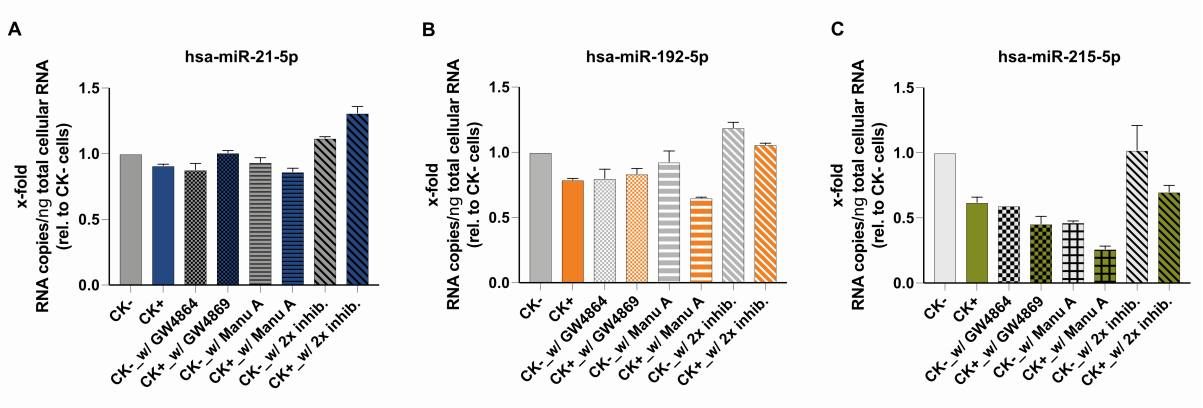

Figure S9. Comparison of cellular miRNAs, hsa-miR-21-5p, -215-5p and -192-5p expressions in RPTECs under different conditions. A-C) The expression of three candidate exosomal miRNAs under non-stimulated (CK-) or cytokine-stimulated (CK+), in the presence or absence of GW4869 and/or manumycin A (Manu A) (2x inhib.), was analysed by absolute quantification PCR (see Materials and Methods). The relative abundance (x-fold) of each candidate exosomal miRNA was determined based on the total RNA copy numbers/ng of total cellular RNA in each sample relative to that in CK- sample without inhibitor. Data represent mean ± SD of a single analysis (pool of three samples) in three replicates.

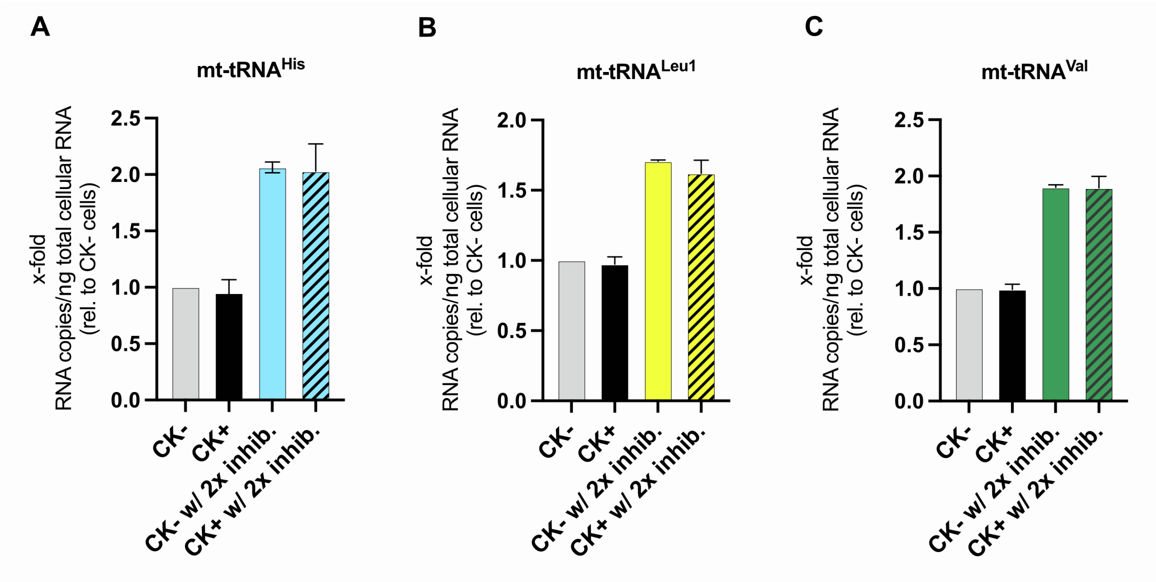

Figure S10. Comparison of cellular mt-tRNA (mt-tRNA^{His}, mt-tRNA^{Leu1} and mt-tRNA^{Val}) expressions in RPTECs under different conditions. A-C) The expression of three candidate exosomal mt-tRNAs under non-stimulated (CK-) or cytokine-stimulated (CK+), in the presence or absence of GW4869 and manumycin A (Manu A) (2x inhib.), was analysed by absolute quantification PCR (see Materials and Methods). The relative abundance (x-fold) of each candidate exosomal miRNA was determined based on the total RNA copy numbers/ng of total cellular RNA in each sample relative to that in CK- sample without inhibitor. Data represent mean ± SEM of two independent experiments.

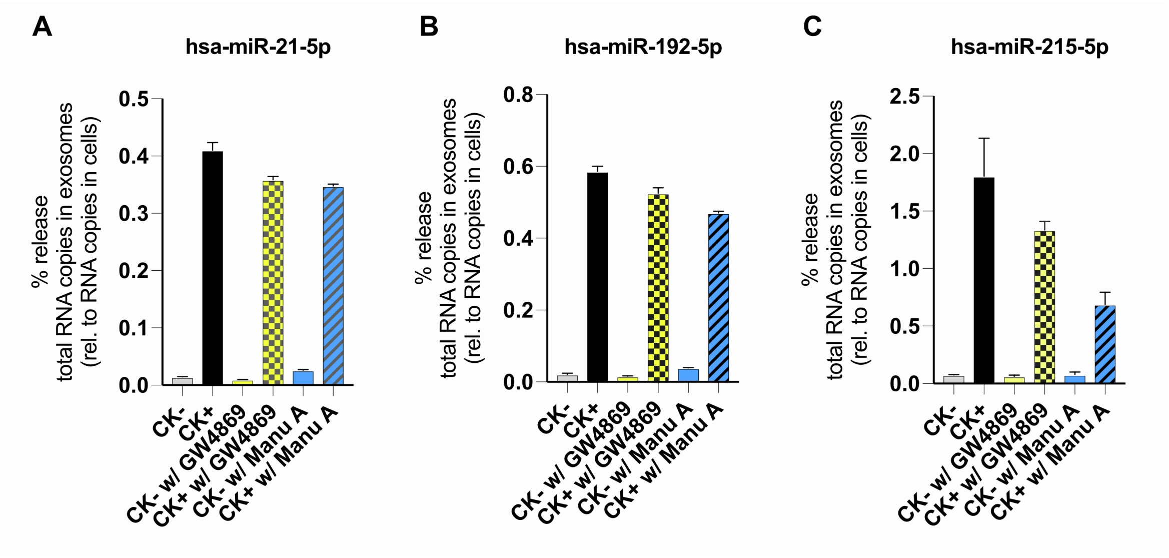

Figure S11. Effect of GW4869 and manumycin A on the cytokine-induced release of exosomal miRNAs, hsa-miR-21-5p, -215-5p and -192-5p from RPTECs. A-C-) RT-qPCR analysis of dysregulated exosomal miRNAs, hsa-miR-21-5p, -215-5p and -192-5p, derived from non-stimulated (CK-) or cytokine-stimulated (CK+) RPTECs treated with individual exosome inhibitor, GW4869 (5 μM) or manumycin A (Manu A) (250 nM). The RNA copy numbers of exosomal miRNAs were analysed by absolute quantification PCR using a synthetic miRNA (i.e. hsa-miR-21-5p, -215-5p or -192-5p) as a standard curve. The percentage (%) release of each dysregulated exosomal miRNA was calculated from the total cDNA copy number in exosomes over the total cDNA copy number in cells. Data represent mean ± SD of a single analysis, which consists of three independent samples pooled together.

Table S1. List of reference genes for normalization. Data represent mean and standard deviation (SD) of Ct values.

miRNA ID	Exosome samples		Cell samples		Urine samples	
	Mean	SD	Mean	SD	Mean	SD
hsa-let-7a-5p	27.22	2.34	32.09	0.47	26.78	1.57
hsa-let-7b-5p	26.46	1.93	29.86	0.69	27.36	1.66
hsa-miR-103a-3p	31.61	3.48	36.97	0.03	28.20	1.61
hsa-miR-191-5p	27.16	3.13	33.12	0.98	29.88	1.60
hsa-miR-26a-5p	24.95	3.21	29.64	0.51	27.49	1.88
hsa-miR-92a-3p	26.94	2.15	31.59	0.46	28.63	1.42

Table S2. Differentially abundant exosomal miRNAs in cytokine-stimulated (CK+) (n=3) and non-stimulated (CK-) (n=3) RPTECs.

miRNA ID	Fold change ($FC=2^{-\Delta\Delta Ct}$) CK+ / CK-	p-value
hsa-miR-31-3p	4.6	0.0318
hsa-miR-218-5p	3.4	0.0384
hsa-miR-146a-5p	3.1	0.0162
hsa-miR-130a-3p	3.1	0.0338
hsa-miR-421	2.9	0.0086
hsa-miR-21-5p	2.5	0.0011
hsa-miR-146b-5p	2.5	0.0088
hsa-miR-625-3p	2.5	0.0314
hsa-miR-126-5p	2.4	0.0393
hsa-miR-192-5p	2.3	0.0204
hsa-miR-215-5p	2.2	0.0227
hsa-miR-30b-5p	2.2	0.0327
hsa-miR-196a-5p	2.1	0.0405
hsa-miR-155-5p	2.1	0.0085
hsa-miR-30a-5p	2.0	0.0485
hsa-miR-151a-3p	2.0	0.0085
hsa-miR-200b-3p	1.9	0.0055
hsa-miR-200c-3p	1.9	0.0108
hsa-miR-361-5p	1.9	0.0237
hsa-miR-126-3p	1.9	0.0013
hsa-miR-30c-5p	1.8	0.0135
hsa-miR-30d-5p	1.8	0.0374
hsa-miR-182-5p	1.7	0.0149
hsa-miR-23b-3p	1.7	0.0011
hsa-miR-151a-5p	1.7	0.0474
hsa-miR-194-5p	1.6	0.0308
hsa-let-7a-5p	0.6	0.0346
hsa-miR-10b-5p	0.6	0.0392
hsa-let-7c-5p	0.4	0.0227
hsa-miR-663a	0.2	0.0071

Table S3. Analysis of most highly expressed exosomal RNAs from non-stimulated (CK-) and cytokine-stimulated (CK+) RPTECs by RNA-sequencing. Highlighted ncRNAs indicate the overlapping ncRNAs between CK- and CK+.

CK-			CK+		
**Gene ID**	**Gene_biotype**	**Average of normalized counts**	**Gene ID**	**Gene_biotype**	**Average of normalized counts**
**MT-RNR2**	Mt_rRNA	14699	**AC073140.2**	lncRNA	14346
**AC073140.2**	lncRNA	10499	**MT-RNR2**	Mt_rRNA	7350
**MT-TM**	Mt_tRNA	7740	**RNY1**	misc_RNA	6458
**MT-RNR1**	Mt_rRNA	6385	**SNORD100**	snoRNA	4673
**SNORD100**	snoRNA	5058	**MIR30A**	miRNA	4306
**RNY1**	misc_RNA	4637	**MT-RNR1**	Mt_rRNA	2160
MT-TS2	Mt_tRNA	3125	**MIR10B**	miRNA	1784
**MIR30A**	miRNA	3016	**MIR31**	miRNA	1723
MT-TL2	Mt_tRNA	2030	**VTRNA1-1**	misc_RNA	1464
MT-TH	Mt_tRNA	1864	**GAS5**	lncRNA	1412
**VTRNA1-1**	misc_RNA	1741	**MT-TM**	Mt_tRNA	1401
**MIR10B**	miRNA	1626	MIR200A	miRNA	1210
MT-TV	Mt_tRNA	1317	**SNORD6**	snoRNA	1138
**MIR31**	miRNA	1217	**SNORD69**	snoRNA	1074
**SNORD69**	snoRNA	1129	MIR200B	miRNA	951
**GAS5**	lncRNA	1040	MIR221	miRNA	926
**SNORD6**	snoRNA	1001	MIR30D	miRNA	888
RNY4	misc_RNA	960	MIRLET7G	miRNA	853
MIR3591	miRNA	925	SNORD99	snoRNA	762
RNY3	misc_RNA	848	MIR29A	miRNA	746

Table S4. Differentially abundant exosomal mtRNAs from cytokine-stimulated relative to non-stimulated RPTECs.

Gene ID	Log2 FC	p-value	adjusted p-value
MT-TV	-2.82	1.657E-11	6.349E-08
MT-TI	-2.57	7.755E-07	3.301E-04
MT-TS2	-2.40	1.066E-09	1.361E-06
MT-TR	-2.34	4.031E-07	2.574E-04
MT-TL2	-2.25	3.581E-10	6.859E-07
MT-TH	-2.14	1.644E-08	1.575E-05
MT-TM	-2.09	2.244E-07	1.719E-04
MT-TL1	-2.09	5.379E-07	2.944E-04
MT-TF	-2.02	6.438E-07	3.083E-04
MT-TP	-1.97	1.804E-06	6.912E-04
MT-TD	-1.94	3.839E-05	1.337E-02
MT-TC	-1.88	5.093E-05	1.626E-02
MT-TY	-1.74	1.329E-04	3.831E-02
MT-ATP6	-1.64	1.808E-04	4.347E-02
MT-ND4	-1.54	1.400E-04	3.831E-02
MT-RNR1	-1.27	1.816E-04	4.347E-02

Table S5. Overlap of dysregulated exosomal miRNAs in the CKD group (Next generation Sequence Analysis of clinical samples)[19] and RPTEC CK+ group (diseased cells) (RT-qPCR analysis using LNA miRNA miRNome Human Panel I).

CKD stage	miRNA ID	Clinical samples (CKD vs. Healthy control)		RPTECs (CK+ vs. CK-)	
		Log2 Fold Change	p-value adjusted	Fold change	p-value
I	hsa-miR-215-5p	3.7	0.0122	2.2	0.0227
II	hsa-miR-126-5p	3.7	0.0023	2.4	0.0393
	hsa-miR-126-3p	2.2	0.0458	1.9	0.0013
	hsa-miR-21-5p	1.5	0.0217	2.5	0.0011
	hsa-miR-215-5p	1.3	0.0931	2.2	0.0227
	hsa-miR-192-5p	1.2	0.0531	2.3	0.0204
	hsa-miR-31-3p	1.2	0.0789	4.6	0.0318
	hsa-miR-23b-3p	0.6	0.0789	1.7	0.0011
III	hsa-miR-215-5p	3.8	0.0146	2.2	0.0227
	hsa-miR-146a-5p	2.3	0.0110	3.1	0.0162
IV	hsa-miR-215-5p	3.5	0.0270	2.2	0.0227
	hsa-miR-192-5p	2.7	0.0349	2.3	0.0204
	hsa-miR-146a-5p	1.5	0.0864	3.1	0.0162
	hsa-miR-23b-3p	0.6	0.0991	1.7	0.0011

Table S6. Overlap of dysregulated exosomal mt-tRNAs in the CKD group[19] and RPTEC CK+ group (diseased cells).

CKD stage	mt-tRNA ID	Clinical samples (CKD vs. Healthy control)		RPTECs (CK+ vs. CK-)	
		Log2 Fold Change	p-value adjusted	Log2 Fold Change	p-value adjusted
I	MT-TC	-3.1	9.41E-05	-1.88	1.63E-02
	MT-TI	-2.9	3.84E-03	-2.57	3.30E-04
	MT-TL2	-2.9	7.41E-04	-2.25	6.86E-07
	MT-TS2	-2.7	3.84E-03	-2.40	1.36E-06
	MT-TL1	-2.6	6.40E-04	-2.09	2.94E-04
	MT-TH	-2.5	6.14E-04	-2.14	1.57E-05
	MT-TM	-2.5	7.02E-04	-2.09	1.72E-04
	MT-TV	-2.5	1.12E-03	-2.82	6.35E-08
	MT-TP	-2.4	7.33E-03	-1.97	6.91E-04
	MT-TY	-2.2	2.00E-02	-1.74	3.83E-02
	MT-TR	-2.0	2.90E-02	-2.34	2.57E-04
	MT-TF	-1.9	7.42E-03	-2.02	3.08E-04
	MT-TD	-1.5	7.78E-02	-1.94	1.34E-02
II	MT-TD	-1.9	4.96E-02	-1.94	1.34E-02
	MT-TR	-1.8	7.85E-02	-2.34	2.57E-04
	MT-TC	-1.7	2.97E-02	-1.88	1.63E-02
	MT-TP	-1.6	8.98E-02	-1.97	6.91E-04
	MT-TL2	-1.4	9.94E-02	-2.25	6.86E-07
	MT-TH	-1.2	8.46E-02	-2.14	1.57E-05
III	MT-TD	-3.2	2.91E-03	-1.94	1.34E-02
	MT-TL1	-2.6	1.85E-03	-2.09	2.94E-04
	MT-TL2	-2.4	6.56E-03	-2.25	6.86E-07
	MT-TC	-1.9	1.30E-02	-1.88	1.63E-02
	MT-TM	-1.9	9.93E-03	-2.09	1.72E-04
	MT-TP	-1.8	5.83E-02	-1.97	6.91E-04
	MT-TI	-1.8	6.88E-02	-2.57	3.30E-04
	MT-TH	-1.8	1.56E-02	-2.14	1.57E-05
	MT-TF	-1.4	6.31E-02	-2.02	3.08E-04
	MT-TV	-1.3	8.98E-02	-2.82	6.35E-08
IV	MT-TC	-2.0	2.04E-03	-1.88	1.63E-02
	MT-TP	-1.6	7.16E-02	-1.97	6.91E-04
	MT-TL1	-1.6	4.15E-03	-2.09	2.94E-04
	MT-TY	-1.8	4.17E-02	-1.74	3.83E-02

Table S7. List of primers and template sequences.

#	Name	Sequences (5' to 3')	Assay	Source
1	hsa-miR-21-5p	UAGCUUAUCAGACUGAUGUUGA	PCR	YP00204230 (Qiagen)
2	hsa-miR-192-5p	CUGACCUAUGAAUUGACAGCC	PCR	YP00204099 (Qiagen)
3	hsa-miR-215-5p	AUGACCUAUGAAUUGACAGAC	PCR	YP00204598 (Qiagen)
4	hsa-miR-194-5p	UGUAACAGCAACUCCAUGUGGA	PCR	YP00204080 (Qiagen)
5	hsa-miR-126-3p	UCGUACCGUGAGUAAUAAUGCG	PCR	YP00204227 (Qiagen)
6	hsa-miR-146a-5p	UGAGAACUGAAUUCCAUGGGUU	PCR	YP00204688 (Qiagen)
7	hsa-miR-21-5p	TAGCTTATCAGACTGATGTTGA	PCR	IDT
8	hsa-miR-192-5p	CTGACCTATGAATTGACAGCC	PCR	IDT
9	hsa-miR-215-5p	ATGACCTATGAATTGACAGAC	PCR	IDT
10	Universal primer rev	GAATCGAGCACCAGTTACGCA	PCR	IDT
11	hsa-miR-21a-5p template w/ tag	GAATCGAGCACCAGTTACGCATTTTGCTCAACATCAGTCTGATAAGCTA	PCR	IDT
12	hsa-miR-192-5p template w/ tag	GAATCGAGCACCAGTTACGCATTTTGCGGCTGTCAATTCATAGGTCAG	PCR	IDT
13	hsa-miR-215-5p template w/ tag	GAATCGAGCACCAGTTACGCATTTTGCGTCTGTCAATTCATAGGTCAT	PCR	IDT
14	SNORD100 fwd	TACATGATGACAACTGGCTCC	PCR	IDT
15	mt-tRNA^{His} fwd	GATTGTGAATCTGACAACAGAGG	PCR	IDT
16	mt-tRNA^{Leu2} fwd	CCATTGGTCTTAGGCCCCA	PCR	IDT
17	mt-tRNA^{Val} fwd	GCTTAACACAAAGCACCCAACT	PCR	IDT
18	mt-tRNA^{Phe} fwd	CCTCCTCAAAGCAATACACTGA	PCR	IDT
19	mt-tRNA^{Leu1} fwd	GAGCCCGGTAATCGCATAAAAC	PCR	IDT
20	mt-tRNA^{His} fwd_w/ T7 promoter sequence	AGGTAATACGACTCACTATAGGGTAAATATAGTTTAACC	PCR	IDT
21	mt-tRNA^{Leu1} fwd_w/ T7 promoter sequence	AGGTAATACGACTCACTATAGGGTTAAGATGGCAGAGCC	PCR	IDT
22	mt-tRNA^{Val} fwd_w/ T7 promoter sequence	AGGTAATACGACTCACTATAGGCAGAGTGTAGCTTAACAC	PCR	IDT
23	mt-tRNA^{His} rev	GGTAAATAAGGGGTCGTAAGC	PCR	IDT
24	mt-tRNA^{Leu1} rev	TGTTAAGAAGAGGAATTGAACC	PCR	IDT
25	mt-tRNA^{Val} rev	TCAGAGCGGTCAAGTTAAG	PCR	IDT
26	mt-tRNA^{His} template	GTAAATATAGTTTAACCAAAACATCAGATTGTGAATCTGACAACAGAGGCTTACGACCCCTTATTTACC	PCR	IDT
27	mt-tRNA^{Leu1} template	GTTAAGATGGCAGAGCCCGGTAATCGCATAAAACTTAAAACTTTACAGTCAGAGGTTCAATTCCTCTTCTTAACA	PCR	IDT
28	mt-tRNA^{Val} template	CAGAGTGTAGCTTAACACAAAGCACCCAACTTACACTTAGGAGATTTCAACTTAACTTGACCGCTCTGA	PCR	IDT
29	mt-tRNA^{His} rev	CCTCTGTTGTCAGATTCACAATC	northern blot	IDT
30	mt-tRNA^{Leu2} rev	TGGGGCCTAAGACCAATGG	northern blot	IDT
