# Supplementary material for: Exosomal mitochondrial tRNAs and miRNAs as potential predictors of inflammation in renal proximal tubular epithelial cells
Source: Mol Ther Nucleic Acids. 2022 May 4;28:794–813. doi: 10.1016/j.omtn.2022.04.035 (PMC9136061; doi:10.1016/j.omtn.2022.04.035)
Supplement: Document S2. Article plus supplemental information [file mmc2.pdf]

Molecular Therapy
Nucleic Acids
Original Article

Exosomal mitochondrial tRNAs and miRNAs as potential predictors of inflammation in renal proximal tubular epithelial cells

Glory Ranches,[1] Maximilian Zeidler,[2] Roman Kessler,[1] Martina Hoelzl,[1] Michael W. Hess,[3] Jonathan Vosper,[4] Paul Perco,[5] Herbert Schramek,[5] Kai K. Kummer,[2] Michaela Kress,[2] Anne Krogsdam,[6] Michael Rudnicki,[5] Gert Mayer,[5] and Alexander Huettenhofer[1]

[1]Division of Genomics and RNomics, Biocenter, Medical University of Innsbruck, Innsbruck 6020, Austria; [2]Institute of Physiology, Medical University of Innsbruck, Innsbruck 6020, Austria; [3]Institute of Histology and Embryology, Medical University of Innsbruck, Innsbruck 6020, Austria; [4]Division of Medical Biochemistry, Biocenter, Medical University of Innsbruck, Innsbruck 6020, Austria; [5]Department of Internal Medicine IV (Nephrology and Hypertension), Medical University of Innsbruck, Innsbruck 6020, Austria; [6]Division of Bioinformatics, Biocenter, Medical University of Innsbruck, Innsbruck 6020, Austria

Exosomes have emerged as a valuable repository of novel biomarkers for human diseases such as chronic kidney disease (CKD). From a healthy control group, we performed microRNA (miRNA) profiling of urinary exosomes and compared it with a cell culture model of renal proximal tubular epithelial cells (RPTECs). Thereby, a large fraction of abundant urinary exosomal miRNAs could also be detected in exosomes derived from RPTECs, indicating them as a suitable model system for investigation of CKD. We subsequently analyzed exosomes from RPTECs in pro-inflammatory and pro-fibrotic states, mimicking some aspects of CKD. Following cytokine treatment, we observed a significant increase in exosome release and identified 30 dysregulated exosomal miRNAs, predominantly associated with the regulation of pro-inflammatory and pro-fibrotic-related pathways. In addition to miRNAs, we also identified 16 dysregulated exosomal mitochondrial RNAs, highlighting a pivotal role of mitochondria in sensing renal inflammation. Inhibitors of exosome biogenesis and release significantly altered the abundance of selected candidate miRNAs and mitochondrial RNAs, thus suggesting distinct sorting mechanisms of different non-coding RNA (ncRNA) species into exosomes. Hence, these two exosomal ncRNA species might be employed as potential indicators for predicting the pathogenesis of CKD and also might enable effective monitoring of the efficacy of CKD treatment.

INTRODUCTION

Chronic kidney disease (CKD) is a pathological condition marked by structural abnormalities of the kidney coupled with dysfunction that progresses over time. The severity of CKD is defined by five stages (stages G1–G5) on the basis of glomerular filtration rate (GFR), either estimated (eGFR) or measured (mGFR), and the extent of albuminuria (stages A1–A3).[1,2] In 2017, 697.5 million cases of CKD were recorded worldwide, which clearly indicates that CKD is a major global health problem.[3]

Although eGFR and albuminuria reflect glomerular functional abnormalities, these indicators are insufficient to draw precise conclusions concerning the pathophysiology of CKD. In addition to glomerular abnormalities, tubulointerstitial damage is similarly important[4] and represents a final common pathway, leading to loss of kidney function as indicated by a decrease in the GFR.[5] In addition, renal proximal tubule epithelia not only are susceptible to injury but importantly drive the progression of injury per se via the induction of a pro-inflammatory and pro-fibrotic milieu.[6,7] Therapeutic interventions directed at signaling events originating from the proximal tubules may therefore prevent progression of CKD.

Since renal proximal tubular epithelial cells (RPTECs) are susceptible to kidney insults and play a vital role in the initiation and progression of CKD,[6,7] they represent a suitable cellular model system for kidney disease.[6,8] Previously, it has been shown that a human RPTEC cell line, immortalized by stable overexpression of the catalytic subunit of human telomerase (TERT1),[9] resembles the proximal tubular cell type found *in vivo* with respect to its morphological characteristics and functional properties.[9,10] Immortalized RPTECs recapitulate many characteristic functions of this cell type *in vivo*.[9] Indeed, Wieser and co-workers previously have shown numerous phenotypic similarities between RPTEC/TERT1 cells and wild-type RPTECs.[9] Furthermore, stimulation of such proximal tubular cells by pro-inflammatory and pro-fibrotic cytokines such as interleukin (IL)-1β,

Received 16 December 2021; accepted 28 April 2022;
https://doi.org/10.1016/j.omtn.2022.04.035.

Correspondence: Alexander Hüttenhofer, Division of Genomics and RNomics, Biocenter, Medical University of Innsbruck, Innsbruck 6020, Austria.
E-mail: alexander.huettenhofer@i-med.ac.at
Correspondence: Glory Ranches, Division of Genomics and RNomics, Biocenter, Medical University of Innsbruck, Innsbruck 6020, Austria.
E-mail: glory.ranches@i-med.ac.at
Correspondence: Gert Mayer, Department of Internal Medicine IV (Nephrology and Hypertension), Medical University of Innsbruck, Innsbruck 6020, Austria.
E-mail: gert.mayer@i-med.ac.at

 © 2022 The Author(s).
This is an open access article under the CC BY license (http://creativecommons.org/licenses/by/4.0/).

oncostatin M (OSM), and transforming growth factor (TGF)-β1 promotes upregulation of the pro-inflammatory gene C-C motif chemokine ligand 2 (*CCL2*).[11,12] These cytokines induce a local inflammatory response and/or affect the proximal tubular cell phenotype, thus contributing to the progression of tubulointerstitial fibrosis.[11,13]

Several studies also suggest that the progression of CKD is more closely linked with tubule-interstitial damage (malfunction) rather than with glomerular damage.[14] In support of this idea, Grgic and colleagues have shown that acute injury to proximal renal tubules is sufficient to generate a full spectrum of pathological changes associated with progressive CKD, based on a mouse model of kidney injury.[6]

Recent findings obtained using a mouse model of kidney injury have led to the discovery of important pathological mechanisms associated with RPTECs that contribute to the development and progression of CKD.[6,15] However, specific transcriptional responses linked to this pathway of CKD pathogenesis remain elusive, especially in the context of regulation of cytokine-mediated proximal tubular inflammation. In addition, despite a high degree of protein-coding conservation between human and mouse, the transcriptional response observed in mouse inflammatory disease models differs significantly from that of human inflammatory diseases.[16] A human-cell-based approach in gene expression profiling may bridge the gap between animal models and human diseases, and may help to define disease-associated gene signatures specific to a certain cell type (e.g., RPTECs).

Exosomes are emerging as one of the most promising sources for identification of molecular markers specific to a disease state of cells since they are released by cells into biofluids (e.g., plasma and urine) *in vivo* and cell culture medium *in vitro*, and thus can be exploited as a "liquid biopsy" for the diagnosis of pathological conditions.[17–20] Exosomes are membrane-bound extracellular vesicles (EVs) with a size range from 30 to 150 nm in diameter and are formed within multivesicular bodies (MVBs) during the endosomal sorting process.[21,22] Their formation is mediated by the endosomal sorting complex required for transport (ESCRT) machinery involving specific proteins such as ALG-2-interacting protein X (ALIX) and tumor susceptibility gene 101 protein (TSG101).[23–26] Exosomes can also be generated independently of the ESCRT pathway via the neural sphingomyelinase (nSMAse)-dependent pathway, which involves hydrolysis of sphingolipids by nSMase to generate ceramide, a critical component for exosome formation.[25] Hence, biogenesis and release of exosomes can be inhibited by nSMase inhibitors such as GW4869 and manumycin A, which also acts as a farnesyltransferase inhibitor.[25,27–29] Inhibition of farnesyltransferase prevents RAS activation, which is implicated in exosome biogenesis through the ESCRT pathway. Manumycin A attenuates the expression of ESCRT-0 proteins Hrs, ALIX, and Rab27a, which are components of exosome biogenesis and secretion, via targeted inhibition of the Ras/Raf/ERK1/2 signaling.[29] Blockage of both pathways results in a strong downregulation of exosome biogenesis and release.[29,30]

It has been shown previously that exosomes harbor a repertoire of molecules such as nucleic acids (e.g., RNAs and DNAs), as well as lipids and proteins, which may reflect the pathological condition of their parental cells.[19,22,31] They mediate cell-to-cell communication in diverse biological scenarios and have functions in the maintenance of cellular integrity, tumor progression, and the immune response.[32–34] The cargo in exosomes and other EVs are protected from enzymatic digestion by proteases or nucleases[35] and, as such, they might be more stable compared with protein- or exosome-devoid RNAs or DNAs. In recent years, several potential exosomal non-coding RNAs (ncRNAs; e.g., microRNAs [miRNAs] and long ncRNAs [lncRNAs]) associated with kidney disease have been identified from urinary exosomes or kidney tissue through RNA sequencing (RNA-seq) and miRNA profiling approaches.[19,20,36]

In this study, we identified RPTECs as a potential major source of urinary exosomes in healthy humans. We next employed a cell culture model system for CKD derived from RPTECs that mimic a pro-inflammatory and pro-fibrotic condition (disease state) in order to determine RPTEC-specific exosomal RNA signatures associated with inflammation and fibrosis. Through a combinatorial approach employing miRNA profiling and RNA-seq analysis, we identified several dysregulated exosomal miRNAs as well as mtRNAs (predominantly mitochondrial tRNAs [mt-tRNAs]). Hence, proximal tubular epithelial cells represent a potential source of kidney inflammatory transcript markers and might thus represent an interesting target for the evaluation of novel CKD therapies.

RESULTS

Comparison between exosomal miRNAs from an RPTEC *in vitro* model and urinary exosomal miRNAs from a healthy control group shows significant overlap

Recent studies have shown that exosomes derived from human urine may mirror a specific pathological condition (e.g., CKD and diabetic kidney disease).[19,37,38] However, it has not yet been demonstrated if these urinary exosomal markers directly represent pathological changes to the kidney or whether they are derived from other tissues or cells linked to the disease (e.g., invading inflammatory cells). Since RPTECs are a primary target of kidney injury and contribute to the development and progression of CKD,[14] we investigated whether urinary exosomal miRNAs might indeed be derived from RPTECs.

To this end, we isolated exosomes from non-stimulated/control RPTEC (designated as CK−) cell culture medium and urine samples obtained from healthy individuals as controls (healthy controls [HCs]) using an ultracentrifugation method (Figures S1A–S1C). The sample quality was evaluated by negative-stain transmission electron microscopy (TEM)[39] (Figures S2A and S2B) and western blotting for the tumor suppressor gene 101 (TSG101), a known exosomal marker (Figures S2C and S2D).[40] The vast majority of exosomes/EVs in both samples were roughly 24 nm in diameter.

We next performed miRNA profiling by employing the miRCURY locked nucleic acid (LNA) miRNA miRNome Human PCR Panel I

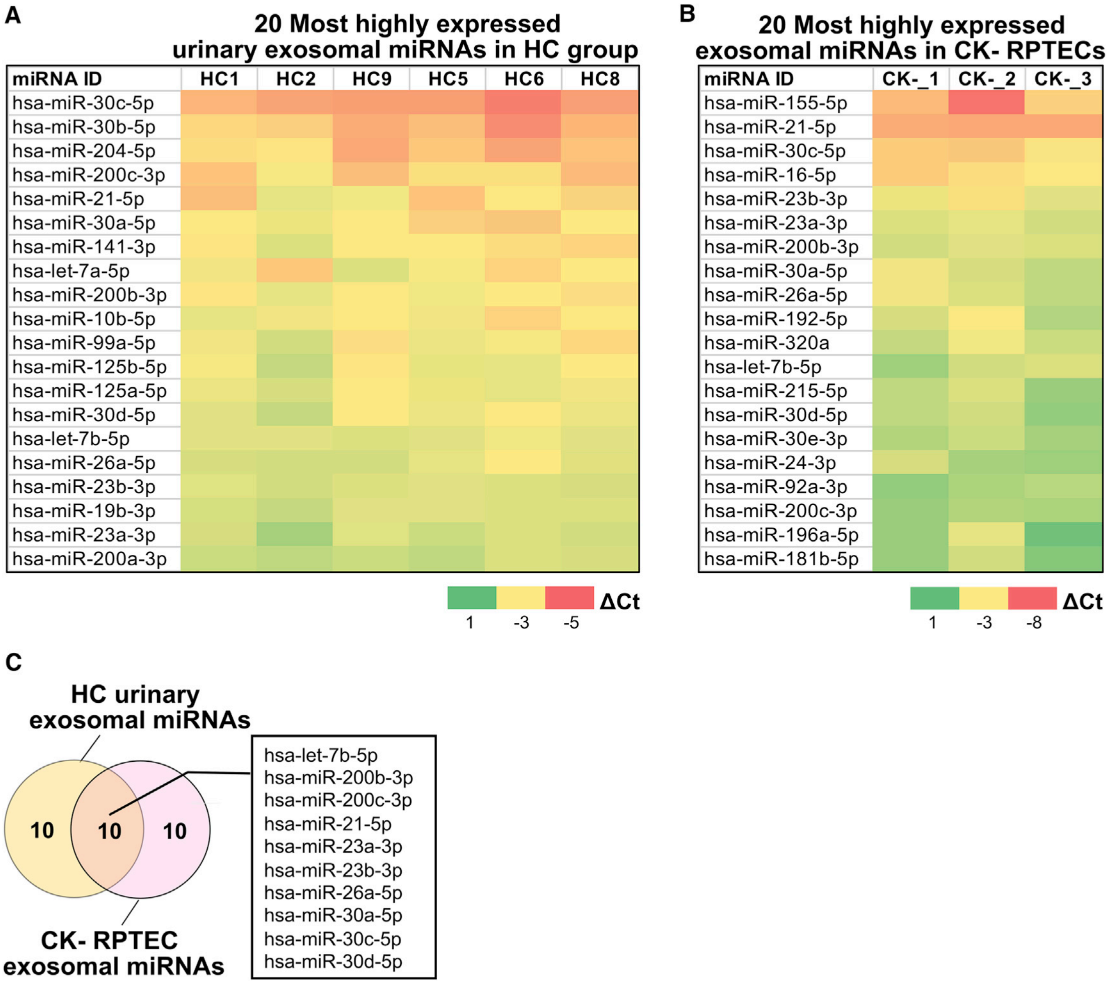

Figure 1. Urinary exosomal miRNAs that are potentially derived from RPTECs

(A) Top 20 most abundant exosomal miRNAs from healthy control (HC) group (n = 6) and (B) non-stimulated RPTECs (CK−) (n = 3). Heatmap colors indicate the delta Ct (ΔCt) value of each target miRNA. Red indicates lower ΔCt (higher gene expression) and green indicates higher ΔCt (lower gene expression). (C) Overlap between the 20 most abundant exosomal miRNAs derived from CK− RPTECs and that from urine of HC group.

containing 368 miRNA species. miRNA profiles were analyzed, and the abundance of each miRNA was determined using six reference genes (hsa-let-7a-5p, hsa-let-7b-5p, hsa-miR-191-5p, hsa-miR-26a-5p, hsa-miR-92a-3p, and hsa-miR-103a-3p) for normalization. These miRNAs were found to be stably expressed with low variability for all the samples investigated (e.g., RPTECs and exosomes and urine samples from HC group) (Table S1); in addition, these miRNAs have previously been reported to be suitable reference genes for miRNA quantification.[41–44] Hsa-miR-26a-5p, hsa-miR-92a-3p, and hsa-miR-103a-3p were also reported to serve as suitable reference genes for human renal tissue and RPTECs under commonly applied conditions (e.g., basal conditions and stimulation with glucose, TNF-α, and TGF-β1).[45]

Following normalization, we analyzed the delta Ct (ΔCt) values of exosomal miRNAs present in each group and determined that 30% of the 368 miRNAs analyzed were present in RPTEC exosome samples, whereas 34% were identified in HC samples. Subsequently, we determined the 20 most abundant urinary exosomal miRNAs in the HC group and compared the results with the RPTEC *in vitro* model employing the non-stimulated CK− group (Figures 1A and 1B). This analysis showed that about half of these exosomal miRNAs are shared between RPTEC CK− and urinary HC groups (Figure 1C), indicating that a significant number of urinary exosomes are potentially derived from kidney epithelial cells. This is in agreement with previously published results.[46]

Cytokine-induced release of RPTEC-derived exosomes

In the CKD RPTEC model system, we employed pro-inflammatory and pro-fibrotic cytokines, namely IL-1β, OSM, and TGF-β1, to transform RPTECs into a state mimicking certain aspects of a CKD phenotype, as previously demonstrated.[11,47] To this end, RPTECs were differentiated and starved prior to the addition of cytokines

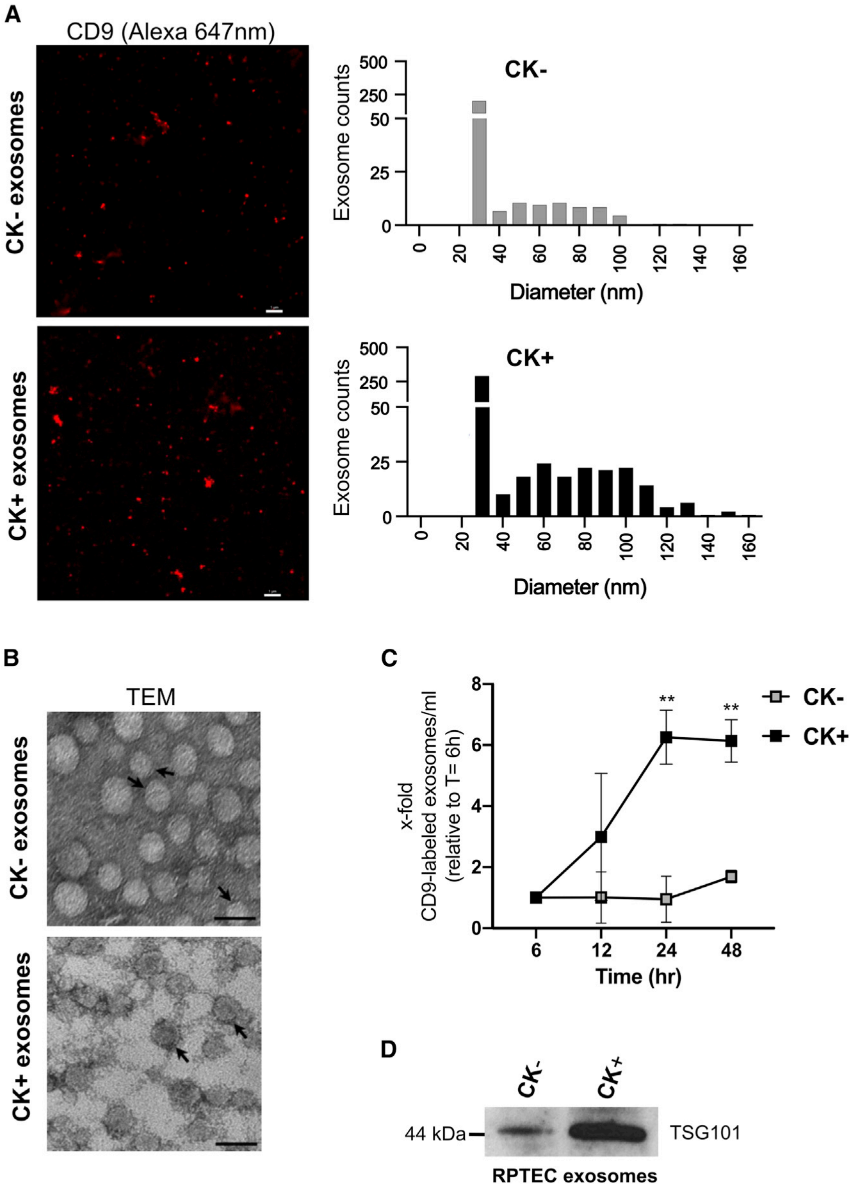

Figure 2. Characterization of exosomes derived from RPTECs

(A) 2D STORM imaging of anti-CD9-labeled (Alexa 647 nm) exosomes from non-stimulated RPTECs (CK−) versus cytokine-stimulated RPTECs (CK+) (see section "materials and methods"). Photos are representative images. Scale bar, 1,000 nm. (B) Transmission electron microscopy (TEM) of exosomes derived from CK− versus CK+ RPTECs; arrows point to the exosome membrane bilayer. Scale bar, 50 nm. C) Time course analysis of exosomal release from CK− and CK+ RPTECs using Alexa (647 nm)-labeled CD9 antibody for exosome immunolabeling (see section "materials and methods"). Statistical analysis was performed using two-way ANOVA with multiple comparison test and Bonferroni correction (GraphPad Prism 8.0.1). Results are presented as mean ± SEM of two independent experiments. **$p < 0.01$. (D) Immunoblot analysis of TSG101 in exosome lysates of either CK− or CK+ sample. The image is a representative of three independent experiments.

(designated as CK−). Exosomes that were secreted into the culture medium from cytokine-stimulated/diseased cells (designated as CK+) and CK− RPTECs were isolated and purified by ultracentrifugation (Figures S1A and S1B).

In order to determine the entire size distribution of exosomes in each sample, we employed a high-resolution imaging technique, referred to as stochastic optical reconstruction microscopy (STORM), that is able to rapidly detect EVs down to 20–30 nm in size with high sensitivity.[48,49] In this assay, we immunostained exosomes by employing a photo-switchable probe, i.e., an Alexa (647 nm)-labeled antibody, directed against CD9, a tetraspanin, which represents a validated exosomal marker.[50] Quantification of the CD9 fluorescence signal revealed an increased level of CD9 in the CK+ sample, relative to the CK− sample (Figure 2A). The size distribution of CD9-positive CK+ exosomes was determined to be in the range of 27–142 nm, whereas a size distribution of 27–97 nm was observed for CK− exosome samples. The majority of isolated exosomes in both samples has a diameter of 30 nm but an increase in the number of exosome populations was observed in CK+ compared with that of CK−. We additionally analyzed samples by TEM as a complementary approach. RPTEC-derived EVs considered as exosomes based on their morphology exhibited a size distribution ranging from 15 to 46 nm for CK+ (24.74, SD 5.25) and 13 to 44 nm for CK− (24.66, SD 5.22; Figure 2B). Taken together, our results are consistent with the reported size range of *bona fide* exosomes (i.e., from 20 to 140 nm).[51]

In order to investigate the effect of cytokines in mediating RPTEC release of exosomes, we performed immunofluorescence analysis of the exosomal tetraspanin CD9 marker in a time course experiment. Following cytokine stimulation, RPTEC exosomes isolated at different time points (6–48 h) were immunostained with an anti-CD9 (Alexa 647 nm) antibody and the CD9 fluorescence signal was analyzed using a plate reader. Based on the average CD9 fluorescence signal, a significant increase in CD9 levels was observed in CK+ exosome samples ($p < 0.05$) after 24 h and 48 h, compared with the CK− samples (Figure 2C). The CD9 increase in CK+ exosome samples was apparent after 12 h (3-fold). The number of CD9-positive exosomes was further increased at 24 h (6-fold) and at 48 h (6-fold) relative to the baseline at 6 h, whereas CD9 levels remained unchanged in CK− exosome samples (1.0- to 1.6-fold) throughout the entire time course experiment

(Figure 2C), suggesting that cytokine stimulation induces exosome release.

To further validate additional key features of exosomal vesicles, we performed western blot analysis of the total exosome lysate and analyzed the protein level of the tumor suppressor gene 101 (TSG101).[40] In the CK+ exosome sample, TSG101 protein levels were found to be significantly elevated compared with the CK− exosomal sample (Figure 2D).

In order to analyze the RNA content in each exosome sample, we isolated total RNA from exosome pellets as well as from their originating cells (i.e., CK− or CK+ cells, respectively). To determine the size distribution of exosomal RNA species, we analyzed the total RNA from exosomes and cells using a Bioanalyzer, employing small RNA (Figures S3A and S3B) and RNA pico chips (Figures S3C and S3D). Both analyses showed that the RNA content was increased in the CK+ exosome samples compared with the CK− exosome samples. The highest RNA abundance was observed for RNAs exhibiting a size below 200 nt, whereas fewer RNA species were observed exhibiting sizes above 200 nt. This observation indicates that the majority of RPTEC-derived exosomes indeed contain small RNA species.

Based on these analyses, we thus demonstrate that RPTEC-derived exosomes exhibit *bona fide* exosome structure and properties, and that cytokines (i.e., IL-1β, OSM, and TGF-β1) induce the release of exosomes. This corresponds to the increased levels of CD9, TSG101 protein, and total RNA content in CK+ samples, relative to the CK− samples. Hence, our results indicate that cytokine stimulation of RPTECs triggers an increased release of exosomes compared with non-stimulated RPTECs.

GW4869 and manumycin A alter cytokine-induced exosome release in RPTECs

The biogenesis and sorting of exosomes can be modulated by several nSMase inhibitors such as the cationic molecule GW4869 and natural microbial metabolite manumycin A, which also functions as a Ras farnesyltransferase inhibitor.[52–54] Since previous reports have shown that a combination of these two inhibitors is more efficient than using a single inhibitor,[29] we employed both exosome inhibitors together in subsequent experiments, but at a lower dose, since they significantly reduced cell viability in a few hours following treatment.

We next investigated the influence of these exosome inhibitors in regulating the release of exosomes during inflammation. For this purpose, we determined the levels of the exosomal tetraspanin marker CD9 on exosomes released from CK+ RPTECs relative to that of CK− RPTECs, employing a fluorescence-based microplate assay. Based on the fluorescence intensity signal of CD9, in the presence of GW4869 and/or manumycin A, we observed that CD9 levels were significantly reduced in CK+ exosomes by a factor of 2- to 3-fold ($p < 0.001$), relative to that in CK+ exosomes without GW4869 and/or manumycin A (Figure S4A).

To assess the general effects of GW4869 and/or manumycin A in regulating exosome secretion and release, we also normalized the CD9 level of all samples (CK− with inhibitor and CK+ with or without inhibitor) to the CK− exosome sample (assigned as 1). Among CK− samples, CK− with GW4869 and/or manumycin A samples showed an increased abundance of CD9 (2- to 4-fold), relative to the CK− sample without an exosome inhibitor (Figure S4B). This result indicates that GW4869 and/or manumycin A not only inhibit biogenesis and release of exosomes but may also induce secretion and budding of other CD9-positive EVs/exosomes. In the CK+ exosome samples, the abundance of CD9 was partially decreased in the presence of GW4869 and/or manumycin A by 0.7- to 0.9-fold, compared with that of the CK+ sample alone (Figure S4B). Thus, the reduction of the CD9 ratio in CK+ compared with CK− samples (Figure S4A) suggests that the two reported exosome inhibitors indeed inhibit the secretion and release of exosomes mediated by cytokine induction.

Analysis of abundant exosomal and cellular miRNAs derived from RPTECs

We next investigated the abundance of exosomal miRNAs, which are derived from RPTECs, since miRNAs not only play an essential role in renal repair but may also contribute to the pathophysiology of CKD.[36,55–57] Hence, we performed exosomal miRNA profiling from RPTEC exosomes (i.e., from CK+ and CK− cells, respectively) employing the miRCURY LNA miRNA miRNome Human PCR Panel I. Following the pre-processing of data (see section "materials and methods"), 30% of 368 miRNAs were identified to be present in all RPTEC exosome samples (CK− and CK+).

Based on the analysis of ΔCt values, we identified the 20 most highly expressed exosomal miRNAs present in each group (Figure S5A). Out of 20, 18 of these highly expressed exosomal miRNAs were shared by both groups (i.e., CK− and CK+ exosomes; Figure S5B). The five most abundant exosomal miRNAs found in both CK− and CK+ samples were hsa-miR-21-5p, hsa-miR-155-5p, hsa-miR-30c-5p, hsa-miR-16-5p, and hsa-miR-23b-3p.

In order to investigate whether the release of exosomal miRNA mirrors the abundance of the miRNA species in the cells from which they were derived, we also performed miRNA profiling of total RNA extract from CK+ RPTECs and compared the cellular miRNA profile with its (CK+) exosomal miRNA profile. Interestingly, we found that 19 of the 20 most highly expressed miRNAs were common to both the CK+ cells and exosomes (Figures S5C and S5D). Similar to CK+ exosomal miRNAs, we showed that hsa-miR-21-5p, hsa-miR-155-5p, hsa-miR-30c-5p, hsa-miR-16-5p, and hsa-miR-23b-3p are the most highly expressed miRNAs in CK+ RPTECs (Figure S5C).

Identification of dysregulated exosomal miRNAs from cytokine-stimulated RPTECs

Several studies have shown that miRNAs can be selectively sorted into exosomes[58,59] through interaction with RNA-binding proteins

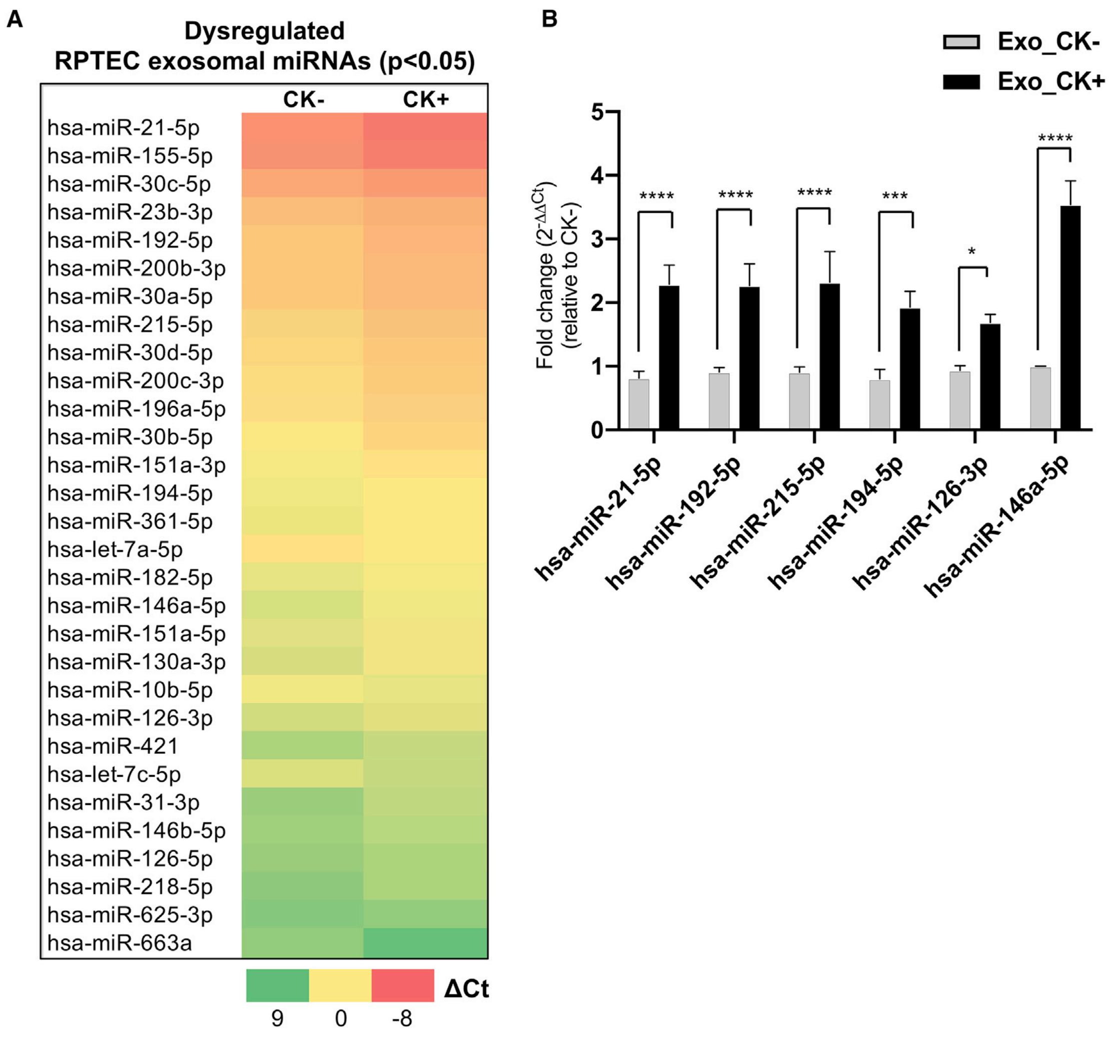

Figure 3. Dysregulated exosomal miRNAs from cytokine-treated RPTECs as potential indicators of inflammatory kidney disease

(A) Identification of dysregulated exosomal miRNAs ($p < 0.05$) derived from non-stimulated (CK−) and cytokine-stimulated (CK+) RPTECs (see section "materials and methods"). Heatmap represents the average ΔCt values of each miRNA in CK− or CK+. Red indicates lower ΔCt (higher gene expression) and green indicates higher ΔCt (lower gene expression). Student's t test was employed for statistical analysis using the ΔCt values obtained from three independent experiments. (B) Validation of six differentially abundant candidate exosomal miRNAs. The relative fold change (FC) of exosomal miRNA expression in cytokine-stimulated condition (Exo_CK+) relative to non-stimulated condition (Exo_CK−) was analyzed using $2^{-\Delta\Delta Ct}$ method. Bar graph represents mean ± SEM of five independent experiments. Two-way ANOVA with Benjamini and Hochberg multiple comparisons was performed for statistical analysis using (GraphPad Prism, 8.0.1). ****$p < 0.0001$, ***$p < 0.001$, *$p < 0.05$.

(RBPs) (e.g., Y-Box Binding Protein 1 [YBX1]),[39,60] Major Vault Protein [MVP],[61] MEX3C,[62] and heterogeneous nuclear ribonucleoprotein A2B1 [hnRNPA2B1]).[63] These studies have further demonstrated that the dysregulation of exosomal miRNAs is associated with several pathologies, including neuroinflammation, diabetes mellitus, and cancer.[64]

By employing a validated *in vitro* cell culture model for CKD, we thus aimed to identify dysregulated exosomal miRNAs signatures that are associated with inflammatory kidney disease and renal fibrosis by employing cytokine-stimulated RPTECs. We first performed expression analysis of exosomal miRNAs released from CK+ cells (i.e., resembling a diseased state) and compared their expression with exosomal miRNAs from CK− cells (i.e., healthy, wild-type cells). Normalized ΔCt values of exosomal miRNAs analyzed by the miRNA panel were obtained for each sample group and the relative fold change (FC) was expressed as $2^{-\Delta\Delta Ct}$. A total of 30 exosomal miRNAs were differentially expressed ($p < 0.05$) in response to cytokine stimulation, when CK+ exosomes were compared with CK− exosomes (Figure 3A; Table S2). Of the 30 differentially dysregulated exosomal miRNAs, 26 exosomal miRNAs were upregulated (FC $\geq$ 1.6), whereas four exosomal miRNAs were found to be downregulated (FC $\leq$ 0.6) (Table S2). Interestingly, 12 of these differentially dysregulated exosomal miRNAs overlapped

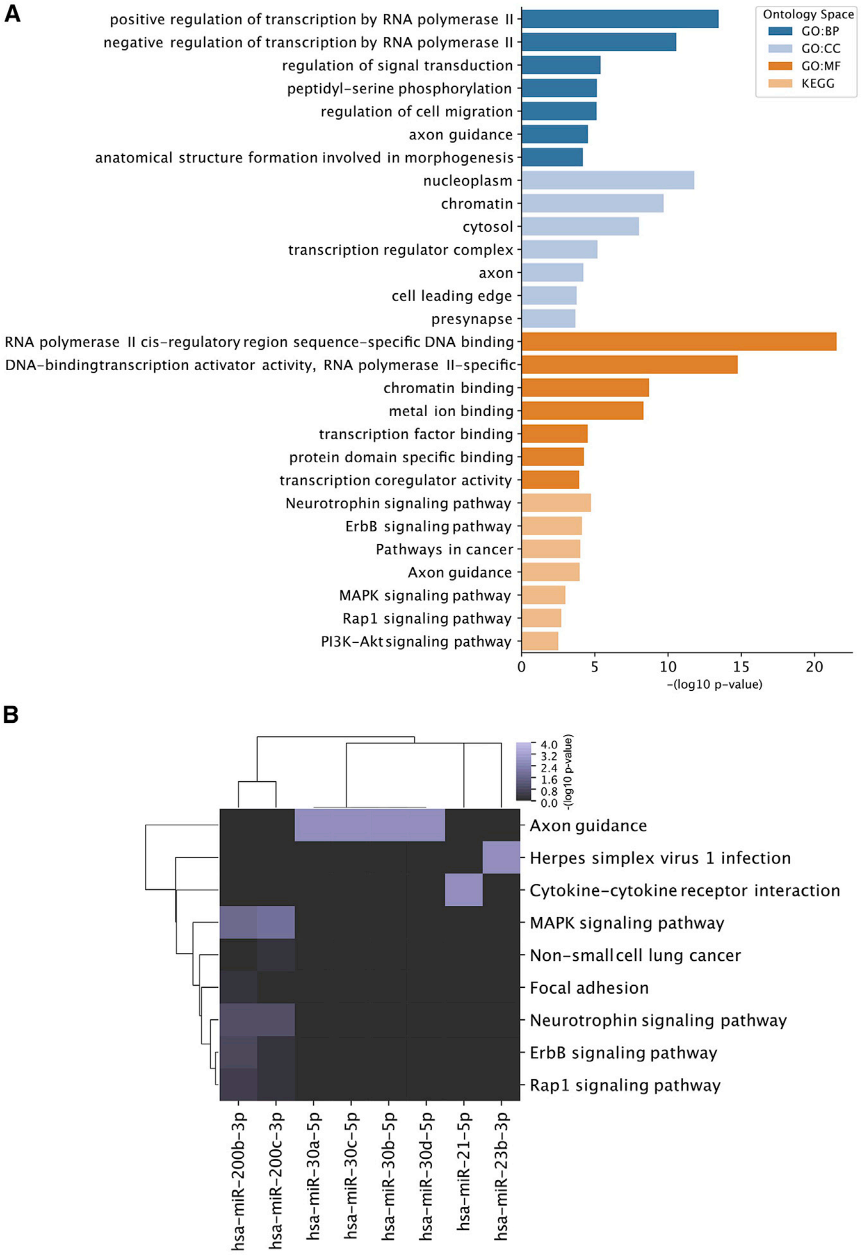

Figure 4. Pathway analysis of dysregulated and highly abundant exosomal miRNAs

(A) Gene Ontology analysis of the target space derived from 12 dysregulated and highly abundant exosomal miRNAs (hsa-miR-155-5p, -192-5p, -196a-5p, 200b/c-3p, -21-5p, 215-5p, 23b-3p, and -30a/b/c/d-5p) was performed for the ontology spaces (GO:BP, GO:CC, GO:MF, and KEGG) and the top seven enriched pathways are represented by the ranked negative $\log_{10}$ p value for each ontology spaces. (B) KEGG pathway analysis of the target space (retrieved from the DIANA microT-CDS prediction tool [score >0.9]) for each of the 12 most significantly dysregulated and highly abundant exosomal miRNAs from cytokine-stimulated RPTECs. Enriched pathways are represented and ranked by the negative $\log_{10}$ p value.

with the 20 most highly expressed miRNAs sorted into exosomes (Figures 3A and S5A).

The relative expression of selected exosomal miRNAs (hsa-miR-21-5p, -192-5p, -215-5p, -194-5p, -146a-5p, and -126-3p), which has been reported to be associated with kidney disease,[65–68] was validated and analyzed by an individual LNA miRNA PCR assay (Figure 3B). The expression level of exosomal miRNAs was analyzed by employing miR-191-5p and let-7b-5p as reference genes, and the relative abundance was expressed as FC ($FC = 2^{-\Delta\Delta Ct}$). Our results from the individual miRNA expression analysis of the six candidate exosomal miRNAs ($p < 0.05$) confirmed the PCR Panel-based miRNA profiling analysis.

Pathway analysis of dysregulated and highly abundant exosomal miRNAs

To identify putative pathways regulated by exosomal miRNAs, target space analysis of the 12 dysregulated and most abundant exosomal miRNAs (hsa-miR-155-5p, hsa-miR-192-5p, hsa-miR-196a-5p, hsa-miR-200b/c-3p, hsa-miR-21-5p, hsa-miR-215-5p, hsa-miR-23b-3p, hsa-miR-30a/b/c/d-5p) was performed using the DIANA microT-CDS v5.0 prediction algorithm. Applying a high confidence threshold (target score >0.9) revealed 2011 unique gene targets predicted to be suppressed by the queried miRNAs. Gene Ontology (GO) analysis was performed subsequently to reveal enriched GO pathways in four annotation spaces (biological process [GO:BP], cellular components [GO:CC], molecular function [GO:MF], and Kyoto Encyclopedia of Genes and Genomes [KEGG]). This revealed RNA polymerase-related pathways (positive regulation of transcription by RNA polymerase II [GO:BP], RNA polymerase II *cis*-regulatory region sequence-specific DNA binding [GO:MF]) as well as signaling pathways such as the mitogen-activated protein kinase (MAPK)-signaling pathway, Rap1-signaling pathway, and the phosphoinositide 3-kinase (PI3K)-AKT-signaling pathways as putative target pathways for released exosomal miRNAs on recipient cells (Figure 4A). To elucidate the role of each individual miRNA, KEGG pathway analysis was additionally performed on each single miRNA target space, which revealed that predominantly the miR-200 family is enriched for signaling pathway-related targets (ErbB signaling pathway, Rap1 signaling pathway, and neurotrophin signaling pathway [KEGG]), whereas miR-21 is predicted to suppress the

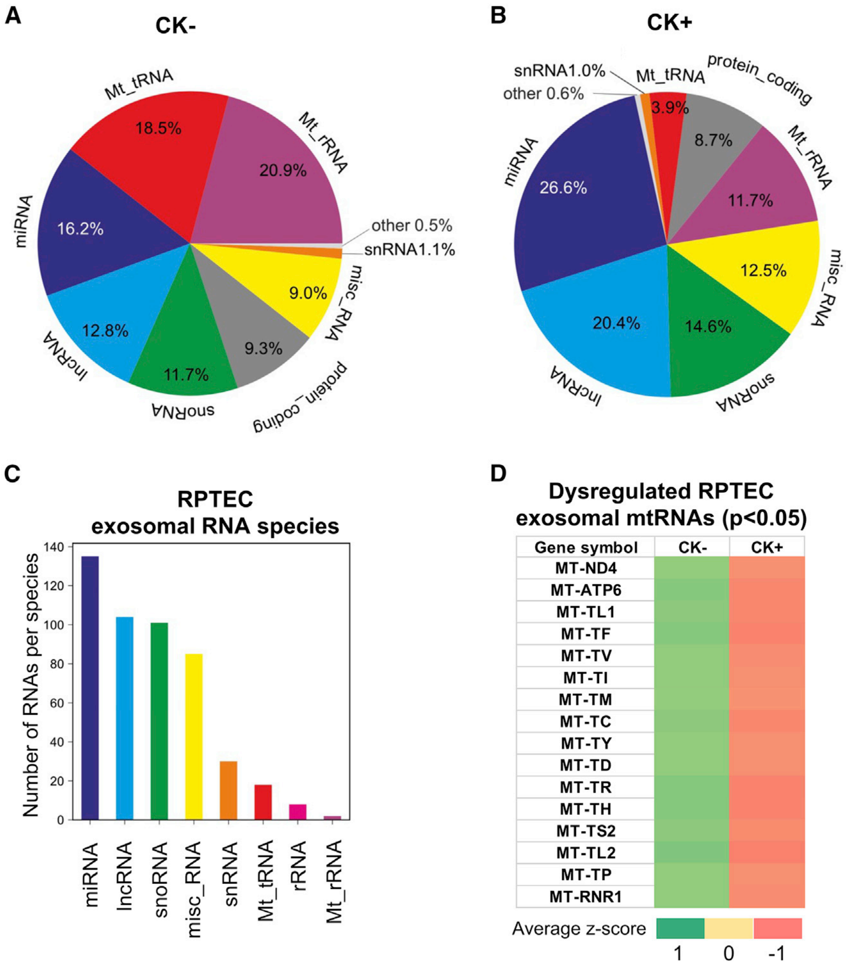

Figure 5. Identification of dysregulated exosomal ncRNAs from a cell-based model system of renal inflammation by RNA sequencing

Distribution of exosomal RNAs derived from (A) non-stimulated (CK−) and (B) cytokine-stimulated (CK+) RPTECs. (C) Total number of exosomal RNA per species obtained from CK− and CK+ RPTECs. (D) Heatmap of differentially expressed exosomal RNAs (adjusted p < 0.05) derived from CK− and CK+ RPTECs. Heatmap colors correspond to the average of normalized *Z* score as indicated in the color range. Red indicates low counts and green indicates high counts.

cytokine-cytokine receptor interaction pathway, thereby being putatively involved in immunomodulatory effects (Figure 4B).

Comparison of ncRNA abundance in exosomes derived from CK− versus CK+

In addition to miRNA profiling, we extended our investigations from miRNAs to longer ncRNAs by next-generation sequencing (RNA-seq) analysis in order to identify additional dysregulated exosomal ncRNAs associated with the pathophysiology of inflammatory kidney disease. By employing our *in vitro* RPTEC culture system, we analyzed the differential expression of exosome-derived small RNA species from RPTECs (i.e., CK+ or CK−, respectively) by RNA-seq analysis.

Initially, we performed principal component analysis (PCA) of RNA expression profiles between the CK+ group and CK− group and identified two distinct clusters, separating the diseased group (CK+) from the non-diseased group (CK−) (Figure S6A). Transcriptome analysis revealed that several ncRNA transcripts (i.e., lncRNAs, small nucleolar RNAs [snoRNAs], miscellaneous RNAs [misc_RNAs], small nuclear RNAs [snRNAs], mt-tRNAs, ribosomal RNAs [rRNAs]and mitochondrial-ribosomal RNAs [mt-rRNAs]) (Figures 5A–5C) were expressed in exosomes derived from RPTECs in addition to miRNAs. Importantly, the comparison between CK− and CK+ samples, showed an apparent difference in the normalized count distribution of specific biotype RNA species, in particular lncRNAs, miRNAs, mt-tRNAs, and mt-rRNAs (Figures 5A and 5B). Based on the number of normalized read counts, we identified that the percentages of the total RNA species of lncRNAs (12.8%) and miRNAs (16.2%) in the CK− sample were elevated in CK+ exosomes to 20.4% and 26.6%, respectively, while the distribution of exosomal mt-tRNAs (18.5%) and exosomal mt-rRNAs (20.9%) identified in CK− exosomes were found to be decreased in CK+ exosomes (3.9% and 11.7%, respectively) (Figures 5A and 5B).

Based on the normalized reads/counts, we further analyzed the 20 most abundant exosomal RNAs in CK− and CK+ samples (Table S3) and determined that the exosomal RNAs, such as lncRNAs (i.e., AC073140.2 and GAS5), miRNAs (i.e., miR-30a, miR-10b and miR-31), misc_RNAs (i.e., RNY1 and VTRNA1-1), mtRNAs (i.e., MT-RNR2, MT-RNR1, and MT-TM), and snoRNAs (i.e., SNORD6, SNORD69 and SNORD100), were the most abundant RNA species in both samples.

When we analyzed the differential abundance of these ncRNA species in CK− versus CK+ exosomes, our analysis revealed 16 significantly dysregulated exosomal RNAs (p < 0.05 adjusted), all of which mapped to the mitochondrial genome (Figures 5D and S6B; Table S4).

Interestingly, of these dysregulated exosomal mtRNAs, the majority (i.e., 81.25%) could be assigned to the class of mt-tRNAs. Based on the normalized number of reads, the expression of all significantly dysregulated exosomal mtRNAs in CK+ samples was downregulated relative to the CK− exosome samples.

In order to rule out the presence of contaminating mitochondria, we performed an immunoblot analysis of cytochrome C, which is a

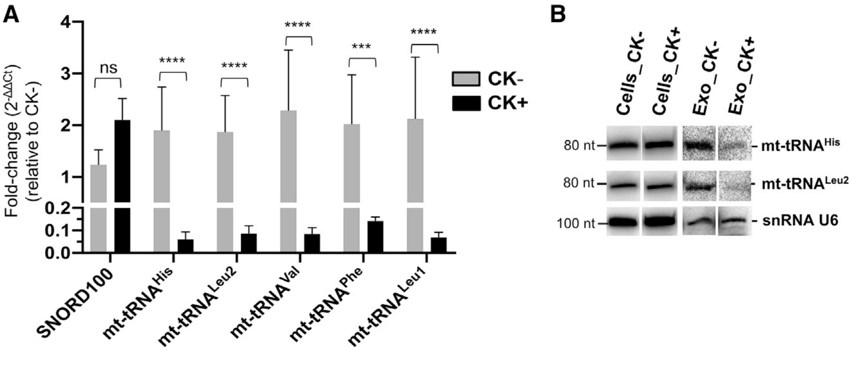

Figure 6. Validation of dysregulated exosomal mt-tRNAs, mt-tRNA^{His}, mt-tRNA^{Leu2}, mt-tRNA^{Val}, mt-tRNA^{Phe}, and mt-tRNA^{Leu1} derived from an RPTEC model system of renal inflammation

(A) RT-qPCR analysis of total exosomal RNA extracts from either non-stimulated (CK−) or cytokine-stimulated (CK+) RPTECs (see section "materials and methods"). The FC expression of each candidate, relative to CK− samples, was analyzed by the $2^{-\Delta\Delta Ct}$ method. Data indicate mean ± SEM of four independent experiments. Statistical analysis of ΔCt values was performed using two-way ANOVA with multiple comparison test and Bonferroni correction (GraphPad Prism 8.0.1). ****$p < 0.0001$; ***$p < 0.001$; and ns, not significant. (B) Northern blot analysis of mt-tRNA^{His} and mt-tRNA^{Leu2} (see section "materials and methods"). Total RNA extracts were derived from either RPTEC exosomes (Exo) or cells under non-stimulated (CK−) or cytokine-stimulated (CK+) condition.

reported and validated marker for mitochondrial organelles, and, concurrently, the exosome marker protein TSG101. These analyses revealed that cytochrome C was undetectable in both exosome preparations (CK− and CK+), while TSG101 was consistently present in both exosome samples, but with increased abundance in CK+ compared with CK− samples, as observed previously (Figure S7A). Hence, these results indicate that exosome preparations from RPTECs are unlikely to be contaminated with mitochondrial organelles or debris.

For the validation of our mt-tRNA results, we selected five candidates (i.e., mt-tRNA^{His}, mt-tRNA^{Leu2}, mt-tRNA^{Val}, mt-tRNA^{Phe}, and mt-tRNA^{Leu1}, respectively) with the highest abundance as deduced by RNA-seq analysis, and investigated their expression levels in CK+ exosomes versus CK− exosomes by RT-qPCR. The FC ($2^{-\Delta\Delta Ct}$) in expression of these candidates was obtained by normalizing to CK−, employing snRNA U6 and β-actin as reference genes.[69,70] These analyses showed that the relative FC of all candidate exosomal mt-tRNAs was consistently downregulated by 14- to 31-fold in CK+, relative to CK− exosomes (Figure 6A).

To verify the lower abundance of mt-tRNAs in exosomes derived from CK+ cells and to determine whether they are expressed as full-length tRNAs or tRNA fragments, we performed northern blot analysis of the total cellular and exosomal RNA extracts of CK+ and CK− samples. We selected two representative candidates (mt-tRNA^{His} and mt-tRNA^{Leu2}) and determined their abundance by northern blot analysis using equal amounts of total cellular or exosomal RNA. Ethidium bromide staining and the expression of snRNA U6 were used as loading controls (Figures S8A and 6B, lowest panel). Northern blot analysis showed that predominantly full-length mt-tRNAs of representative candidate mt-RNAs were found in CK+ or CK− cellular RNA extracts (Figure 6B). The abundance of two selected exosomal mt-tRNAs was found to be consistently decreased in exosomes derived from CK+ cells (Figure 6B), which was consistent with RNA-seq and RT-qPCR analysis (Figures 5D and 6A).

Abundance of selected candidate miRNAs and mt-tRNAs in exosomes versus their parental cells

To investigate the ratio of exosomal versus cellular miRNAs and mt-tRNAs, respectively, we first analyzed the abundance of three dysregulated exosomal miRNAs (hsa-miR-21-5p, -215-5p, and -192-5p) by qPCR and compared their abundance with that found within cells. We determined the copy number of candidate miRNAs in both exosomes and cells, and based on this we inferred the percentage that gets released into exosomes. From these analyses, the percentage release of candidate exosomal miRNAs hsa-miR-21-5p (0.4%), hsa-miR-192-5p (0.6%), and hsa-miR-215-5p (1.8%) in CK+ exosomes was significantly higher than in CK− exosomes (<0.1%) (Figure 7A). Comparison between CK− and CK+ showed that the percentage release of these selected candidate exosomal miRNAs was consistently upregulated in CK+ samples (24- to 28-fold), relative to CK− samples in response to cytokine stimulation (Figure 7A).

We then determined whether candidate exosomal miRNAs with enhanced expression in CK+ samples was correlated with the expression of the respective miRNA in parental cells. The cellular miRNA abundance or RNA copy numbers of each candidate miRNA across all samples were analyzed. Relative to the CK− sample (set to 1), the total RNA copy numbers of hsa-miR-21-5p (0.9-fold), hsa-miR-215-5p (0.6-fold), and hsa-miR-192-5p (0.8-fold) of CK+ parental cells were not considerably altered (Figures S9A–S9C). This suggests that cytokine stimulation does not significantly alter the abundance of these candidate miRNAs within the cells but rather can mediate the selective sorting and exosomal release of candidate miRNAs.

We employed a similar analysis to selected candidate exosomal mt-tRNAs and determined that the percentage release of exosomal mt-tRNA^{His} (0.016%), mt-tRNA^{Leu1} (0.011%), and mt-tRNA^{Val} (0.0013%) was notably reduced in CK+ samples by 4- to 6-fold, compared with that in CK− samples (0.081%, 0.04%, and 0.008%, respectively) (Figure 7B), confirming our initial finding employing the $2^{-\Delta\Delta Ct}$ method (Figure 6A). Similar to miRNAs, the abundance of candidate mt-tRNAs showed no sizable difference in parental cells

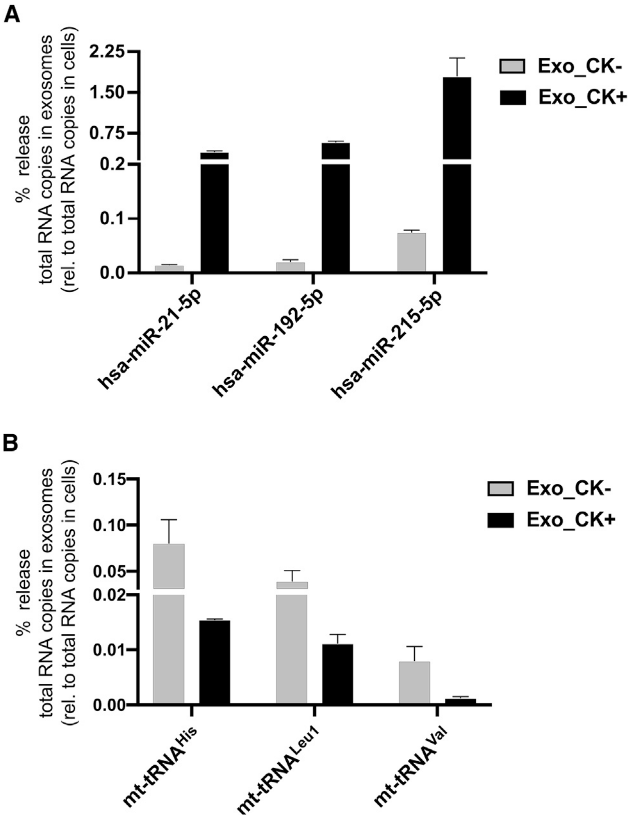

Figure 7. Cytokine-mediated release of candidate exosomal miRNAs and mt-tRNAs in RPTECs

(A) Absolute quantification PCR analysis of dysregulated exosomal miRNAs, hsa-miR-21-5p, -192-5p, and -215-5p, derived either from non-stimulated (CK−) or cytokine-stimulated (CK+) RPTECs. The percentage release of each candidate exosomal miRNA was calculated by obtaining the total RNA copies in exosomes and normalized to the total RNA copies in cells (set to 100%) (see section "materials and methods"). Data represent mean ± SD, which consists of three independent samples pooled together. (B) The percentage release of candidate exosomal mt-tRNAs, mt-tRNA^{His}, mt-tRNA^{Leu1}, and mt-tRNA^{Val} was analyzed similar to (A). Bar graph represents mean ± SEM of two independent experiments.

between CK− and CK+ (Figures S10A–S10C), suggesting that cytokine treatment does not affect the expression of the selected candidates in these cells, but strongly affects their sorting and release into exosomes. Furthermore, the abundance of all candidate exosomal miRNAs and mt-tRNAs, either in CK− or CK+, was found to be significantly lower compared with their abundance in parental cells.

Cytokine-mediated release of exosomal miRNAs and mt-tRNAs is altered by exosome inhibitors

In order to unequivocally demonstrate that candidate exosomal miRNAs (in particular hsa-miR-21-5p, -215-5p, and -192-5p, respectively) are selectively sorted into exosomes in response to cytokine treatment, we subjected the RPTEC *in vitro* culture system to a combination of inhibitors of exosome biogenesis and release (i.e., GW4869 and manumycin A).

We analyzed the abundance or percentage release of three dysregulated exosomal miRNAs (hsa-miR-21-5p, -215-5p, and -192-5p), in the presence or absence of GW4869 and/or manumycin A, by RT-qPCR relative to their abundance within the cells, based on the total RNA copy numbers in exosomes relative to the total RNA copy numbers in cells (Figures 8A–8C and S11A–S11C). Comparison of the percentage release of each candidate exosomal miRNA demonstrated that the percentage release of exosomal hsa-miR-21-5p and -192-5p was observed to be marginally reduced in CK+ following the administration of a single exosome inhibitor, compared with CK+ without inhibitor (Figures S11A and S11B), whereas the percentage release of exosomal hsa-miR-215-5p was strongly reduced in CK+ with manumycin A and marginally reduced in CK+ with GW4869 relative to the CK+ in the absence of inhibitor (Figure S11C). Strikingly, the percentage release of three candidate exosomal miRNAs (hsa-miR-21-5p, -192-5p, and -miR-215-5p) was also strongly reduced by 3- to 7-fold in the CK+ in the presence of two inhibitors, relative to their abundance in CK+ without inhibitors (Figures 8A–8C), suggesting that a combination of two inhibitors may have synergistic effect in inhibiting exosomal release of candidate miRNAs.

To determine whether the reduction of candidate exosomal miRNAs is correlated with the expression of the respective miRNA in parental cells, we also analyzed the cellular miRNA abundance or copy numbers of each candidate miRNA across all samples. Relative to the CK− sample, the addition of GW4869 and manumycin A had no pronounced effect on the abundance of cellular hsa-miR-21-5p and hsa-miR-192-5p in the CK+ sample, across all samples, except for hsa-miR-215-5p (Figures S9A–S9C). Our data therefore suggest that the abundance of these selected candidate miRNAs within the cells is not directly correlated with the extent of their exosomal release under these conditions.

We also investigated the effects of inhibitors GW4869 and manumycin A on regulating the release of selected candidate exosomal mt-tRNAs. Contrary to candidate exosomal miRNA release, the abundance of candidate exosomal mt-tRNAs, mt-tRNA^{Leu1} and mt-tRNA^{Val}, was found to be upregulated by 2- to 4-fold in CK+ samples upon addition of both inhibitors, compared with the untreated CK+ samples, except for mt-tRNA^{His} (Figures 8D–8F). An increase was observed for exosomal mt-tRNA^{Leu1} and mt-tRNA^{Val} in CK− samples in the presence of both inhibitors (Figures 8E and 8F), except for mt-tRNA^{His} (Figure 8D). In cells, the abundance of these candidate exosomal mt-tRNAs was also enhanced by 2-fold in both CK− and CK+ in the presence of two inhibitors, relative to the untreated samples (CK− and CK+, respectively) (Figures S10A–S10C).

DISCUSSION

RPTECs play a vital role in kidney function. In response to kidney injury, however, they are also able to promote progression of CKD via the synthesis of various bioactive molecules (e.g., cytokines/chemokines, RNAs, DNAs, or proteins) that drive interstitial inflammation and fibrosis.[55,71–73] The transfer of these bioactive molecules into the extracellular milieu can be facilitated via exosomes. For example,

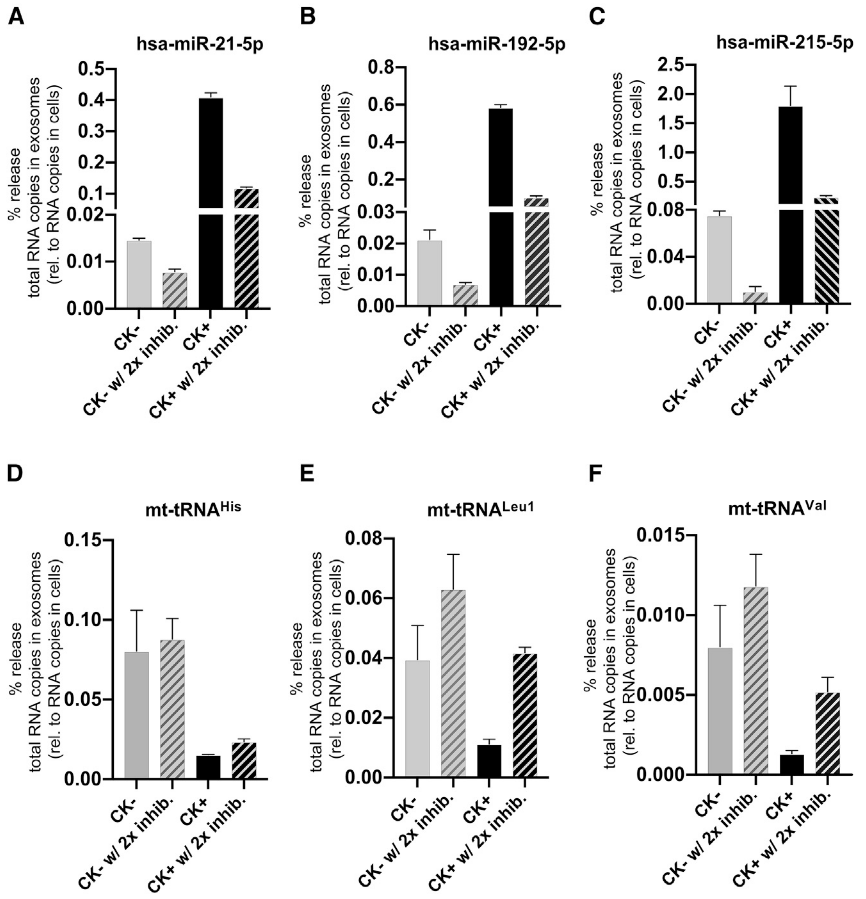

Figure 8. Regulation of candidate exosomal miRNAs and mt-tRNAs from RPTECs by combinatorial treatment of GW4869 and manumycin A

(A–C) RT-qPCR analysis of dysregulated exosomal miRNAs, hsa-miR-21-5p, -215-5p, and -192-5p, derived from non-stimulated (CK−) or cytokine-stimulated (CK+) RPTECs treated with both inhibitors (2× inhib.), GW4869 (5 μM), and manumycin A (125 nM). The percentage release of each candidate exosomal miRNA was calculated by obtaining the total RNA copies in exosomes and normalized to the total RNA copies in cells (set to 100%) for each treatment (see section "materials and methods"). Data represent mean ± SD, which consists of three independent samples pooled together. (D–F) The percentage release of each candidate exosomal mt-tRNAs, mt-tRNA^{His}, mt-tRNA^{Leu1}, and mt-tRNA^{Val} was analyzed similar to (A–C). Data represent mean ± SEM of two independent experiments.

injured tubular epithelial cells can release exosomes containing TGF-β mRNA, which can activate fibroblasts, contributing to the development of renal fibrosis in post-acute kidney injury kidneys.[74] Exosomes are EVs that play a role not only in cell-to-cell communication within nephron segments but also in various kidney disorders.[22,31,75] The molecular composition of exosomes reflects the physiological or pathological state of their cellular origin. Hence, exosomes might serve as potential liquid biomarkers.[32,59]

In kidney-related diseases, an altered expression of urinary exosomal miRNAs has been found to correlate with the progression of kidney disease.[19,36] Thus, urinary exosome analysis is currently employed to correlate expression of biomarkers, such as miRNAs, to specific disease states (e.g., CKD).[19,75] In our previous study, we identified several urinary exosomal biomarkers from CKD patients and from an HC group through RNA-seq analysis,[19] but the potential cellular source of these molecules remained elusive.

To demonstrate that urinary exosomal ncRNAs are mainly derived from kidney-related cell types, in particular RPTECs, we evaluated urinary exosomal miRNA profiles of an HC group and compared them with that of non-stimulated/control RPTECs. By these analyses, we showed that a large fraction of urinary exosomal miRNAs were likely to be directly derived from RPTECs. Comparison between the HC group versus non-stimulated RPTECs showed that about 50% of the 20 most abundant exosomal miRNAs (Figure 1C) were shared between the two groups. Based on this assessment, we suggest that RPTECs are a suitable model system for the identification of exosomal biomarkers that are associated with CKD pathophysiology.

Interestingly, our findings also reflect the fact that urinary exosomes may be derived from other sources as well, as depicted by the non-overlapping exosomal miRNAs profiles between the two groups. Based on cell-specific markers, urinary exosomes are derived from other parts of the kidney (e.g., nephron segments) or non-kidney-related tissue (e.g., bladder and prostate).[76,77] Individual metabolic conditions influenced by several factors (e.g., type of diet, exercise, or stress) might also contribute to the heterogeneous population of urinary exosomes and other EVs, and, consequently, to a shift in their specific cargoes.[59,78]

We aimed to gain insights into the molecular changes that reflect CKD and/or that drive the progression of CKD by employing the RPTEC culture model system that resembles some aspects of CKD. Therefore, we transformed RPTECs into a pro-inflammatory and pro-fibrotic disease state (designated as CK+) by stimulating the cells with cytokines (IL-1β, OSM, and TGF-β1) that are implicated in the development of phenotypes also observed in CKD (e.g., inflammation and fibrosis).[79,80]

The cytokine-induced pro-fibrotic state of RPTECs, which were immortalized by TERT1, might be influenced by the increased telomerase activity in these cells. TGF-β is a key regulator in the development of fibrotic diseases and the activation of the canonical Wnt signaling. This is achieved in a p38-dependent manner by decreasing

the expression of the Wnt antagonist Dickkopf-1, which is essential for TGF-β-mediated fibrosis.[81] It has previously been shown that overexpression of TERT can promote Wnt signaling,[82] leading to an increase in cellular response (e.g., cellular dedifferentiation[83]), which is a hallmark of fibrosis.[7] However, it has also been demonstrated that the effect of TERT overexpression on Wnt signaling was not evident in different human breast cancer cell lines, suggesting that TERT-induced Wnt signaling is cell-type specific and that, in certain contexts, telomerase activity may not necessarily stimulate Wnt signaling.[84] It is also quite unlikely that telomerase activity has an influence on the dedifferentiation of RPTECs, since comparable effects of TGF-β1 and/or OSM on phosphorylation of downstream mediators Smad2/3 and Smad1/5/8 were observed in both RPTEC/TERT1 cells and HK-2 cells, despite the latter being immortalized independently of TERT via overexpression of human papilloma E6/E7 proteins.[13] This indicates that the enhanced telomerase activity may not necessarily affect the transformation of RPTECs into their pro-fibrotic state upon cytokine stimulation.

In our model system, we found that pro-inflammatory and pro-fibrotic cytokines stimulated the release of different populations of exosomes, as shown by increased levels of the exosomal markers TSG101 and CD9, and larger clusters of exosomes, compared with the non-stimulated condition. An induced release of EVs, in particular exosomes, might be a common cellular response to inflammation.[85,86] Accordingly, in a recent study it was also observed that the release of EVs is significantly increased during inflammation, which might be linked to the pathogenesis in neuroinflammatory diseases.[87] In damaged kidney cells, an increased secretion of EVs and their cargo promotes inflammation and fibrosis.[86] Since EVs are generally accepted as significant contributors to inflammation and pathogenesis,[87] the analysis of exosomes in an *in vitro* model system for CKD is justified, as they are likely to be altered and may recapitulate aspects of kidney disease pathophysiology.

Previous studies revealed that exosomal pathways are essential in cellular processes (e.g., reticulocyte maturation or membrane remodeling and senescence-like cell-cycle arrest or apoptosis in human cells) as a means to eliminate proteins or harmful DNA materials.[88–91] It is therefore conceivable that the export of specific miRNAs, such as miR-21, via the exosomal pathway might be employed to eliminate miRNAs associated with kidney disease from cells. In contrast, it has also been shown that an uptake of exosome cargo (e.g., RNAs) by other cells, either in the vicinity of or at distant sites from the releasing cells, can modulate phenotypic responses.[59] Exosomes and other EVs can facilitate cell-to-cell communication, mediating protective or injury signals.[92] In kidney cells, signaling between nephron regions can be mediated by miRNAs derived from EVs (i.e., exosomes).[93] In kidney injury, some miRNAs (e.g., miR-21, miR-126, miR-146a, and miR-200 family) have protective effects (e.g., anti-apoptosis, anti-inflammation, and anti-fibrosis), while some miRNAs (e.g., miR-21 and miR-192) elicit pathological influence by promoting apoptosis, inflammation, and fibrosis.[94] Thus, we speculate that the release of highly abundant exosomal miRNAs, which we observed to be common to both the non-stimulated and stimulated conditions, might be essential for maintenance of cellular homeostasis in normal proximal tubule cell type and/or recipient cells, and that their altered abundance in response to cytokine stimulation might promote inflammation and the development of CKD.

The primary part of the nephron (i.e., proximal tubule and glomerulus) is a known source of exosomes and other EVs found in urine.[76,95,96] This is supported by our data demonstrating that the high prevalence of highly abundant RPTEC-derived exosomal miRNAs was mirrored in the urine samples (e.g., unstimulated condition versus healthy group). As such, our findings suggest that the significantly altered abundance of exosomal miRNAs (Figure 3A) potentially reflect a diseased cell type (i.e., proximal tubule cell type) in response to inflammation and fibrosis. We also found that the highly abundant and significantly dysregulated exosomal miRNAs (i.e., hsa-miR-192-5p, -21-5p, -215-5p, −200b-3p, and −200c-3p) were upregulated in cytokine-induced exosomes, an observation that is consistent with *in vivo* studies of acute kidney injury and diabetic nephropathy, the leading cause of CKD.[93,94,97] Interestingly, by comparing differentially expressed exosomal miRNAs from cytokine-stimulated RPTECs with urinary exosomal miRNAs from clinical CKD patient samples and controls ($N = 25$), as previously reported by our group,[19] we found that eight out of 30 exosomal miRNA species differentially expressed in cytokine-stimulated RPTECs were also differentially expressed in the urinary exosomes derived from different CKD stages (Table S5). The dysregulated exosomal miRNAs (i.e., hsa-miR-215-5p, hsa-miR-192-5p, hsa-miR-146a-5p, hsa-miR-23b-3p, hsa-miR-126-5p, hsa-miR-126-3p, hsa-miR-21-5p, and hsa-miR-31-3p) mostly represent an early stage of CKD, i.e., stage II, followed by stage IV, stage III, and then stage I (Table S5).

Our findings thus suggest that renal tubular cells of the kidney might be a direct source of signaling vesicles. The identification of highly abundant and/or dysregulated exosomal miRNAs upon cytokine stimulation (Figures 1B and 3A) can be a valuable undertaking since it may unveil potential physiological and pathological processes related to CKD.

In addition, prediction of miRNA target spaces might help to elucidate the putative signaling roles of exosomal miRNAs differentially expressed from cytokine-stimulated RPTECs, since these miRNAs might be associated with the pathogenesis of CKD. Interestingly, differentially expressed exosomal miRNAs are implicated in the regulation of signaling pathways such as ErbB signaling or PI3K-AKT signaling, which are linked with the regulation of immune responses (e.g., inflammation).[98,99] This might suggest that exosomal miRNAs can act as "secondary messenger" molecules, which signal the inflammatory state of the kidney to recipient cells. In addition, KEGG pathway enrichments of miR-21 target spaces suggest direct roles in the suppression of cytokine receptor interactions.

We also investigated other RNA signatures that specifically originate from RPTECs and might be involved in modulating CKD

phenotypes. Based on RNA-seq analysis, we revealed that dysregulated exosomal RNAs (Figure 5D; Table S4) originated from the mitochondria, predominantly mt-tRNAs, implying a role for the mitochondria in sensing inflammation and fibrosis. Previous studies have shown that mtRNAs are secreted into exosomes,[100–102] which suggests that exosomal mtRNAs may be engaged in late endosome formation involving mitophagy and exosome synthesis or fusion of mitochondria with lysosomes, which facilitates transfer of mitochondria content into vesicles.[103–105] Among renal cell types, proximal tubular cells contain more mitochondria than any other structure in the kidney and are susceptible to mitochondrial dysfunction, which can lead to apoptosis or necrosis of RPTECs[106] and is associated with the pathogenesis of CKD.[107–109] Mitochondria also play a vital role in autophagy.[107,110] These mitochondria-dependent pathways can be induced by cytokines (i.e., TGF-β, IL-1β, and OSM), promoting activation of caspase-3, accumulation of autophagosomes and LC3-II, and release of cytochrome C.[111–115]

Consistent with these observations, we detected elevated levels of cleaved caspase-3 and LC3B, markers for apoptosis and autophagy respectively, in cytokine-treated RPTECs, relative to non-stimulated RPTECs (Figures S7B and S7C). We thus envision that the impaired sorting, secretion, and release of mtRNAs via exosomes in cytokine-stimulated cells might be attributable to TGF-β1/IL-1β/OSM-mediated induction of apoptosis and/or activation of autophagy. In contrast to impaired exosomal mtRNA release in cytokine-stimulated cells, an enhanced exosomal release of mtRNAs in non-stimulated cells might reflect an important cellular process in maintaining cellular homeostasis and repair in the kidney.[116,117]

From a total of 16 dysregulated exosomal mtRNAs, we identified 13 mt-tRNAs (i.e., mt-tRNA^{Cys}, mt-tRNA^{Ile}, mt-tRNA^{Leu2}, mt-tRNA^{Ser2}, mt-tRNA^{Leu1}, mt-tRNA^{His}, mt-tRNA^{Met}, mt-tRNA^{Val}, mt-tRNA^{Pro}, mt-tRNA^{Tyr}, mt-tRNA^{Arg}, mt-tRNA^{Phe}, and mt-tRNA^{Asp}) that were also found to be differentially abundant in previous next-generation sequencing analysis of differentially expressed ncRNAs in urinary exosomes derived from CKD patients and controls (N = 25) at different CKD stages (i.e., stages I–IV, respectively; Table S6).[19] As for CK+ RPTECs, these exosomal mt-tRNAs were found to be consistently downregulated in different CKD stages, and the majority of the dysregulated exosomal mtRNA candidates were present in stage I, followed by stage III, stage II, and then stage IV.

Although our findings concerning differentially abundant exosomal miRNAs and mtRNAs still have to be extended to a larger patient cohort, they further support our hypothesis that RPTECs might serve as major sources of urinary exosomes and, in addition, that these two classes of ncRNAs might represent potential biomarkers for renal tubular inflammation-related CKD.

To gain insight into the expression and exosomal release of miRNAs and mt-tRNAs, which are dysregulated upon cytokine stimulation, we analyzed the abundance or total RNA copy number of selected candidate miRNAs and mt-tRNAs in exosomes versus their parental cells. Based on the RNA copy numbers, we demonstrated that only a very small fraction of miRNAs were present in exosomes compared with the cells (i.e., RPTECs) from which they were derived. It therefore seems unlikely that, at least in this RPTEC system, exosomes are employed to effectively eliminate specific mRNAs from cells, but rather they may provide a "snapshot" of the cytoplasm reflecting the physiological state of their parental cells.

We also demonstrated that cytokine-mediated release of candidate exosomal miRNAs and mtRNAs was regulated by known inhibitors of exosome biogenesis and release, GW4869 and manumycin A. Combined treatment with GW4869 and manumycin impeded the secretion of selected exosomal miRNAs, indicating that they are selectively sorted into exosomes in response to cytokine stimulation in RPTECs. The sorting and release of selected candidate exosomal miRNAs, as well as their exosome carriers, is likely mediated by the activation of Ras and neural sphingomyelinase 2 (nSMase2), which are involved in ESCRT-dependent and -independent exosomal pathways, respectively.[25,29,52] In contrast to candidate exosomal miRNAs, addition of both inhibitors did not prevent, and potentially even enhanced, sorting of candidate mt-tRNAs into exosomes under non-stimulated and stimulated conditions. This observation might be attributed to the different subpopulations of exosomes or EVs, preferentially harboring mt-tRNAs in addition to other ncRNAs. GW4869 not only inhibits biogenesis and release of exosomes but may also induce secretion and budding of other CD9-positive EVs/exosomes, altering the population of EVs/exosomes that harbor cargo molecules.[118] This is consistent with our observation of altered CD9 levels in both non-stimulated and stimulated conditions and might also reflect an enhanced cellular expression of candidate mt-tRNAs in cells upon addition of GW4869 and manumycin A. The downregulation of candidate exosomal mt-tRNAs expression was partially rescued by GW4869 and manumycin A, suggesting that these nSMAse and transfarnesyl inhibitors, respectively, may be able to restore mitochondria function.[27] Thus, targeting the exosomal pathways may lead to novel therapeutic avenues, since exosomes and their cargo molecules (e.g., RNAs, DNAs, and proteins) can modulate intercellular communication and are also potential indicators of diseased state (i.e., CKD).[19,30,32,75,119]

Through our analyses, we identified differentially abundant exosomal miRNA and mtRNA candidates in response to cytokine stimulation of RPTECs as being major contributors to the development and progression of inflammatory kidney disease leading to CKD.[14] Since these candidates can be regulated by inhibitors of exosome biogenesis and release, they can potentially be used for diagnostic purposes and for monitoring the efficacy of treatment of CKD associated with renal inflammation specifically originating from diseased RPTECs.

MATERIALS AND METHODS

Reagents and antibodies

Cell culture reagents were purchased from Gibco (Life Technologies) unless otherwise indicated. Other materials and reagents were

obtained as follows: IL-1β (R&D Systems); TGF-β1 (PeproTech); mouse anti-CD9 (Alexa 647 nm) (Bio-Rad), mouse anti-TSG101, mouse anti-caspase-3 and mouse anti-cytochrome c (Santa Cruz); rabbit anti-LC3B (Cell Signaling); mouse monoclonal anti-GAPDH (Proteintech); goat anti-mouse immunoglobulin G (IgG) (Millipore); goat anti-rabbit IgG (Thermo Fisher Scientific); primer sequences (Integrated DNA Technologies). All other reagents were obtained from Sigma.

RPTEC cell culture and exosome isolation

Human renal proximal tubular epithelial cell line (RPTEC/hTERT1) was used as a model system for renal inflammation and fibrosis. Cells were cultured as described previously.[47] Briefly, cells were grown in a serum-free mixture of DMEM/F-12 (1:1) medium containing insulin (5 μg/mL), transferrin (5 μg/mL), selenium (5 ng/mL) (ITS); GlutaMAX (2 mM); epithelial growth factor (EGF) (10 ng/mL); hydrocortisone (36 ng/mL); and penicillin (100 U/mL)/streptomycin (100 μL/mL) at 37°C in a humidified 5% CO_2 atmosphere. Cells were fed every 2–3 days until 95% confluency and grown for another 10 days to allow the cells to undergo differentiation. Subsequently, cells were made quiescent by incubation in serum-free medium containing 0.1% complete medium and penicillin/streptomycin for 48 h. Following starvation, cells were either left unstimulated (CK−) or stimulated (CK+) with TGF-β1 (10 ng/mL), IL-1β (10 ng/mL), and OSM (10 ng/mL) for another 48 h. Isolation of exosomes was performed as previously described with few modifications.[120] Culture media were harvested and sequentially pre-centrifuged at 2,500 × *g* for 20 min and 10,000 × *g* for another 10 min at 4°C to remove cell debris. Supernatants were transferred into Beckmann tubes and centrifuged at 100,000 × *g* for 1 h and 30 min at 4°C to obtain exosome pellets. Pellets were washed with cold 1× phosphate-buffered saline (PBS) and centrifuged in similar conditions. Samples were resuspended in 50–100 μL of nuclease-free water and stored at −80°C.

Exosome isolation from urine

Urine samples were derived from the CKD biobank project from the Department of Internal Medicine IV (Nephrology and Hypertension). The collection of blood and urine samples for biomarker research was approved by the Institutional Review Board of the Medical University Innsbruck (AN4492, February 28, 2014), and each patient signed an informed consent.

Exosomes were isolated as previously described[19,120] with few modifications. Briefly, urine samples were collected from six HCs. Samples were pre-centrifuged at 2500 × *g* for 20 min at 4°C. The supernatants were transferred into Beckmann tubes and centrifuged at 100,000 × *g* for 1 h and 30 min at 4°C. The pellets were resuspended in 1 mL of isolation solution (250 mM sucrose, 10 mM triethanolamine [pH 7.6]) and, subsequently, 50 μL 1 M dithiothreitol (DTT) was added to remove Tamm-Horsfall protein. Samples were vortexed, resuspended in 1× PBS, and centrifuged at 100,000 × *g* for another 1 h and 30 min at 4°C. The supernatants were carefully discarded, and the pellets were resuspended in 50–100 μL of nuclease-free water and stored at −80°C.

Immunofluorescence staining

Exosome samples were prepared from an equal volume of culture medium. Exosome pellets were resuspended in 1× PBS, blocked with 1% BSA in 1× PBS for 20 min and, subsequently, incubated with Alexa (647 nm)-labeled anti-CD9 (0.5 μg/mL) antibodies for 1 h at room temperature (RT). Following antibody incubation, samples were washed with 1× PBS and centrifuged at 100,000 × *g* for 1 h and 30 min at 4°C. For microplate fluorescence assay, anti-CD9 (Alexa 647 nm)-labeled exosomes were resuspended in 100 μL of 1× PBS, transferred into 96-well glass-bottom microplate, and analyzed on a microplate reader (CLARIOstar, BMG Labtech, Germany).

Microscopy analysis

STORM

Anti-CD9-labeled (Alexa 647 nm) exosomes were resuspended in 2% paraformaldehyde, incubated for 15 min, washed with 1× PBS, and centrifuged at 100,000 × *g* for another 1 h and 30 min at 4°C. The supernatants were carefully discarded, and the exosome pellets were resuspended in 1× PBS. Samples were transferred into polylysine-coated glass-bottom chamber slides and incubated in the dark at 4°C overnight. The analysis of CD9-labeled (Alexa 647 nm) exosomes was performed by employing the 2D STORM technique using a buffer consisting of 50 mM β-mercaptoethylamine hydrochloride (MEA), 5% (v/v) OxyFluor, 20% (v/v) of sodium DL-lactate solution, and 1× PBS (pH 8–8.5) (OxEA buffer) as described in Nahidiazar et al.[49] The Alexa 647 nm dye blinking was analyzed using an iMIC Digital Microscope (TILL Photonics, Germany) with Hamamatsu ORCA Flash 4 camera (FEI Munich Germany). A total of 10,000 raw frames were obtained and the data were processed using ImageJ/ThunderSTORM employing a built-in drift correction.[121] A background subtraction was employed and different size spot analysis for the quantification of exosomes was performed using IMARIS software (version 9.7.2). Photos are representative images. Scale bar, 1,000 nm.

TEM

Exosome pellets were obtained from an equal volume of culture medium. CK− samples were undiluted while CK+ samples were diluted with 1× PBS (1:10). An aliquot of 7 μL of exosomes in 1× PBS was absorbed onto a formvar carbon-coated copper grid for 8 min at RT. Subsequently, the excess liquid was removed, and the grids were stained with 0.5% uranyl acetate for 3 min[39] Finally, the excess stain was removed and the grids were air-dried for 3–5 min. Samples were viewed with a CM120 TEM at 80 kV (from Philips, Eindhoven, the Netherlands), equipped with a Morada digital camera (from EMSIS, Münster, Germany). Exosome diameters were measured on digital images with iTEM software (from EMSIS).

SDS-PAGE and western blotting

RPTEC exosome pellets and cells were lysed in lysis buffer (300 mM NaCl, 50 mM Tris pH 7.4, 1% Triton X-100, and proteases inhibitors)

and incubated on ice for 15 min. Following centrifugation of the samples at maximum speed, the total exosome or cell lysates were transferred into a new Eppendorf tube and an equal volume of 2× Laemmli sample buffer (4% sodium dodecylsulfate [SDS], 10% β-mercaptoethanol, 20% glycerol, 0.1 M Tris pH 6.8, and 0.005% of bromophenol blue) was added. Samples were denatured at 95°C for 5 min and incubated on ice. An equal volume of exosome lysates or an equal protein concentration of cell lysates was used for SDS polyacrylamide gel electrophoresis (SDS-PAGE). Proteins were resolved onto 10%–12% SDS gel, after which they were transferred onto polyvinylidene fluoride (PVDF) membranes by western blotting. Following protein transfer, membranes were blocked with 5% BSA in 1× PBS for 1 h at RT, incubated with the primary antibody (in 1× PBS/3%BSA/0.1% Tween 20) overnight at 4°C and washed three times with 1× PBS/0.1% Tween 20. Subsequently, membranes were incubated with the HRP-conjugated secondary antibody for 1 h at RT and washed three times with washing buffer. Protein bands were analyzed by immunoblotting and detected by the enhanced chemiluminescence (ECL) method.

Total RNA extraction

RPTECs were washed three times with cold 1× PBS, lysed with TRI reagent, according to manufacturer's instructions, and incubated for 5 min at RT. Chloroform solution was added to the samples and incubated for another 5 min at RT. The samples were centrifuged at 13,000 rpm for 15 min at 4°C, and the aqueous phase was carefully removed and transferred into a fresh tube. A second chloroform extraction was carried out to remove residual phenol. The aqueous phase was transferred into a new tube and isopropanol and glycol blue solution were added. Samples were vortexed for 15 s and incubated for 10 min at RT. The RNA pellets were obtained by centrifugation at 13,000 × *g* for 30 min at 4°C, washed with 800 μL of 80% ethanol, and subsequently centrifuged at 7500 × *g* for 5 min at 4°C. The pellets were air dried for 3–5 min at RT and dissolved in nuclease-free water. For RPTEC exosome pellets, total RNA extraction was carried out by employing miRNeasy Micro kit (Qiagen) in accordance with its instruction manual. The quality of total RNA extracts was analyzed by NanoDrop (ND-1000, Avantor, VWR) and Bioanalyzer (Agilent) using small RNA and RNA pico chips.

Reverse transcription and qPCR analysis

For miRNA expression profiling, we employed miRCURY LNA miRNA miRNome Human PCR Panel I (Qiagen) containing 368 miRNAs. Reverse transcription and qPCR analyses were performed using miRCURY LNA RT kit (Qiagen) and miRCURY LNA SYBR Green PCR kit (Qiagen), respectively, and according to the product's instructions with a few modifications. Briefly, an equal volume of total RNA extract derived from RPTEC exosomes, which were isolated from an equal volume of culture medium for each sample (i.e., CK− and CK+) (n = 3), were used for reverse transcription. For RPTECs, a fixed amount of 10 ng for each sample was used for reverse transcription. The generation of cDNA templates was carried out with the following conditions: 60-min incubation at 42°C; 5-min inactivation of reverse transcriptase at 95°C; and cooling at 4°C. The samples were diluted in 40 folds and dispensed on a 384-well open plate containing specific LNA miRNA primer set. The cDNA amplification was performed using a ViiA 7 real-time PCR instrument (Applied Biosystems) with the following PCR cycling conditions: 2 min at 95°C for initial heat activation; 10 s at 95°C for denaturation; and 60 s at 56°C for combined annealing and extension. The miRNA profiling was performed from three independent experiments. A similar analysis was performed for the urinary exosomal miRNA profiling in HC (n = 6), using a miRCURY LNA miRNA miRNome screen panel and kits as described above. For technical validation of miRNA candidates, 3 μL of total RNA extract derived from an equal volume of each sample were transcribed using miRCURY LNA individual miRNA PCR Assay (Qiagen) (Table S7), based on manufacturer's instructions. For mt-tRNA validation, miScript II RT kit (Qiagen) and Luna universal qPCR master mix (New England Biolabs) were employed, using specific forward primer for each candidate and a universal reverse primer (Table S7). Similar sample volume of tRNA was used and analyzed based on manufacturer's instructions with a few modifications. Briefly, reverse transcription was performed with the following conditions: 60-min incubation at 37°C; 5-min inactivation of reverse transcriptase at 95°C; and cooling at 4°C. Reverse-transcribed products were diluted in 20 folds. Subsequently, qPCR was performed with the following conditions: 1 min at 95°C for initial heat activation; 15 s at 95°C for denaturation; 30 s at 60°C for combined annealing and extension; and 40 cycles. Synthetic cDNAs were used as a standard for absolute quantification of miRNA copies, whereas a DNA template of mt-tRNAs containing a T7 promoter region was generated (Table S7) and transcribed using HiScribe T7 High Yield RNA Synthesis Kit (New England Biolabs). The RNA product was DNAse treated and purified using the phenol/chloroform/isoamyl (PCI) extraction and ethanol precipitation method. All samples were analyzed in triplicate. The list of primers and template sequences are presented in Table S7. The miRNA profiles of total RNA from RPTECs (i.e., CK+) and exosomes (i.e., CK− or CK+), and urine samples generated from the human PCR Panel I were determined. Ct values that were undetermined or >38 and incomplete data points were filtered out. For the urine samples, similar conditions were adapted, employing six biological replicates (n = 6) of HC samples. Data were normalized using the average of six reference genes namely, hsa-let-7a-5p, hsa-let-7b-5p, miR-191-5p, miR-26a-5p, miR-92a-3p, and miR-103a-3p.[41–45] The high expression and stability of these miRNAs in all samples were used as a basis for their adequacy as normalizers. The ΔCt values ($\Delta Ct_{(test)} = Ct_{\ (test)} - Ct_{\ (average\ of\ 6\times\ reference\ genes)}$) were obtained for each replicate in every group (CK− or CK+). The relative FC expression of miRNAs was expressed as $2^{-\Delta\Delta Ct}$, wherein $2^{-\Delta\Delta Ct} = \Delta Ct_{(CK+)} - \Delta Ct_{(CK-)}$.[122] To verify the significantly differentially expressed miRNAs ($p < 0.05$) derived from the human PCR Panel I analysis, hsa-miR-191-5p and hsa-let-7b-5p were selected as normalizers for the technical validation. The ΔCt values obtained from the human PCR Panel I were analyzed using Student's t test. For the validation of mtRNA candidates, the relative gene expression of candidate mt-tRNAs was analyzed using snRNA U6 and β-actin as reference genes. GraphPad Prism version 8 was employed using analysis of variance (ANOVA) with multiple comparison and Bonferroni

correction or as otherwise indicated to validate the significant differences in expression levels of miRNA or mt-tRNA candidates ($p < 0.05$) between CK− and CK+ in RPTEC exosomes.

Northern blot analysis

Total RNA extracts from RPTEC cells or exosomes were size fractionated using denaturing PAGE. An equal amount of total exosomal RNA (2.4 μg) and total cellular RNA (10 μg) for each condition (i.e., CK− or CK+) was used for PAGE. Each sample was obtained from six plates (pooled). Samples were heat denatured for 3 min at 95°C in RNA loading dye (NEB), cooled on ice for 10 min, and loaded on an 8% polyacrylamide gel containing 7 M urea. RNA bands were stained with ethidium bromide, visualized by a Transilluminator (Bio-Rad), and transferred onto a Hybond N^+ nylon membrane (GE Healthcare). RNA-membrane crosslinking was performed at 120 mJ by an ultraviolet (UV) crosslinker. The oligonucleotide probes complementary to mt-$tRNA^{His}$ and mt-$tRNA^{Leu2}$ were radioactively labeled with [γ-^{32}P]-ATP (Hartmann Analytics) using T4 polynucleotide kinase (NEB) and according to manufacturer's instructions. The probes were designed based on a consensus consequence identified in the RNA-seq analysis (Table S7). Following pre-hybridization of the membrane, the probe was added for hybridization at 42°C overnight. Subsequently, the membrane was washed three times with saline sodium citrate (SSC) (20× SSC: 3 M NaCl and 0.3 M sodium citrate, pH 7.0) buffer using different stringency (2× SSC, twice and 1× SSC, once) for 5 min each at RT. The radioactive signal was analyzed using a Typhoon PhosphorImager (GE Healthcare) and the images were processed using ImageJ software.

RNA-seq analysis

Next-generation sequencing-based RNA-seq analysis of total RNA extracts derived from RPTEC exosomes was performed by employing Ion Proton System (Ion Torrent, Life Technologies). Small RNA libraries, two for each group (i.e., CK− or CK+), were prepared using the Ion Total RNA-Seq Kit v2 (Ion Torrent, Life Technologies), which has the advantage of inhibiting cDNA synthesis of the adaptor byproduct, thus allowing cDNA separation with magnetic bead-based technology. A total amount of 20 ng of total RNA for each sample was used as a starting material for the cDNA library preparation. Reverse transcription was carried out as described in the kit manufacturer's instructions. The samples were pooled and loaded unto the Ion chip Kit v2 and sequenced on the Ion Proton sequencer (Ion Torrent, Life Technologies). Raw reads were collected and processed using the Torrent Suite software. The 3′ adaptors were trimmed and low-quality reads were filtered out, generating a FASTQ file for each sample.[123]

Pre-processing, mapping, and assembly

The quality of .fastq files was assessed using FastP and quality trimming as well as filtering performed with default parameters. Derived filtered mRNA.fastq files were aligned to the human reference genome (GRCh38.p13, release 33) provided by GENCODE using STAR (v2.7) software with the default parameter.[124] Raw RNA counts were obtained using HTseq with the stranded parameter set to "yes". Raw mRNA counts were determined and mapped using the GENCODE comprehensive gene annotation file GRCh38.p13_chr_patch_hapl_scaf.annotations.gtf.[124] Detection of differentially expressed genes and miRNAs was performed using the Bioconductor R-package DESeq2 (release 3.10) and RStudio. A differential gene expression model was deployed between the CK− and CK+ group using the Wald test. Calculated log2 FCs were shrank using "ashr" to account for lowly expressed differentially expressed genes, using the integrated LFCshrink function in DESeq2. Variance stabilized counts were calculated to account for heteroscedasticity and used for PCA as well as heatmap generation. The Bioconductor package EnhancedVolcano was further used to visualize significantly differentially expressed genes according to their FC for CK− versus CK+.[125] To receive an overview of the distribution of mapped read counts according to their biotype for each group, DESeq2 normalized group counts were aggregated using Python's Pandas module (groupby) and visualized as a pie chart. Analysis scripts are accessible via GitHub.

miRNA-mapping mirdeep2

miRNAs were aligned to the human reference genome (GRCh38) using bowtie with default parameters using the mapper.pl module. Aligned reads with a length below 16 were removed from the analysis. Quantification of miRNAs was performed with the quantifier.pl module provided by mirdeep2 against the mature human miRNA reference as well as the hairpin-precursor reference provided by miRbase.org.[126] Differential abundance analysis of miRNAs was performed using DESeq2 on raw counts. Significance threshold following false discovery rate (FDR) correction was set to 0.1 and the log2 FC was shrunk using the "ashr" shrinkage function.

miRNA target space analysis

Most abundantly expressed miRNAs preselected from the differential abundance analysis (qPCR panel) were subjected to target space enrichment analysis. For this, high-confidence miRNA targets were queried using the DIANA microT-CDS v5.0 algorithm with the target threshold set to 0.9.[127] Extracted targets per miRNAs were then subject to g::Profiler ontology analysis using the ontology space biological pathways (GO:BP). miRNAs were then clustered using hierarchical clustering based on similar pathways enriched and visualized using the clustermap function integrated in Python's "seaborn" module.

Ontology analysis (g::Profiler)

Ontology analysis was performed using the integrated g::Profiler API querying the ontology spaces (GO:BP, GO:CC, GO:MF, and KEGG). The significance of pathways was tested using the integrated g:SCS method for computing multiple comparisons and only best-per-parent pathways were used for further analysis.

SUPPLEMENTAL INFORMATION

Supplemental information can be found online at https://doi.org/10.1016/j.omtn.2022.04.035.

ACKNOWLEDGMENTS

This research was funded by the Austrian Science Funds (FWF ZFP326120 and FWF TAI 411-B) to A.H. We would like to thank Dr. Martin Offterdinger (Division of Neurobiochemistry/Biooptics, Medical University of Innsbruck), Dr. Alexander Jesacher (Institute of Biomedical Physics, Medical University of Innsbruck), and Dr. Philipp Zelger (University Clinic for Hearing, Voice and Speech Disorders, Tirol Kliniken Innsbruck) for their technical support, and Dr. Mariana E.G. de Araujo (Division of Cell Biology, Medical University of Innsbruck) for providing the LC3B antibody. A special thanks to Dr. Harald Mischak for helpful discussions.

AUTHOR CONTRIBUTIONS

A.H. conceived and supervised the study, and also organized, wrote, and edited the manuscript. G.R. planned and performed the experiments, analyzed the data, wrote the original draft, and organized and edited the manuscript. M.Z. analyzed the RNA-seq data, and wrote and edited the manuscript. R.K. processed the urinary exosome samples. M.H. performed the RNA-seq analysis. A.K. supervised the RNA-seq analysis. M.W.H. analyzed the exosome samples for TEM. J.V., P.P., M.W.H., H.S., K.K.K., M.K., M.R., and G.M. provided valuable ideas for this study and edited the manuscript. M.R. and G.M. also provided the urine samples.

DECLARATION OF INTERESTS

P.P. is an employee at Delta 4 GmbH, Austria, and R.K. is an employee of Commonwealth Serum Laboratories Behring, Switzerland. All other authors declare no competing interests.

REFERENCES

1. Inker, L.A., Astor, B.C., Fox, C.H., Isakova, T., Lash, J.P., Peralta, C.A., et al. (2014). KDOQI US commentary on the 2012 KDIGO clinical practice guideline for the evaluation and management of CKD. Am J. Kidney Dis *63* (5), 713–735. https://doi.org/10.1053/j.ajkd.2014.01.416.
2. Romagnani, P., Remuzzi, G., Glassock, R., Levin, A., Jager, K.J., Tonelli, M., Massy, Z., Wanner, C., and Anders, H.J. (2017). Chronic kidney disease. Nat. Rev. Dis. Primers *3*, 17088. https://doi.org/10.1038/nrdp.2017.88.
3. Bikbov, B., Purcell, C.A., Levey, A.S., Smith, M., Abdoli, A., Abebe, M., Adebayo, O.M., Afarideh, M., Agarwal, S.K., Agudelo-Botero, M., et al. (2020). Global, regional, and national burden of chronic kidney disease, 1990–2017: a systematic analysis for the Global Burden of Disease Study 2017. Lancet *395*, 709–733. https://doi.org/10.1016/S0140-6736(20)30045-3.
4. Meng, X.M., Nikolic-Paterson, D.J., and Lan, H.Y. (2014). Inflammatory processes in renal fibrosis. Nat. Rev. Nephrol. *10*, 493–503. https://doi.org/10.1038/nrneph.2014.114.
5. Nangaku, M. (2006). Chronic hypoxia and tubulointerstitial injury: a final common pathway to end-stage renal failure. J. Am. Soc. Nephrol. *17*, 17–25. https://doi.org/10.1681/asn.2005070757.
6. Grgic, I., Campanholle, G., Bijol, V., Wang, C., Sabbisetti, V.S., Ichimura, T., Humphreys, B.D., and Bonventre, J.V. (2012). Targeted proximal tubule injury triggers interstitial fibrosis and glomerulosclerosis. Kidney Int. *82*, 172–183. https://doi.org/10.1038/ki.2012.20.
7. Gewin, L., Zent, R., and Pozzi, A. (2017). Progression of chronic kidney disease: too much cellular talk causes damage. Kidney Int. *91*, 552–560. https://doi.org/10.1016/j.kint.2016.08.025.
8. Yang, L., Besschetnova, T.Y., Brooks, C.R., Shah, J.V., and Bonventre, J.V. (2010). Epithelial cell cycle arrest in G2/M mediates kidney fibrosis after injury. Nat. Med. *16*, 535–543. https://doi.org/10.1038/nm.2144.
9. Wieser, M., Stadler, G., Jennings, P., Streubel, B., Pfaller, W., Ambros, P., Riedl, C., Katinger, H., Grillari, J., and Grillari-Voglauer, R. (2008). hTERT alone immortalizes epithelial cells of renal proximal tubules without changing their functional characteristics. Am. J. Physiol. Ren. Physiol. *295*, F1365–F1375. https://doi.org/10.1152/ajprenal.90405.2008.
10. Slyne, J., Slattery, C., McMorrow, T., and Ryan, M.P. (2015). New developments concerning the proximal tubule in diabetic nephropathy: in vitro models and mechanisms. Nephrol. Dial. Transpl. *30*. iv60–67. https://doi.org/10.1093/ndt/gfv264.
11. Sarkozi, R., Corazza, U., Osterkamp, J.P., Pirklbauer, M., Mayer, G., and Schramek, H. (2015). Synergistic induction of CCL2/MCP-1 expression driven by oncostatin M and IL-1β in human proximal tubular cells depends on STAT3 and p65 NFκB/RelA. Physiol. Rep. *3*, e12298. https://doi.org/10.14814/phy2.12298.
12. Qi, W., Chen, X., Polhill, T.S., Sumual, S., Twigg, S., Gilbert, R.E., and Pollock, C.A. (2006). TGF-β_1 induces IL-8 and MCP-1 through a connective tissue growth factor-independent pathway. Am. J. Physiol. Ren. Physiol. *290*, F703–F709. https://doi.org/10.1152/ajprenal.00254.2005.
13. Sarkozi, R., Hauser, C., Noppert, S.J., Kronbichler, A., Pirklbauer, M., Haller, V.M., Grillari, J., Grillari-Voglauer, R., Mayer, G., and Schramek, H. (2011). Oncostatin M is a novel inhibitor of TGF-β1-induced matricellular protein expression. Am. J. Physiol. Ren. Physiol. *301*, F1014–F1025. https://doi.org/10.1152/ajprenal.00123.2011.
14. Chevalier, R.L. (2016). The proximal tubule is the primary target of injury and progression of kidney disease: role of the glomerulotubular junction. Am. J. Physiol. Ren. Physiol. *311*, F145–F161. https://doi.org/10.1152/ajprenal.00164.2016.
15. Takaori, K., and Yanagita, M. (2016). Insights into the mechanisms of the acute kidney injury-to-chronic kidney disease continuum. Nephron *134*, 172–176. https://doi.org/10.1159/000448081.
16. Seok, J., Warren, H.S., Cuenca, A.G., Mindrinos, M.N., Baker, H.V., Xu, W., Richards, D.R., McDonald-Smith, G.P., Gao, H., Hennessy, L., et al. (2013). Genomic responses in mouse models poorly mimic human inflammatory diseases. Proc. Natl. Acad. Sci. U S A. *110*, 3507–3512. https://doi.org/10.1073/pnas.1222878110.
17. Caby, M.P., Lankar, D., Vincendeau-Scherrer, C., Raposo, G., and Bonnerot, C. (2005). Exosomal-like vesicles are present in human blood plasma. Int. Immunol. *17*, 879–887. https://doi.org/10.1093/intimm/dxh267.
18. Wang, L., Skotland, T., Berge, V., Sandvig, K., and Llorente, A. (2017). Exosomal proteins as prostate cancer biomarkers in urine: from mass spectrometry discovery to immunoassay-based validation. Eur. J. Pharm. Sci. *98*, 80–85. https://doi.org/10.1016/j.ejps.2016.09.023.
19. Khurana, R., Ranches, G., Schafferer, S., Lukasser, M., Rudnicki, M., Mayer, G., and Huttenhofer, A. (2017). Identification of urinary exosomal noncoding RNAs as novel biomarkers in chronic kidney disease. RNA *23*, 142–152. https://doi.org/10.1261/rna.058834.116.
20. Barreiro, K., Huber, T.B., and Holthofer, H. (2021). Urinary extracellular vesicles magic particles for biomarker discovery. Adv. Exp. Med. Biol. *1306*, 29–40. https://doi.org/10.1007/978-3-030-63908-2_3.
21. Johnstone, R.M., Adam, M., Hammond, J.R., Orr, L., and Turbide, C. (1987). Vesicle formation during reticulocyte maturation. Association of plasma membrane activities with released vesicles (exosomes). J. Biol. Chem. *262*, 9412–9420. https://doi.org/10.1016/s0021-9258(18)48095-7.
22. Doyle, L.M., and Wang, M.Z. (2019). Overview of extracellular vesicles, their origin, composition, purpose, and methods for exosome isolation and analysis. Cells *8*, 727. https://doi.org/10.3390/cells8070727.
23. Raiborg, C., and Stenmark, H. (2009). The ESCRT machinery in endosomal sorting of ubiquitylated membrane proteins. Nature *458*, 445–452. https://doi.org/10.1038/nature07961.
24. Larios, J., Mercier, V., Roux, A., and Gruenberg, J. (2020). ALIX- and ESCRT-III-dependent sorting of tetraspanins to exosomes. J. Cell Biol. *219*, e201904113. https://doi.org/10.1083/jcb.201904113.
25. Trajkovic, K., Hsu, C., Chiantia, S., Rajendran, L., Wenzel, D., Wieland, F., Schwille, P., Brugger, B., and Simons, M. (2008). Ceramide triggers budding of exosome vesicles into multivesicular endosomes. Science *319*, 1244–1247. https://doi.org/10.1126/science.1153124.

26. Colombo, M., Moita, C., van Niel, G., Kowal, J., Vigneron, J., Benaroch, P., Manel, N., Moita, L.F., Thery, C., and Raposo, G. (2013). Analysis of ESCRT functions in exosome biogenesis, composition and secretion highlights the heterogeneity of extracellular vesicles. J. Cell Sci. *126*, 5553–5565. https://doi.org/10.1242/jcs.128868.

27. Luberto, C., Kraveka, J.M., and Hannun, Y.A. (2002). Ceramide regulation of apoptosis versus differentiation: a walk on a fine line. Lessons from neurobiology. Neurochem. Res. *27*, 609–617. https://doi.org/10.1023/a:1020267831851.

28. Guo, E.Z., and Xu, Z. (2015). Distinct mechanisms of recognizing endosomal sorting complex required for transport III (ESCRT-III) protein IST1 by different microtubule interacting and trafficking (MIT) domains. J. Biol. Chem. *290*, 8396–8408. https://doi.org/10.1074/jbc.m114.607903.

29. Datta, A., Kim, H., Lal, M., McGee, L., Johnson, A., Moustafa, A.A., Jones, J.C., Mondal, D., Ferrer, M., and Abdel-Mageed, A.B. (2017). Manumycin A suppresses exosome biogenesis and secretion via targeted inhibition of Ras/Raf/ERK1/2 signaling and hnRNP H1 in castration-resistant prostate cancer cells. Cancer Lett. *408*, 73–81. https://doi.org/10.1016/j.canlet.2017.08.020.

30. Catalano, M., and O'Driscoll, L. (2020). Inhibiting extracellular vesicles formation and release: a review of EV inhibitors. J. Extracell Vesicles *9*, 1703244. https://doi.org/10.1080/20013078.2019.1703244.

31. Zhou, H., Pisitkun, T., Aponte, A., Yuen, P.S., Hoffert, J.D., Yasuda, H., Hu, X., Chawla, L., Shen, R.F., Knepper, M.A., et al. (2006). Exosomal Fetuin-A identified by proteomics: a novel urinary biomarker for detecting acute kidney injury. Kidney Int. *70*, 1847–1857. https://doi.org/10.1038/sj.ki.5001874.

32. Pisitkun, T., Hoffert, J.D., Yu, M.J., and Knepper, M.A. (2007). Tandem mass spectrometry in physiology. Physiology (Bethesda) *22*, 390–400. https://doi.org/10.1152/physiol.00025.2007.

33. Jella, K.K., Nasti, T.H., Li, Z., Malla, S.R., Buchwald, Z.S., and Khan, M.K. (2018). Exosomes, their biogenesis and role in inter-cellular communication, tumor microenvironment and cancer immunotherapy. Vaccines 6, 69.

34. Harrell, C.R., Fellabaum, C., Jovicic, N., Djonov, V., Arsenijevic, N., and Volarevic, V. (2019). Molecular mechanisms responsible for therapeutic potential of mesenchymal stem cell-derived secretome. Cells *8*, 467. https://doi.org/10.3390/cells8050467.

35. Huttenhofer, A., and Mayer, G. (2017). Circulating miRNAs as biomarkers of kidney disease. Clin. Kidney J. *10*, 27–29. https://doi.org/10.1093/ckj/sfw075.

36. Rudnicki, M., Perco, P., D'haene, B., Leierer, J., Heinzel, A., Muhlberger, I., Schweibert, N., Sunzenauer, J., Regele, H., Kronbichler, A., et al. (2016). Renal microRNA- and RNA-profiles in progressive chronic kidney disease. Eur. J. Clin. Invest. *46*, 213–226. https://doi.org/10.1111/eci.12585.

37. Musante, L., Tataruch, D.E., and Holthofer, H. (2014). Use and isolation of urinary exosomes as biomarkers for diabetic nephropathy. Front. Endocrinol. (Lausanne) *5*, 149. https://doi.org/10.3389/fendo.2014.00149.

38. Gudehithlu, K.P., Garcia-Gomez, I., Vernik, J., Brecklin, C., Kraus, M., Cimbaluk, D.J., Hart, P., Dunea, G., Arruda, J.A., and Singh, A.K. (2015). In diabetic kidney disease urinary exosomes better represent kidney specific protein alterations than whole urine. Am. J. Nephrol. *42*, 418–424. https://doi.org/10.1159/000443539.

39. Shurtleff, M.J., Temoche-Diaz, M.M., Karfilis, K.V., Ri, S., and Schekman, R. (2016). Y-box protein 1 is required to sort microRNAs into exosomes in cells and in a cell-free reaction. Elife *5*, e19276. https://doi.org/10.7554/elife.19276.

40. Willms, E., Johansson, H.J., Mager, I., Lee, Y., Blomberg, K.E.M., Sadik, M., Alaarg, A., Smith, C.E., Lehtio, J., El Andaloussi, S., et al. (2016). Cells release subpopulations of exosomes with distinct molecular and biological properties. Sci. Rep. *6*, 22519. https://doi.org/10.1038/srep22519.

41. Bignotti, E., Calza, S., Tassi, R.A., Zanotti, L., Bandiera, E., Sartori, E., Odicino, F.E., Ravaggi, A., Todeschini, P., and Romani, C. (2016). Identification of stably expressed reference small non-coding RNAs for microRNA quantification in high-grade serous ovarian carcinoma tissues. J. Cell Mol. Med. *20*, 2341–2348. https://doi.org/10.1111/jcmm.12927.

42. Abraham, S., Montoya, R.T., Edelstein, L.C., McKenzie, S.E., and Bray, P.F. (2012). Identification of reference genes for miRNA profiling in hematopoietic cell lineages. Blood *120*, 3330.

43. Lange, T., Stracke, S., Rettig, R., Lendeckel, U., Kuhn, J., Schluter, R., Rippe, V., Endlich, K., and Endlich, N. (2017). Identification of miR-16 as an endogenous reference gene for the normalization of urinary exosomal miRNA expression data from CKD patients. PLoS One *12*, e0183435. https://doi.org/10.1371/journal.pone.0183435.

44. Donati, S., Ciuffi, S., and Brandi, M.L. (2019). Human circulating miRNAs real-time qRT-PCR-based analysis: an overview of endogenous reference genes used for data normalization. Int. J. Mol. Sci. *20*, 4353. https://doi.org/10.3390/ijms20184353.

45. Rudnicki, M., Schweibert, N., Beckers, A., Schneeberger, S., Königsrainer, A., Böhmig, G., Vandesompele, J., Mayer, G., and Leierer, J. (2016). SP248endogenous controls for miRNA analysis in rneal cells and tissue. Nephrol. Dial. Transpl. *31*, i169–i170. https://doi.org/10.1093/ndt/gfw163.29.

46. Knepper, M.A., and Pisitkun, T. (2007). Exosomes in urine: who would have thought. Kidney Int. *72*, 1043–1045. https://doi.org/10.1038/sj.ki.5002510.

47. Ranches, G., Lukasser, M., Schramek, H., Ploner, A., Stasyk, T., Mayer, G., Mayer, G., and Huttenhofer, A. (2017). In vitro selection of cell-internalizing DNA aptamers in a model system of inflammatory kidney disease. Mol. Ther. Nucleic Acids *8*, 198–210. https://doi.org/10.1016/j.omtn.2017.06.018.

48. Tam, J., and Merino, D. (2015). Stochastic optical reconstruction microscopy (STORM) in comparison with stimulated emission depletion (STED) and other imaging methods. J. Neurochem. *135*, 643–658. https://doi.org/10.1111/jnc.13257.

49. Nahidiazar, L., Agronskaia, A.V., Broertjes, J., van den Broek, B., and Jalink, K. (2016). Optimizing imaging conditions for demanding multi-color super resolution localization microscopy. PLoS One *11*, e0158884. https://doi.org/10.1371/journal.pone.0158884.

50. Graner, M.W., Alzate, O., Dechkovskaia, A.M., Keene, J.D., Sampson, J.H., Mitchell, D.A., and Bigner, D.D. (2009). Proteomic and immunologic analyses of brain tumor exosomes. FASEB J. *23*, 1541–1557. https://doi.org/10.1096/fj.08-122184.

51. Wunsch, B.H., Smith, J.T., Gifford, S.M., Wang, C., Brink, M., Bruce, R.L., Austin, R.H., Stolovitzky, G., and Astier, Y. (2016). Nanoscale lateral displacement arrays for the separation of exosomes and colloids down to 20 nm. Nat. Nanotechnol. *11*, 936–940. https://doi.org/10.1038/nnano.2016.134.

52. Kosaka, N., Iguchi, H., Yoshioka, Y., Takeshita, F., Matsuki, Y., and Ochiya, T. (2010). Secretory mechanisms and intercellular transfer of microRNAs in living cells. J. Biol. Chem. *285*, 17442–17452. https://doi.org/10.1074/jbc.m110.107821.

53. Kosaka, N., and Ochiya, T. (2012). Unraveling the mystery of cancer by secretory microRNA: horizontal microRNA transfer between living cells. Front. Genet. *2*, 97. https://doi.org/10.3389/fgene.2011.00097.

54. Arenz, C., Thutewohl, M., Block, O., Waldmann, H., Altenbach, H.J., and Giannis, A. (2001). Manumycin A and its analogues are irreversible inhibitors of neutral sphingomyelinase. Chembiochem *2*, 141–143. https://doi.org/10.1002/1439-7633(20010202)2:2<141::aid-cbic141>3.0.co;2-p.

55. Liu, B.C., Tang, T.T., Lv, L.L., and Lan, H.Y. (2018). Renal tubule injury: a driving force toward chronic kidney disease. Kidney Int. *93*, 568–579. https://doi.org/10.1016/j.kint.2017.09.033.

56. Chandrasekaran, K., Karolina, D.S., Sepramaniam, S., Armugam, A., Marelyn Wintour, E., Bertram, J.F., and Jeyaseelan, K. (2012). Role of microRNAs in kidney homeostasis and disease. Kidney Int. *81*, 617–627. https://doi.org/10.1038/ki.2011.448.

57. Connor, K.L., Teenan, O., Cairns, C., Banwell, V., Thomas, R.A., Rodor, J., Finnie, S., Pius, R., Tannahill, G.M., Sahni, V., et al. (2020). Identifying cell-enriched miRNAs in kidney injury and repair. JCI Insight *5*, e140399. https://doi.org/10.1172/jci.insight.140399.

58. Valadi, H., Ekstrom, K., Bossios, A., Sjostrand, M., Lee, J.J., and Lotvall, J.O. (2007). Exosome-mediated transfer of mRNAs and microRNAs is a novel mechanism of genetic exchange between cells. Nat. Cell Biol. *9*, 654–659. https://doi.org/10.1038/ncb1596.

59. O'Brien, K., Breyne, K., Ughetto, S., Laurent, L.C., and Breakefield, X.O. (2020). RNA delivery by extracellular vesicles in mammalian cells and its applications. Nat. Rev. Mol. Cell Biol. *21*, 585–606. https://doi.org/10.1038/s41580-020-0251-y.

60. Lin, F., Zeng, Z., Song, Y., Li, L., Wu, Z., Zhang, X., Li, Z., Ke, X., and Hu, X. (2019). YBX-1 mediated sorting of miR-133 into hypoxia/reoxygenation-induced EPC-derived exosomes to increase fibroblast angiogenesis and MEndoT. Stem Cell Res. Ther. *10*, 263. https://doi.org/10.1186/s13287-019-1377-8.

61. Teng, Y., Ren, Y., Hu, X., Mu, J.Y., Samykutty, A., Zhuang, X.Y., Deng, Z.B., Kumar, A., Zhang, L.F., Merchant, M.L., et al. (2017). MVP-mediated exosomal sorting of miR-193a promotes colon cancer progression. Nat. Commun. *8*, 14448. https://doi.org/10.1038/ncomms14448.

62. Lu, P., Li, H., Li, N., Singh, R.N., Bishop, C.E., Chen, X., and Lu, B. (2017). MEX3C interacts with adaptor-related protein complex 2 and involves in miR-451a exosomal sorting. PLoS One *12*, e0185992. https://doi.org/10.1371/journal.pone.0185992.

63. Villarroya-Beltri, C., Gutierrez-Vazquez, C., Sanchez-Cabo, F., Perez-Hernandez, D., Vazquez, J., Martin-Cofreces, N., Martinez-Herrera, D.J., Pascual-Montano, A., Mittelbrunn, M., and Sanchez-Madrid, F. (2013). Sumoylated hnRNPA2B1 controls the sorting of miRNAs into exosomes through binding to specific motifs. Nat. Commun. *4*, 2980. https://doi.org/10.1038/ncomms3980.

64. Groot, M., and Lee, H. (2020). Sorting mechanisms for MicroRNAs into extracellular vesicles and their associated diseases. Cells *9*, 1044. https://doi.org/10.3390/cells9041044.

65. Jia, Y., Guan, M., Zheng, Z., Zhang, Q., Tang, C., Xu, W., Xiao, Z., Wang, L., and Xue, Y. (2016). miRNAs in urine extracellular vesicles as predictors of early-stage diabetic nephropathy. J. Diabetes Res. *2016*, 1–10. https://doi.org/10.1155/2016/7932765.

66. Min, Q.H., Chen, X.M., Zou, Y.Q., Zhang, J., Li, J., Wang, Y., Li, S.Q., Gao, Q.F., Sun, F., Liu, J., et al. (2018). Differential expression of urinary exosomal microRNAs in IgA nephropathy. J. Clin. Lab. Anal. *32*, e22226. https://doi.org/10.1002/jcla.22226.

67. Perez-Hernandez, J., Olivares, D., Forner, M.J., Ortega, A., Solaz, E., Martinez, F., Chaves, F.J., Redon, J., and Cortes, R. (2018). Urinary exosome miR-146a is a potential marker of albuminuria in essential hypertension. J. Transl. Med. *16*, 228. https://doi.org/10.1186/s12967-018-1604-6.

68. Zang, J., Maxwell, A.P., Simpson, D.A., and McKay, G.J. (2019). Differential expression of urinary exosomal MicroRNAs miR-21-5p and miR-30b-5p in individuals with diabetic kidney disease. Sci. Rep. *9*, 10900. https://doi.org/10.1038/s41598-019-47504-x.

69. Dong, L., Zhong, X., He, J., Zhang, L., Bai, K., Xu, W., Wang, T., and Huang, X. (2016). Supplementation of tributyrin improves the growth and intestinal digestive and barrier functions in intrauterine growth-restricted piglets. Clin. Nutr. *35*, 399–407. https://doi.org/10.1016/j.clnu.2015.03.002.

70. Gouin, K., Peck, K., Antes, T., Johnson, J.L., Li, C., Vaturi, S.D., Middleton, R., de Couto, G., Walravens, A.S., Rodriguez-Borlado, L., et al. (2017). A comprehensive method for identification of suitable reference genes in extracellular vesicles. J. Extracell. Vesicles *6*, 1347019. https://doi.org/10.1080/20013078.2017.1347019.

71. Liu, L., Pang, X.L., Shang, W.J., Xie, H.C., Wang, J.X., and Feng, G.W. (2018). Over-expressed microRNA-181a reduces glomerular sclerosis and renal tubular epithelial injury in rats with chronic kidney disease via down-regulation of the TLR/NF-κB pathway by binding to CRY1. Mol. Med. *24*, 49. https://doi.org/10.1186/s10020-018-0045-2.

72. Chung, K.W., Dhillon, P., Huang, S., Sheng, X., Shrestha, R., Qiu, C., Kaufman, B.A., Park, J., Pei, L., Baur, J., et al. (2019). Mitochondrial damage and activation of the STING pathway lead to renal inflammation and fibrosis. Cell Metab. *30*, 784–799.e5. https://doi.org/10.1016/j.cmet.2019.08.003.

73. Liu, J., Jia, Z., and Gong, W. (2021). Circulating mitochondrial DNA stimulates innate immune signaling pathways to mediate acute kidney injury. Front. Immunol. *12*, 680648. https://doi.org/10.3389/fimmu.2021.680648.

74. Borges, F.T., Melo, S.A., Ozdemir, B.C., Kato, N., Revuelta, I., Miller, C.A., Gattone, V.H., 2nd, LeBleu, V.S., and Kalluri, R. (2013). TGF-β1–Containing exosomes from injured epithelial cells activate fibroblasts to initiate tissue regenerative responses and fibrosis. J. Am. Soc. Nephrol. *24*, 385–392. https://doi.org/10.1681/asn.2012101031.

75. Thongboonkerd, V. (2020). Roles for exosome in various kidney diseases and disorders. Front. Pharmacol. *10*, 1655. https://doi.org/10.3389/fphar.2019.01655.

76. Kwon, S.H. (2019). Extracellular vesicles in renal physiology and clinical applications for renal disease. Korean J. Intern. Med. *34*, 470–479. https://doi.org/10.3904/kjim.2019.108.

77. Erozenci, L.A., Bottger, F., Bijnsdorp, I.V., and Jimenez, C.R. (2019). Urinary exosomal proteins as (pan-)cancer biomarkers: insights from the proteome. FEBS Lett. *593*, 1580–1597. https://doi.org/10.1002/1873-3468.13487.

78. Akbar, N., Azzimato, V., Choudhury, R.P., and Aouadi, M. (2019). Extracellular vesicles in metabolic disease. Diabetologia *62*, 2179–2187. https://doi.org/10.1007/s00125-019-05014-5.

79. Lam, C.W.K. (2009). 2. Inflammation, cytokines and chemokines in chronic kidney disease. EJIFCC *20*, 12–20.

80. Sarkozi, R., Flucher, K., Haller, V.M., Pirklbauer, M., Mayer, G., and Schramek, H. (2012). Oncostatin M inhibits TGF-β1-induced CTGF expression via STAT3 in human proximal tubular cells. Biochem. Biophys. Res. Commun. *424*, 801–806. https://doi.org/10.1016/j.bbrc.2012.07.042.

81. Akhmetshina, A., Palumbo, K., Dees, C., Bergmann, C., Venalis, P., Zerr, P., Horn, A., Kireva, T., Beyer, C., Zwerina, J., et al. (2012). Activation of canonical Wnt signalling is required for TGF-beta-mediated fibrosis. Nat. Commun. *3*, 735. https://doi.org/10.1038/ncomms1734.

82. Park, J.I., Venteicher, A.S., Hong, J.Y., Choi, J., Jun, S., Shkreli, M., Chang, W., Meng, Z., Cheung, P., Ji, H., et al. (2009). Telomerase modulates Wnt signalling by association with target gene chromatin. Nature *460*, 66–72. https://doi.org/10.1038/nature08137.

83. Zhang, C., Chen, P., Fei, Y., Liu, B., Ma, K., Fu, X., Zhao, Z., Sun, T., and Sheng, Z. (2012). Wnt/β-catenin signaling is critical for dedifferentiation of aged epidermal cells in vivo and in vitro. Aging Cell *11*, 14–23. https://doi.org/10.1111/j.1474-9726.2011.00753.x.

84. Listerman, I., Gazzaniga, F.S., and Blackburn, E.H. (2014). An investigation of the effects of the core protein telomerase reverse transcriptase on Wnt signaling in breast cancer cells. Mol. Cell Biol. *34*, 280–289. https://doi.org/10.1128/mcb.00844-13.

85. Yang, W., Luo, Y., Yang, S., Zeng, M., Zhang, S., Liu, J., Han, Y., Liu, Y., Zhu, X., Wu, H., et al. (2018). Ectopic lipid accumulation: potential role in tubular injury and inflammation in diabetic kidney disease. Clin. Sci. (Lond) *132*, 2407–2422. https://doi.org/10.1042/cs20180702.

86. Jing, H., Tang, S., Lin, S., Liao, M., Chen, H., and Zhou, J. (2019). The role of extracellular vesicles in renal fibrosis. Cell Death Dis. *10*, 367. https://doi.org/10.1038/s41419-019-1605-2.

87. Yang, Y., Boza-Serrano, A., Dunning, C.J.R., Clausen, B.H., Lambertsen, K.L., and Deierborg, T. (2018). Inflammation leads to distinct populations of extracellular vesicles from microglia. J. Neuroinflammation *15*, 168. https://doi.org/10.1186/s12974-018-1204-7.

88. Rieu, S., Geminard, C., Rabesandratana, H., Sainte-Marie, J., and Vidal, M. (2000). Exosomes released during reticulocyte maturation bind to fibronectin via integrin α4β1. Eur. J. Biochem. *267*, 583–590. https://doi.org/10.1046/j.1432-1327.2000.01036.x.

89. Blanc, L., and Vidal, M. (2010). Reticulocyte membrane remodeling: contribution of the exosome pathway. Curr. Opin. Hematol. *17*, 1–183. https://doi.org/10.1097/moh.0b013e328337b4e3.

90. Vidal, M. (2019). Exosomes: revisiting their role as "garbage bags". Traffic *20*, 815–828. https://doi.org/10.1111/tra.12687.

91. Takahashi, A., Okada, R., Nagao, K., Kawamata, Y., Hanyu, A., Yoshimoto, S., Takasugi, M., Watanabe, S., Kanemaki, M.T., Obuse, C., and Hara, E. (2017). Exosomes maintain cellular homeostasis by excreting harmful DNA from cells. Nat. Commun. *8*, 15287. https://doi.org/10.1038/ncomms15287.

92. Lv, L.L., Feng, Y., Tang, T.T., and Liu, B.C. (2019). New insight into the role of extracellular vesicles in kidney disease. J. Cell Mol. Med. *23*, 731–739. https://doi.org/10.1111/jcmm.14101.

93. Thomas, M.J., Fraser, D.J., and Bowen, T. (2018). Biogenesis, stabilization, and transport of microRNAs in kidney health and disease. Noncoding RNA *4*, 30. https://doi.org/10.3390/ncrna4040030.

94. Fan, P.C., Chen, C.C., Chen, Y.C., Chang, Y.S., and Chu, P.H. (2016). MicroRNAs in acute kidney injury. Hum. Genomics *10*, 29. https://doi.org/10.1186/s40246-016-0085-z.

95. Melkonyan, H.S., Feaver, W.J., Meyer, E., Scheinker, V., Shekhtman, E.M., Xin, Z., and Umansky, S.R. (2008). Transrenal nucleic acids: from proof of principle to clinical tests. Ann. N. Y Acad. Sci. *1137*, 73–81. https://doi.org/10.1196/annals.1448.015.

96. Sun, I.O., and Lerman, L.O. (2019). Urinary microRNA in kidney disease: utility and roles. Am. J. Physiol. Ren. Physiol. *316*, F785–F793. https://doi.org/10.1152/ajprenal.00368.2018.

97. Gross, J.L., de Azevedo, M.J., Silveiro, S.P., Canani, L.H., Caramori, M.L., and Zelmanovitz, T. (2005). Diabetic nephropathy: diagnosis, prevention, and treatment. Diabetes Care *28*, 164–176. https://doi.org/10.2337/diacare.28.1.164.

98. Ryzhov, S., Matafonov, A., Galindo, C.L., Zhang, Q., Tran, T.L., Lenihan, D.J., Lenneman, C.G., Feoktistov, I., and Sawyer, D.B. (2017). ERBB signaling attenuates proinflammatory activation of nonclassical monocytes. Am. J. Physiol. Heart Circ. Physiol. *312*, H907–H918. https://doi.org/10.1152/ajpheart.00486.2016.

99. Hawkins, P.T., and Stephens, L.R. (2015). PI3K signalling in inflammation. Biochim. Biophys. Acta *1851*, 882–897. https://doi.org/10.1016/j.bbalip.2014.12.006.

100. Nolte-'t Hoen, E.N.M., Buermans, H.P.J., Waasdorp, M., Stoorvogel, W., Wauben, M.H.M., and 't Hoen, P.A.C. (2012). Deep sequencing of RNA from immune cell-derived vesicles uncovers the selective incorporation of small non-coding RNA biotypes with potential regulatory functions. Nucleic Acids Res. *40*, 9272–9285. https://doi.org/10.1093/nar/gks658.

101. de Jong, O.G., Verhaar, M.C., Chen, Y., Vader, P., Gremmels, H., Posthuma, G., Schiffelers, R.M., Gucek, M., and van Balkom, B.W. (2012). Cellular stress conditions are reflected in the protein and RNA content of endothelial cell-derived exosomes. J. Extracell Vesicles *1*, 18396. https://doi.org/10.3402/jev.v1i0.18396.

102. Mears, R., Craven, R.A., Hanrahan, S., Totty, N., Upton, C., Young, S.L., Patel, P., Selby, P.J., and Banks, R.E. (2004). Proteomic analysis of melanoma-derived exosomes by two-dimensional polyacrylamide gel electrophoresis and mass spectrometry. Proteomics *4*, 4019–4031. https://doi.org/10.1002/pmic.200400876.

103. Soubannier, V., McLelland, G.L., Zunino, R., Braschi, E., Rippstein, P., Fon, E.A., and McBride, H.M. (2012). A vesicular transport pathway shuttles cargo from mitochondria to lysosomes. Curr. Biol. *22*, 135–141. https://doi.org/10.1016/j.cub.2011.11.057.

104. Politi, Y., Gal, L., Kalifa, Y., Ravid, L., Elazar, Z., and Arama, E. (2014). Paternal mitochondrial destruction after fertilization is mediated by a common endocytic and autophagic pathway in Drosophila. Dev. Cell *29*, 305–320. https://doi.org/10.1016/j.devcel.2014.04.005.

105. Liou, W., Geuze, H.J., Geelen, M.J., and Slot, J.W. (1997). The autophagic and endocytic pathways converge at the nascent autophagic vacuoles. J. Cell Biol. *136*, 61–70. https://doi.org/10.1083/jcb.136.1.61.

106. Bhargava, P., and Schnellmann, R.G. (2017). Mitochondrial energetics in the kidney. Nat. Rev. Nephrol. *13*, 629–646. https://doi.org/10.1038/nrneph.2017.107.

107. Galvan, D.L., Green, N.H., and Danesh, F.R. (2017). The hallmarks of mitochondrial dysfunction in chronic kidney disease. Kidney Int. *92*, 1051–1057. https://doi.org/10.1016/j.kint.2017.05.034.

108. Bai, M., Chen, H., Ding, D., Song, R., Lin, J., Zhang, Y., Guo, Y., Chen, S., Ding, G., Zhang, Y., et al. (2019). MicroRNA-214 promotes chronic kidney disease by disrupting mitochondrial oxidative phosphorylation. Kidney Int. *95*, 1389–1404. https://doi.org/10.1016/j.kint.2018.12.028.

109. Wang, X., Tang, D., Zou, Y., Wu, X., Chen, Y., Li, H., Chen, S., Shi, Y., and Niu, H. (2019). A mitochondrial-targeted peptide ameliorated podocyte apoptosis through a HOCl-alb-enhanced and mitochondria-dependent signalling pathway in diabetic rats and in vitro. J. Enzyme Inhib. Med. Chem. *34*, 394–404. https://doi.org/10.1080/14756366.2018.1488697.

110. Abate, M., Festa, A., Falco, M., Lombardi, A., Luce, A., Grimaldi, A., Zappavigna, S., Sperlongano, P., Irace, C., Caraglia, M., et al. (2020). Mitochondria as playmakers of apoptosis, autophagy and senescence. Semin. Cell Dev. Biol. *98*, 139–153. https://doi.org/10.1016/j.semcdb.2019.05.022.

111. Casalena, G., Daehn, I., and Bottinger, E. (2012). Transforming growth factor-β, bioenergetics, and mitochondria in renal disease. Semin. Nephrol. *32*, 295–303. https://doi.org/10.1016/j.semnephrol.2012.04.009.

112. Herrera, B., Alvarez, A.M., Sanchez, A., Fernandez, M., Roncero, C., Benito, M., and Fabregat, I. (2001). Reactive oxygen species (ROS) mediates the mitochondrial-dependent apoptosis induced by transforming growth factor ß in fetal hepatocytes. FASEB J. *15*, 741–751. https://doi.org/10.1096/fj.00-0267com.

113. Ding, Y., Kim, S., Lee, S.Y., Koo, J.K., Wang, Z., and Choi, M.E. (2014). Autophagy regulates TGF-beta expression and suppresses kidney fibrosis induced by unilateral ureteral obstruction. J. Am. Soc. Nephrol. *25*, 2835–2846. https://doi.org/10.1681/asn.2013101068.

114. Koesters, R., Kaissling, B., Lehir, M., Picard, N., Theilig, F., Gebhardt, R., Glick, A.B., Hahnel, B., Hosser, H., Grone, H.J., et al. (2010). Tubular overexpression of transforming growth factor-β1 induces autophagy and fibrosis but not mesenchymal transition of renal epithelial cells. Am. J. Pathol. *177*, 632–643. https://doi.org/10.2353/ajpath.2010.091012.

115. Shen, J., Xu, S., Zhou, H., Liu, H., Jiang, W., Hao, J., and Hu, Z. (2017). IL-1β induces apoptosis and autophagy via mitochondria pathway in human degenerative nucleus pulposus cells. Sci. Rep. *7*, 41067. https://doi.org/10.1038/srep41067.

116. Lin, T.A., Wu, V.C.C., and Wang, C.Y. (2019). Autophagy in chronic kidney diseases. Cells *8*, 61. https://doi.org/10.3390/cells8010061.

117. Kaushal, G.P., Chandrashekar, K., Juncos, L.A., and Shah, S.V. (2020). Autophagy function and regulation in kidney disease. Biomolecules *10*, 100. https://doi.org/10.3390/biom10010100.

118. Menck, K., Sonmezer, C., Worst, T.S., Schulz, M., Dihazi, G.H., Streit, F., Erdmann, G., Kling, S., Boutros, M., Binder, C., et al. (2017). Neutral sphingomyelinases control extracellular vesicles budding from the plasma membrane. J. Extracell. Vesicles *6*, 1378056. https://doi.org/10.1080/20013078.2017.1378056.

119. Kalluri, R., and LeBleu, V.S. (2020). The biology, function, and biomedical applications of exosomes. Science *367*, eaau6977. https://doi.org/10.1126/science.aau6977.

120. Livshits, M.A., Khomyakova, E., Evtushenko, E.G., Lazarev, V.N., Kulemin, N.A., Semina, S.E., Generozov, E.V., and Govorun, V.M. (2015). Isolation of exosomes by differential centrifugation: theoretical analysis of a commonly used protocol. Sci. Rep. *5*, 17319. https://doi.org/10.1038/srep17319.

121. Ovesny, M., Krizek, P., Borkovec, J., Svindrych, Z., and Hagen, G.M. (2014). ThunderSTORM: a comprehensive ImageJ plug-in for PALM and STORM data analysis and super-resolution imaging. Bioinformatics *30*, 2389–2390. https://doi.org/10.1093/bioinformatics/btu202.

122. Livak, K.J., and Schmittgen, T.D. (2001). Analysis of relative gene expression data using real-time quantitative PCR and the $2-\Delta\Delta CT$ method. Methods *25*, 402–408. https://doi.org/10.1006/meth.2001.1262.

123. Contributors, W. (2021). FASTQ format. https://en.wikipedia.org/w/index.php?title=FASTQ_format&oldid=1039439009.

124. Frankish, A., Diekhans, M., Ferreira, A.M., Johnson, R., Jungreis, I., Loveland, J., Mudge, J.M., Sisu, C., Wright, J., Armstrong, J., et al. (2019). GENCODE reference annotation for the human and mouse genomes. Nucleic Acids Res. *47*, D766–D773. https://doi.org/10.1093/nar/gky955.

125. Blighe, K.R.S., and Lewis, M. (2021). EnhancedVolcano: Publication-Ready Volcano Plots with Enhanced Colouring and Labeling (Github). R package version 1120. https://github.com/kevinblighe/EnhancedVolcano.

126. Friedlander, M.R., Mackowiak, S.D., Li, N., Chen, W., and Rajewsky, N. (2012). miRDeep2 accurately identifies known and hundreds of novel microRNA genes in seven animal clades. Nucleic Acids Res. *40*, 37–52. https://doi.org/10.1093/nar/gkr688.

127. Paraskevopoulou, M.D., Georgakilas, G., Kostoulas, N., Vlachos, I.S., Vergoulis, T., Reczko, M., Filippidis, C., Dalamagas, T., and Hatzigeorgiou, A.G. (2013). DIANA-microT web server v5.0: service integration into miRNA functional analysis workflows. Nucleic Acids Res. *41*, W169–W173. https://doi.org/10.1093/nar/gkt393.

OMTN, Volume *28*

Supplemental information

Exosomal mitochondrial tRNAs and miRNAs as potential predictors of inflammation in renal proximal tubular epithelial cells

Glory Ranches, Maximilian Zeidler, Roman Kessler, Martina Hoelzl, Michael W. Hess, Jonathan Vosper, Paul Perco, Herbert Schramek, Kai K. Kummer, Michaela Kress, Anne Krogsdam, Michael Rudnicki, Gert Mayer, and Alexander Huettenhofer

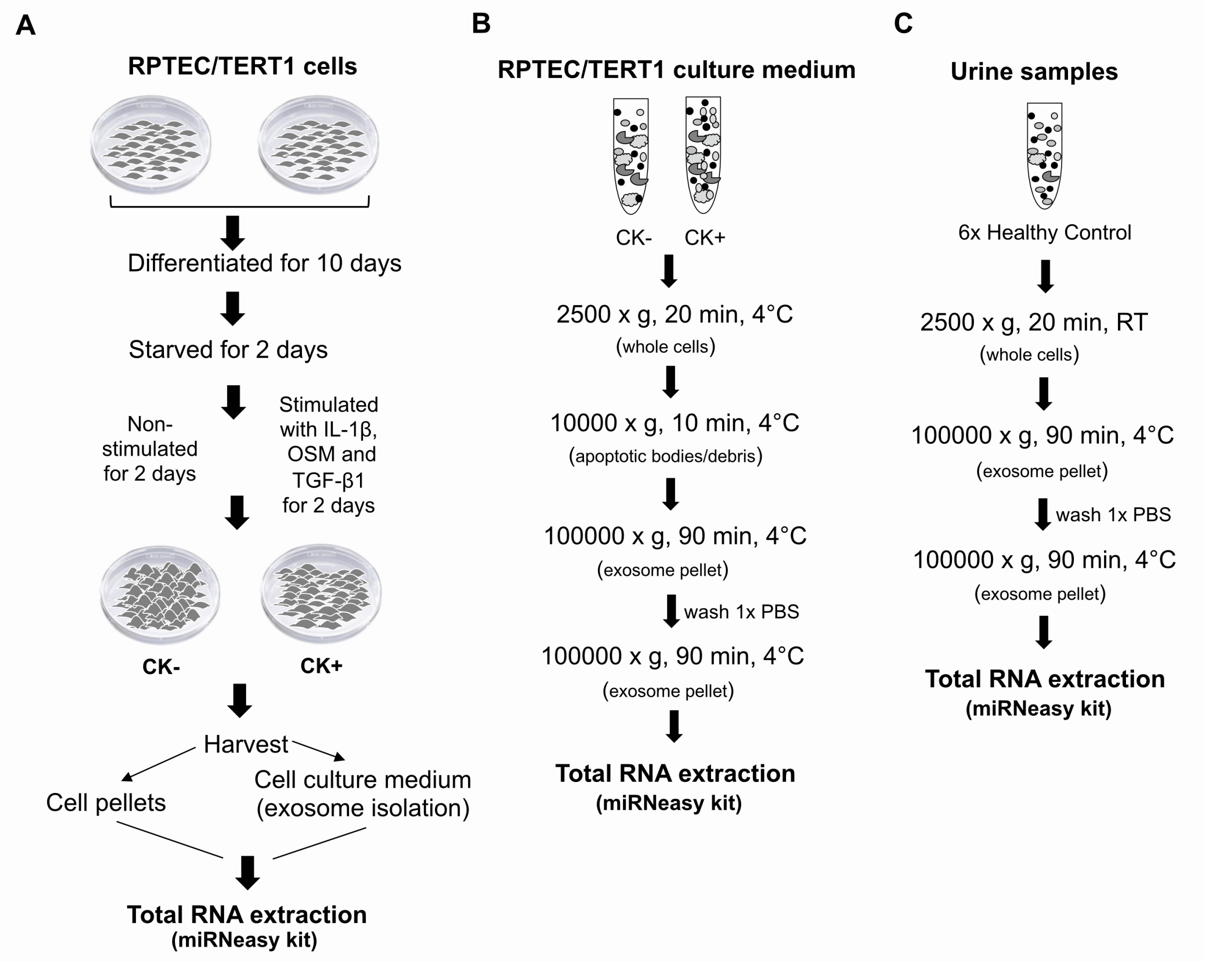

Figure S1. Schematic representation of RPTEC cytokine stimulation and exosome isolation methods employed in RPTEC culture medium and urine samples. A) An equal number of RPTECs were grown as describe in 'Materials and Methods'. CK- indicates non-stimulated cells and CK+ represents cytokine-stimulated cells or diseased-cells. **B)** An equal volume of RPTEC culture medium from each sample (A) were processed for ultracentrifugation as indicated and the resulting exosome pellets were analysed for total RNA extraction. **C)** An equal volume of urine samples from healthy control group (n=6) were processed for ultracentrifugation as indicated and the urinary exosome pellets were analysed similar to (B).

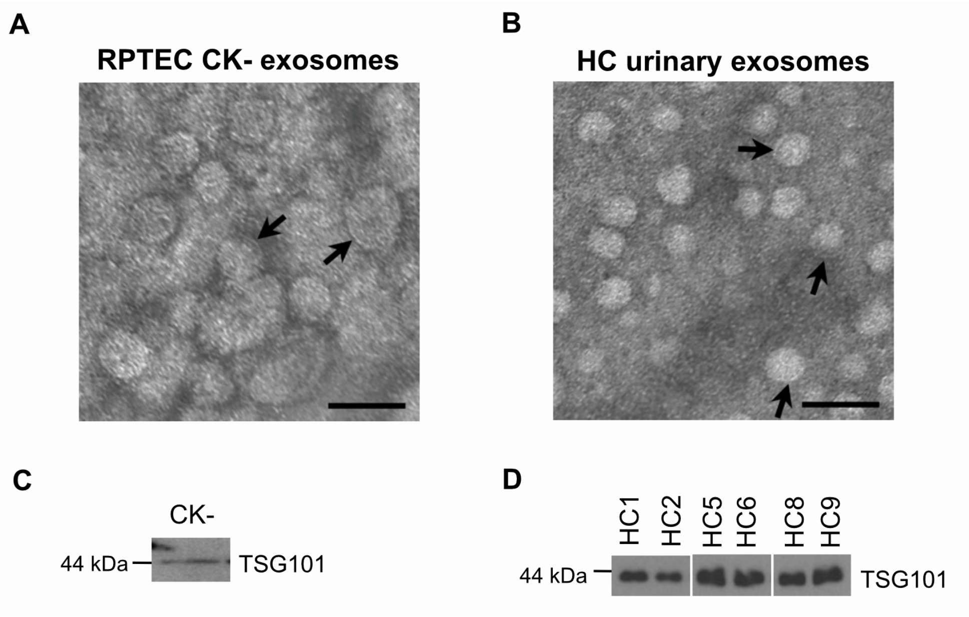

Figure S2. Characterisation of exosomes derived from non-stimulated RPTECs and healthy control urine samples. A) Transmission electron microscopy (TEM) of exosomes derived from non-stimulated RPTECs (CK-) and **B)** urinary exosomes from healthy control (HC) group; arrows point to the exosome membrane bilayer; scale bar= 50nm. **C)** Immunoblot analysis of TSG101 in exosomal lysates from CK- and **D)** urinary exosome samples obtained from HC group (n=6).

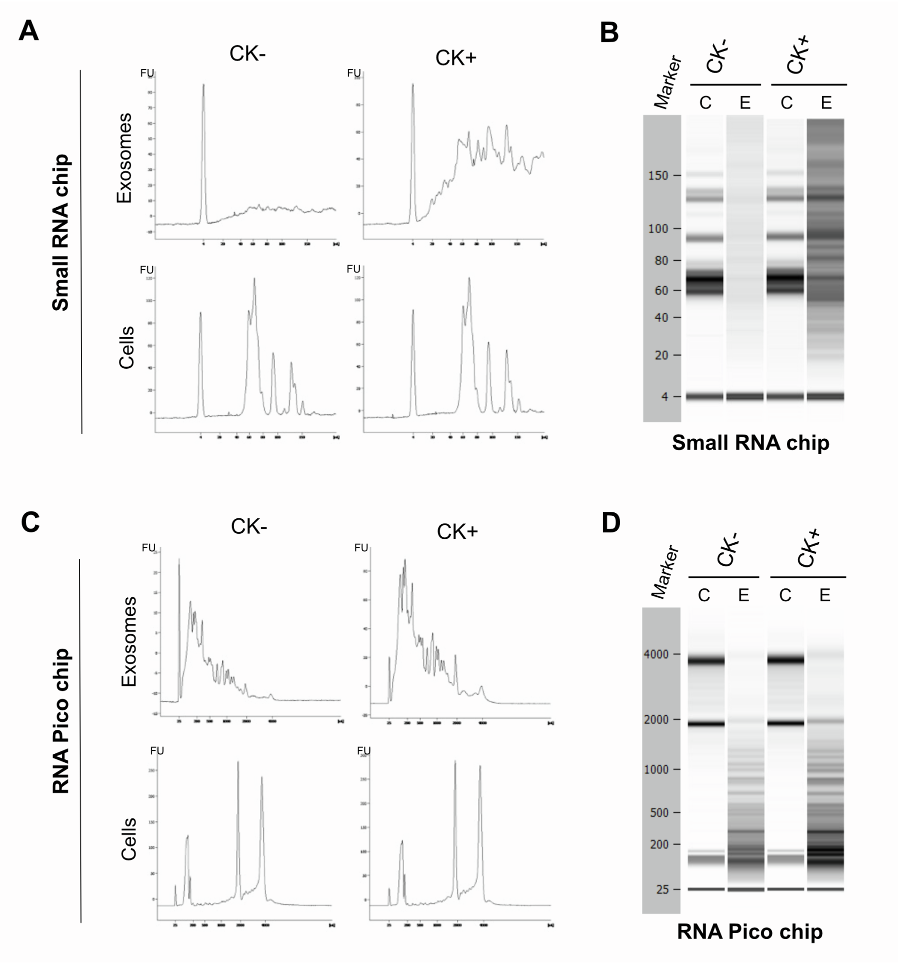

Figure S3. Bioanalyzer profile of total RNA extract from RPTEC exosomes or cells. A) Electropherogram profile of total exosomal RNA extracts (upper panels) and total cellular RNA extracts (lower panels) from either non-stimulated (CK-) or cytokine-stimulated cells (CK+) samples using small RNA chip (<200 nt). An equal amount of total cellular RNA (5 ng) and an equal volume of total exosomal RNA extract (1 µl) for each sample was used for loading. Total exosomal RNA extracts of CK- were undiluted (1:0) while CK+ was diluted in water (1:6). **B)** PAGE gel profile indicating the distribution and molecular size of total exosomal (E) and cellular (C) RNA populations of (A). **C)** Electropherogram profile of total exosomal RNA extracts (upper panels) and total cellular RNA extracts (lower panels) as in (A) using RNA pico chip. **D)** PAGE gel analysis showing the distribution and molecular size of exosomal (E) and cellular (C) RNA populations in (C).

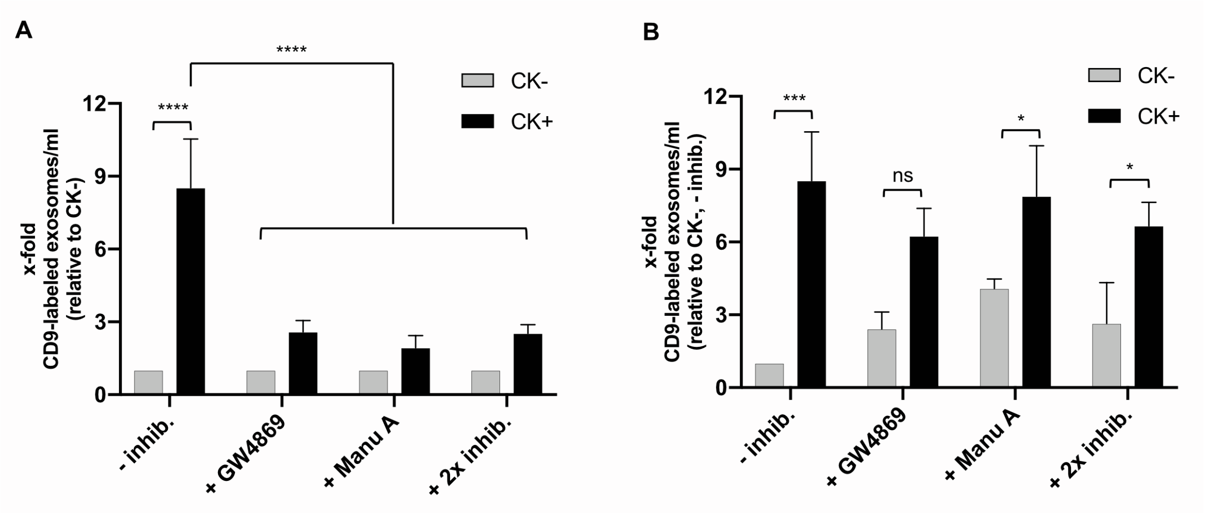

Figure S4. The effect of GW4869 and/or manumycin A on exosome release in RPTECs. A) The fold change (x-fold) of exosome release in cytokine-stimulated sample (CK+) relative to non-stimulated (CK-) sample in the absence or presence of a single inhibitor, (i.e. GW4869 or manumycin A (Manu A)) or two inhibitors (2x inhib.) was analysed by fluorescence-based detection of anti-CD9 (Alexa-647 nm)-labeled exosomes using microplate reader (see Materials and Methods). Bar graph represents mean ± SD of three independent experiments. Data were analysed using two-way ANOVA, with multiple comparisons and Bonferroni correction (GraphPad Prism, 8.0.1). **** indicates p<0.0001. **B)** Analysis of anti-CD9 (Alexa-647 nm)-labeled exosomes derived from CK- or CK+ RPTECs under similar conditions as in (A). The fold change (x-fold) of CD9-positive exosomes for each sample was calculated relative to CK- sample without inhibitor (-inhib.). Data were analysed using two-way ANOVA, with multiple comparisons and Bonferroni correction (GraphPad Prism, 8.0.1). * indicates p<0.05, *** indicates p<0.001 and ns represents not significant.

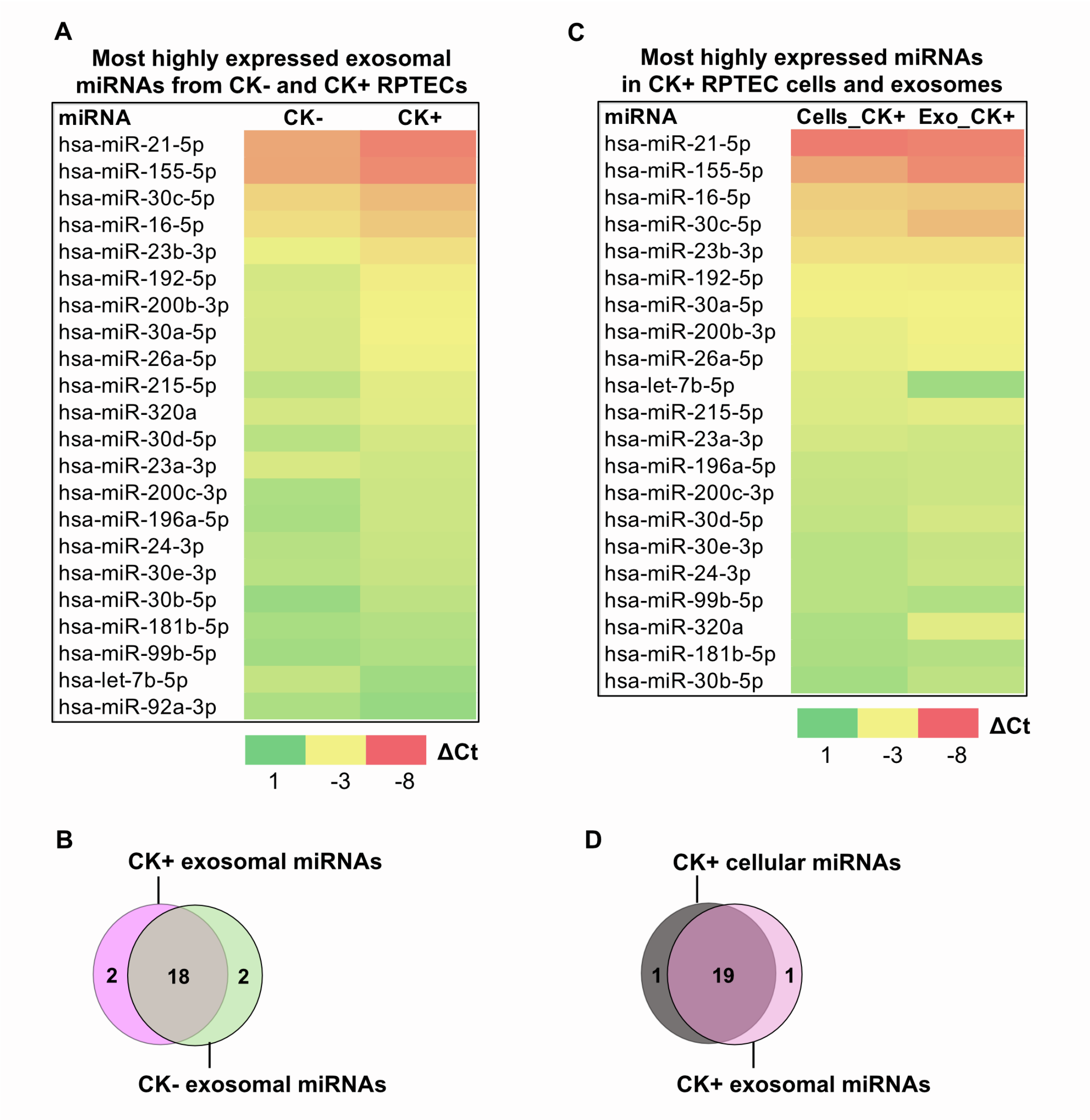

Figure S5. Most abundant exosomal miRNAs in RPTEC cells and exosomes. A) Heat map of 20 most highly abundant RPTEC exosomal miRNAs derived from non-stimulated (CK-) and cytokine-stimulated (CK+). Heat map colors indicate the normalized ΔCt ($Ct_{target\ miRNA}$ -$Ct_{reference\ 6x\ miRNAs}$) values of each target miRNA. Red indicates lower ΔCt (higher expression) and green indicates higher ΔCt (lower expression). **B)** Venn diagram of (A) showing the overlap of most highly abundant exosomal miRNAs between CK- and CK+. **C)** Most highly abundant miRNAs derived from CK+ RPTEC cells and exosomes. Similar analysis was performed as in (A). **D)** Overlap of (C) showing that the majority of most highly abundant miRNAs from CK+ RPTECs are secreted and released via exosomes.

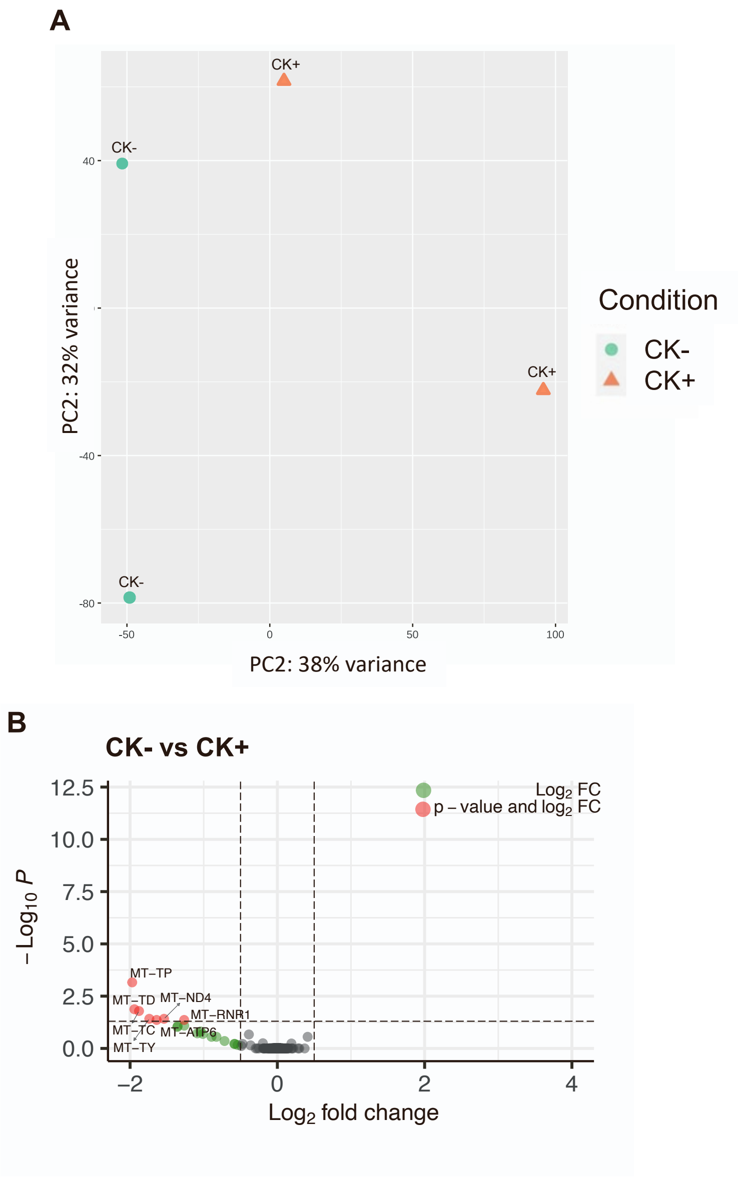

Figure S6. Analysis of differentially abundant exosomal RNAs. A) Principal component analysis (PCA) of exosomal RNA expression from cytokine-stimulated (CK+) and non-stimulated (CK-) RPTECs. **B)** Volcano plot analysis of differentially expressed exosomal mt-RNAs (adjusted p<0.05) derived from CK+ (red) and that from CK- RPTECs (green).

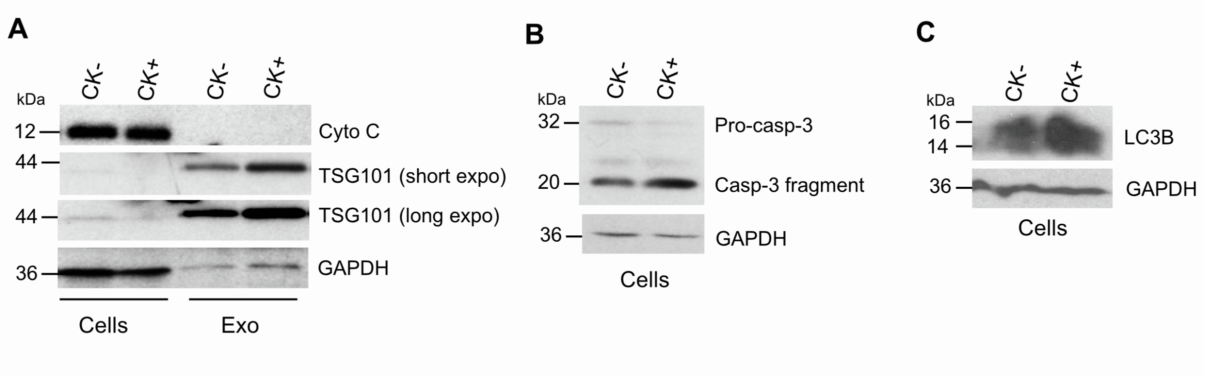

Figure S7. Immunoblot analysis of TSG10, cytochrome C, caspase-3 and LC3B.

RPTECs were either non-stimulated (CK-) or cytokine-stimulated (CK+). Total cell lysates (Cells) and exosomal lysates (Exo) under CK- or CK+ condition were analysed by SDS-PAGE and the protein expressions of **A)** exosome marker tumor suppressor gene (TSG101) and mitochondrial protein cytochrome C (Cyto C); **B)** pro-caspase-3 full length (Pro-casp-3) and caspase-3 fragment (Casp-3); and **C)** LC3B were detected by immunoblotting. GAPDH was used as a loading control. Western blot images (**A-C**) are representative of at least two independent experiments.

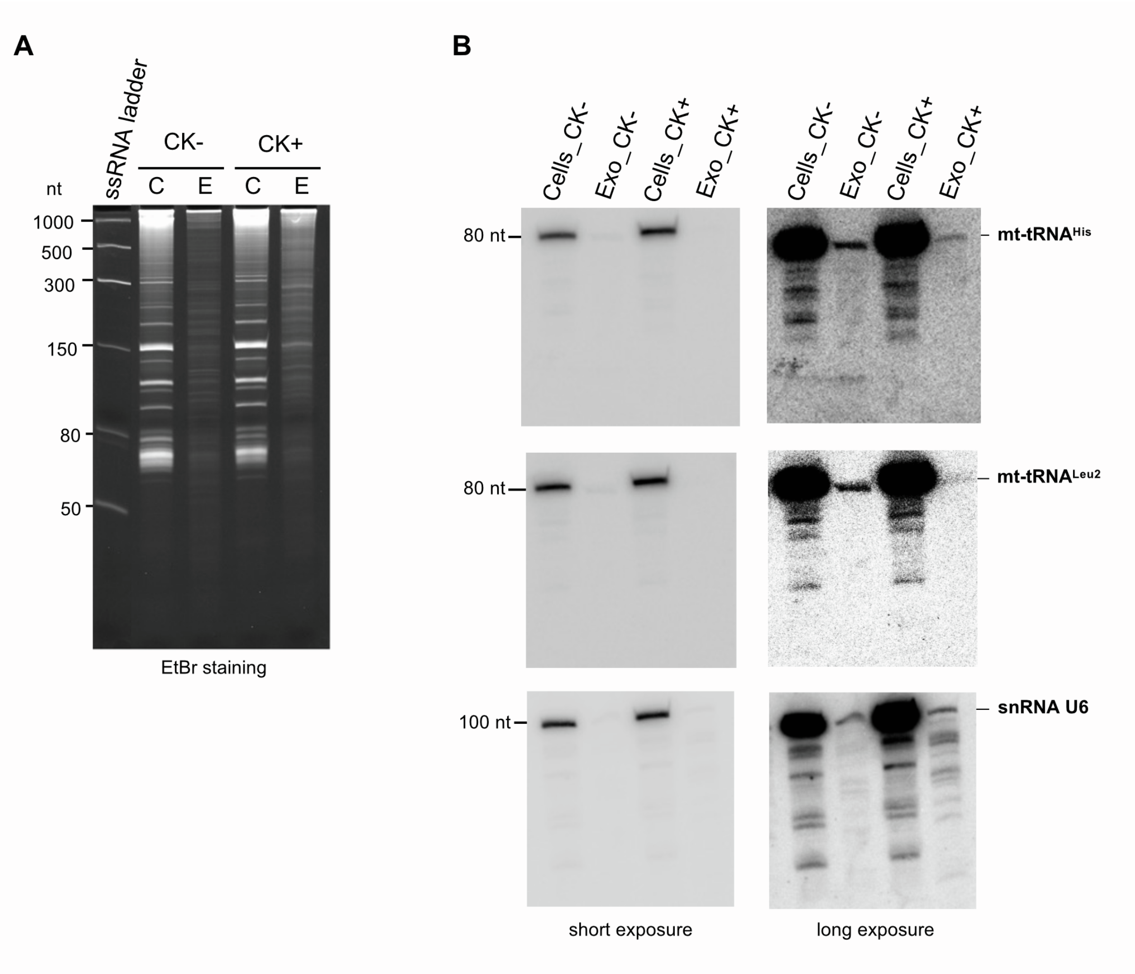

Figure S8. A) Ethidium bromide (EtBr) staining of total cellular RNA (C) and total exosomal RNA (E) derived either from non-stimulated (CK-) or cytokine-stimulated (CK+) RPTECs (see Materials and Methods). **B)** Northern blot analysis of mt-tRNA^{His}, mt-tRNA^{Leu2} and snRNA U6 (uncut membranes).

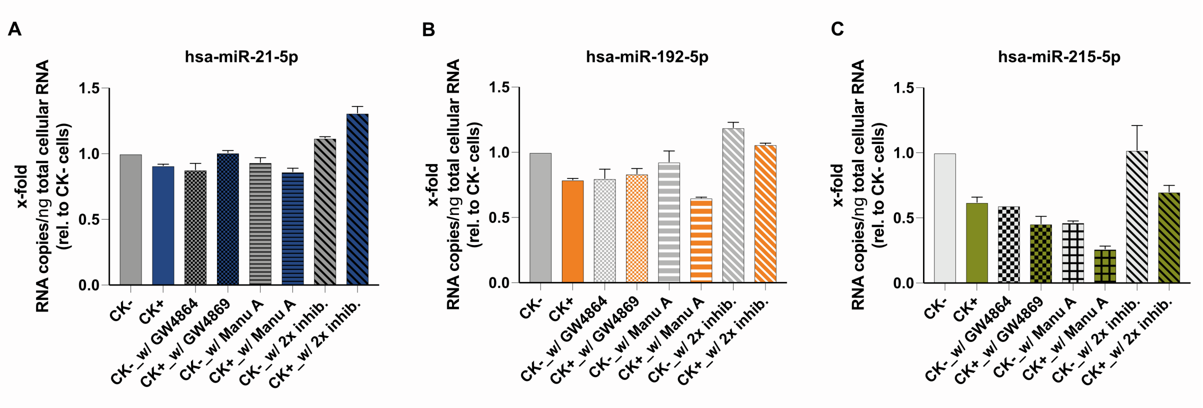

Figure S9. Comparison of cellular miRNAs, hsa-miR-21-5p, -215-5p and -192-5p expressions in RPTECs under different conditions. A-C) The expression of three candidate exosomal miRNAs under non-stimulated (CK-) or cytokine-stimulated (CK+), in the presence or absence of GW4869 and/or manumycin A (Manu A) (2x inhib.), was analysed by absolute quantification PCR (see Materials and Methods). The relative abundance (x-fold) of each candidate exosomal miRNA was determined based on the total RNA copy numbers/ng of total cellular RNA in each sample relative to that in CK- sample without inhibitor. Data represent mean ± SD of a single analysis (pool of three samples) in three replicates.

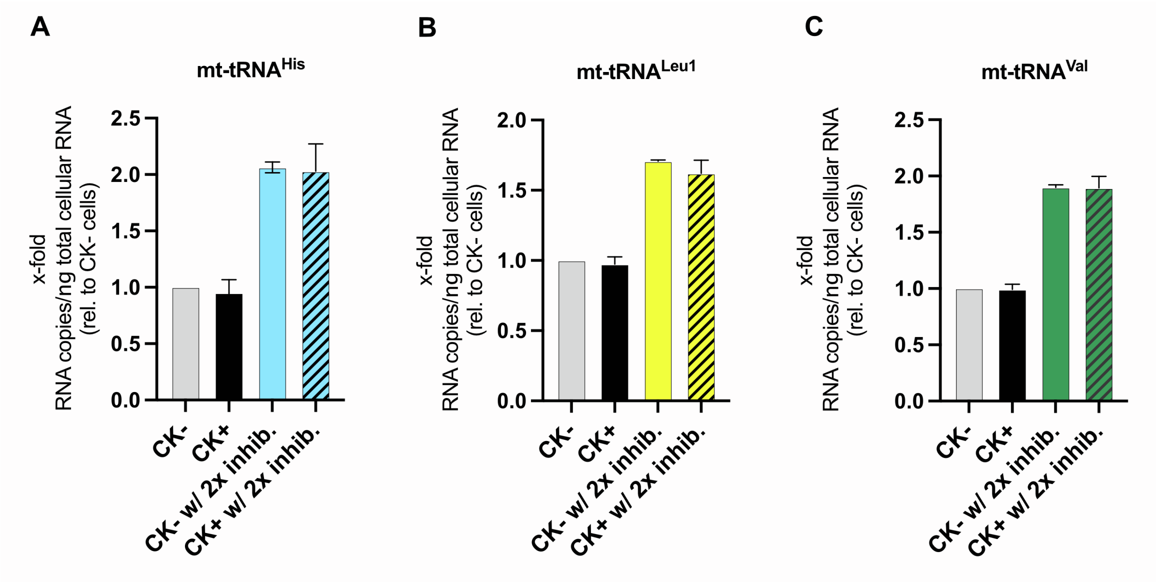

Figure S10. Comparison of cellular mt-tRNA (mt-tRNA^{His}, mt-tRNA^{Leu1} and mt-tRNA^{Val}) expressions in RPTECs under different conditions. A-C) The expression of three candidate exosomal mt-tRNAs under non-stimulated (CK-) or cytokine-stimulated (CK+), in the presence or absence of GW4869 and manumycin A (Manu A) (2x inhib.), was analysed by absolute quantification PCR (see Materials and Methods). The relative abundance (x-fold) of each candidate exosomal miRNA was determined based on the total RNA copy numbers/ng of total cellular RNA in each sample relative to that in CK- sample without inhibitor. Data represent mean ± SEM of two independent experiments.

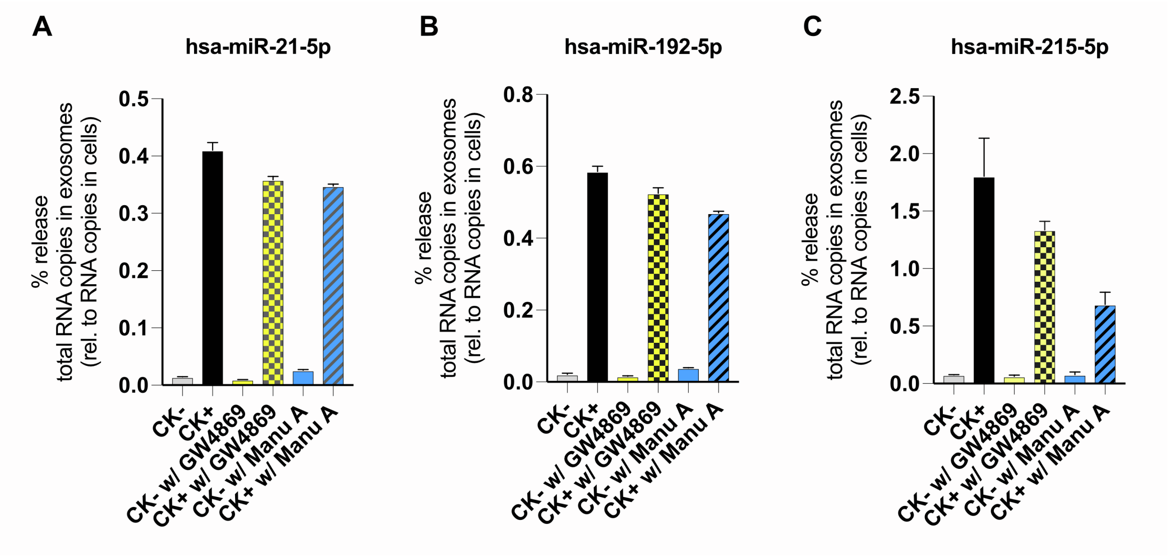

Figure S11. Effect of GW4869 and manumycin A on the cytokine-induced release of exosomal miRNAs, hsa-miR-21-5p, -215-5p and -192-5p from RPTECs. A-C-) RT-qPCR analysis of dysregulated exosomal miRNAs, hsa-miR-21-5p, -215-5p and -192-5p, derived from non-stimulated (CK-) or cytokine-stimulated (CK+) RPTECs treated with individual exosome inhibitor, GW4869 (5 μM) or manumycin A (Manu A) (250 nM). The RNA copy numbers of exosomal miRNAs were analysed by absolute quantification PCR using a synthetic miRNA (i.e. hsa-miR-21-5p, -215-5p or -192-5p) as a standard curve. The percentage (%) release of each dysregulated exosomal miRNA was calculated from the total cDNA copy number in exosomes over the total cDNA copy number in cells. Data represent mean ± SD of a single analysis, which consists of three independent samples pooled together.

Table S1. List of reference genes for normalization. Data represent mean and standard deviation (SD) of Ct values.

miRNA ID	Exosome samples		Cell samples		Urine samples	
	Mean	SD	Mean	SD	Mean	SD
hsa-let-7a-5p	27.22	2.34	32.09	0.47	26.78	1.57
hsa-let-7b-5p	26.46	1.93	29.86	0.69	27.36	1.66
hsa-miR-103a-3p	31.61	3.48	36.97	0.03	28.20	1.61
hsa-miR-191-5p	27.16	3.13	33.12	0.98	29.88	1.60
hsa-miR-26a-5p	24.95	3.21	29.64	0.51	27.49	1.88
hsa-miR-92a-3p	26.94	2.15	31.59	0.46	28.63	1.42

Table S2. Differentially abundant exosomal miRNAs in cytokine-stimulated (CK+) (n=3) and non-stimulated (CK-) (n=3) RPTECs.

miRNA ID	Fold change ($FC=2^{-\Delta\Delta Ct}$) CK+ / CK-	p-value
hsa-miR-31-3p	4.6	0.0318
hsa-miR-218-5p	3.4	0.0384
hsa-miR-146a-5p	3.1	0.0162
hsa-miR-130a-3p	3.1	0.0338
hsa-miR-421	2.9	0.0086
hsa-miR-21-5p	2.5	0.0011
hsa-miR-146b-5p	2.5	0.0088
hsa-miR-625-3p	2.5	0.0314
hsa-miR-126-5p	2.4	0.0393
hsa-miR-192-5p	2.3	0.0204
hsa-miR-215-5p	2.2	0.0227
hsa-miR-30b-5p	2.2	0.0327
hsa-miR-196a-5p	2.1	0.0405
hsa-miR-155-5p	2.1	0.0085
hsa-miR-30a-5p	2.0	0.0485
hsa-miR-151a-3p	2.0	0.0085
hsa-miR-200b-3p	1.9	0.0055
hsa-miR-200c-3p	1.9	0.0108
hsa-miR-361-5p	1.9	0.0237
hsa-miR-126-3p	1.9	0.0013
hsa-miR-30c-5p	1.8	0.0135
hsa-miR-30d-5p	1.8	0.0374
hsa-miR-182-5p	1.7	0.0149
hsa-miR-23b-3p	1.7	0.0011
hsa-miR-151a-5p	1.7	0.0474
hsa-miR-194-5p	1.6	0.0308
hsa-let-7a-5p	0.6	0.0346
hsa-miR-10b-5p	0.6	0.0392
hsa-let-7c-5p	0.4	0.0227
hsa-miR-663a	0.2	0.0071

Table S3. Analysis of most highly expressed exosomal RNAs from non-stimulated (CK-) and cytokine-stimulated (CK+) RPTECs by RNA-sequencing. Highlighted ncRNAs indicate the overlapping ncRNAs between CK- and CK+.

CK-			CK+		
**Gene ID**	**Gene_biotype**	**Average of normalized counts**	**Gene ID**	**Gene_biotype**	**Average of normalized counts**
**MT-RNR2**	Mt_rRNA	14699	**AC073140.2**	lncRNA	14346
**AC073140.2**	lncRNA	10499	**MT-RNR2**	Mt_rRNA	7350
**MT-TM**	Mt_tRNA	7740	**RNY1**	misc_RNA	6458
**MT-RNR1**	Mt_rRNA	6385	**SNORD100**	snoRNA	4673
**SNORD100**	snoRNA	5058	**MIR30A**	miRNA	4306
**RNY1**	misc_RNA	4637	**MT-RNR1**	Mt_rRNA	2160
MT-TS2	Mt_tRNA	3125	**MIR10B**	miRNA	1784
**MIR30A**	miRNA	3016	**MIR31**	miRNA	1723
MT-TL2	Mt_tRNA	2030	**VTRNA1-1**	misc_RNA	1464
MT-TH	Mt_tRNA	1864	**GAS5**	lncRNA	1412
**VTRNA1-1**	misc_RNA	1741	**MT-TM**	Mt_tRNA	1401
**MIR10B**	miRNA	1626	MIR200A	miRNA	1210
MT-TV	Mt_tRNA	1317	**SNORD6**	snoRNA	1138
**MIR31**	miRNA	1217	**SNORD69**	snoRNA	1074
**SNORD69**	snoRNA	1129	MIR200B	miRNA	951
**GAS5**	lncRNA	1040	MIR221	miRNA	926
**SNORD6**	snoRNA	1001	MIR30D	miRNA	888
RNY4	misc_RNA	960	MIRLET7G	miRNA	853
MIR3591	miRNA	925	SNORD99	snoRNA	762
RNY3	misc_RNA	848	MIR29A	miRNA	746

Table S4. Differentially abundant exosomal mtRNAs from cytokine-stimulated relative to non-stimulated RPTECs.

Gene ID	Log2 FC	p-value	adjusted p-value
MT-TV	-2.82	1.657E-11	6.349E-08
MT-TI	-2.57	7.755E-07	3.301E-04
MT-TS2	-2.40	1.066E-09	1.361E-06
MT-TR	-2.34	4.031E-07	2.574E-04
MT-TL2	-2.25	3.581E-10	6.859E-07
MT-TH	-2.14	1.644E-08	1.575E-05
MT-TM	-2.09	2.244E-07	1.719E-04
MT-TL1	-2.09	5.379E-07	2.944E-04
MT-TF	-2.02	6.438E-07	3.083E-04
MT-TP	-1.97	1.804E-06	6.912E-04
MT-TD	-1.94	3.839E-05	1.337E-02
MT-TC	-1.88	5.093E-05	1.626E-02
MT-TY	-1.74	1.329E-04	3.831E-02
MT-ATP6	-1.64	1.808E-04	4.347E-02
MT-ND4	-1.54	1.400E-04	3.831E-02
MT-RNR1	-1.27	1.816E-04	4.347E-02

Table S5. Overlap of dysregulated exosomal miRNAs in the CKD group (Next generation Sequence Analysis of clinical samples)[19] and RPTEC CK+ group (diseased cells) (RT-qPCR analysis using LNA miRNA miRNome Human Panel I).

CKD stage	miRNA ID	Clinical samples (CKD vs. Healthy control)		RPTECs (CK+ vs. CK-)	
		Log2 Fold Change	p-value adjusted	Fold change	p-value
I	hsa-miR-215-5p	3.7	0.0122	2.2	0.0227
II	hsa-miR-126-5p	3.7	0.0023	2.4	0.0393
	hsa-miR-126-3p	2.2	0.0458	1.9	0.0013
	hsa-miR-21-5p	1.5	0.0217	2.5	0.0011
	hsa-miR-215-5p	1.3	0.0931	2.2	0.0227
	hsa-miR-192-5p	1.2	0.0531	2.3	0.0204
	hsa-miR-31-3p	1.2	0.0789	4.6	0.0318
	hsa-miR-23b-3p	0.6	0.0789	1.7	0.0011
III	hsa-miR-215-5p	3.8	0.0146	2.2	0.0227
	hsa-miR-146a-5p	2.3	0.0110	3.1	0.0162
IV	hsa-miR-215-5p	3.5	0.0270	2.2	0.0227
	hsa-miR-192-5p	2.7	0.0349	2.3	0.0204
	hsa-miR-146a-5p	1.5	0.0864	3.1	0.0162
	hsa-miR-23b-3p	0.6	0.0991	1.7	0.0011

Table S6. Overlap of dysregulated exosomal mt-tRNAs in the CKD group[19] and RPTEC CK+ group (diseased cells).

CKD stage	mt-tRNA ID	Clinical samples (CKD vs. Healthy control)		RPTECs (CK+ vs. CK-)	
		Log2 Fold Change	p-value adjusted	Log2 Fold Change	p-value adjusted
I	MT-TC	-3.1	9.41E-05	-1.88	1.63E-02
	MT-TI	-2.9	3.84E-03	-2.57	3.30E-04
	MT-TL2	-2.9	7.41E-04	-2.25	6.86E-07
	MT-TS2	-2.7	3.84E-03	-2.40	1.36E-06
	MT-TL1	-2.6	6.40E-04	-2.09	2.94E-04
	MT-TH	-2.5	6.14E-04	-2.14	1.57E-05
	MT-TM	-2.5	7.02E-04	-2.09	1.72E-04
	MT-TV	-2.5	1.12E-03	-2.82	6.35E-08
	MT-TP	-2.4	7.33E-03	-1.97	6.91E-04
	MT-TY	-2.2	2.00E-02	-1.74	3.83E-02
	MT-TR	-2.0	2.90E-02	-2.34	2.57E-04
	MT-TF	-1.9	7.42E-03	-2.02	3.08E-04
	MT-TD	-1.5	7.78E-02	-1.94	1.34E-02
II	MT-TD	-1.9	4.96E-02	-1.94	1.34E-02
	MT-TR	-1.8	7.85E-02	-2.34	2.57E-04
	MT-TC	-1.7	2.97E-02	-1.88	1.63E-02
	MT-TP	-1.6	8.98E-02	-1.97	6.91E-04
	MT-TL2	-1.4	9.94E-02	-2.25	6.86E-07
	MT-TH	-1.2	8.46E-02	-2.14	1.57E-05
III	MT-TD	-3.2	2.91E-03	-1.94	1.34E-02
	MT-TL1	-2.6	1.85E-03	-2.09	2.94E-04
	MT-TL2	-2.4	6.56E-03	-2.25	6.86E-07
	MT-TC	-1.9	1.30E-02	-1.88	1.63E-02
	MT-TM	-1.9	9.93E-03	-2.09	1.72E-04
	MT-TP	-1.8	5.83E-02	-1.97	6.91E-04
	MT-TI	-1.8	6.88E-02	-2.57	3.30E-04
	MT-TH	-1.8	1.56E-02	-2.14	1.57E-05
	MT-TF	-1.4	6.31E-02	-2.02	3.08E-04
	MT-TV	-1.3	8.98E-02	-2.82	6.35E-08
IV	MT-TC	-2.0	2.04E-03	-1.88	1.63E-02
	MT-TP	-1.6	7.16E-02	-1.97	6.91E-04
	MT-TL1	-1.6	4.15E-03	-2.09	2.94E-04
	MT-TY	-1.8	4.17E-02	-1.74	3.83E-02

Table S7. List of primers and template sequences.

#	Name	Sequences (5' to 3')	Assay	Source
1	hsa-miR-21-5p	UAGCUUAUCAGACUGAUGUUGA	PCR	YP00204230 (Qiagen)
2	hsa-miR-192-5p	CUGACCUAUGAAUUGACAGCC	PCR	YP00204099 (Qiagen)
3	hsa-miR-215-5p	AUGACCUAUGAAUUGACAGAC	PCR	YP00204598 (Qiagen)
4	hsa-miR-194-5p	UGUAACAGCAACUCCAUGUGGA	PCR	YP00204080 (Qiagen)
5	hsa-miR-126-3p	UCGUACCGUGAGUAAUAAUGCG	PCR	YP00204227 (Qiagen)
6	hsa-miR-146a-5p	UGAGAACUGAAUUCCAUGGGUU	PCR	YP00204688 (Qiagen)
7	hsa-miR-21-5p	TAGCTTATCAGACTGATGTTGA	PCR	IDT
8	hsa-miR-192-5p	CTGACCTATGAATTGACAGCC	PCR	IDT
9	hsa-miR-215-5p	ATGACCTATGAATTGACAGAC	PCR	IDT
10	Universal primer rev	GAATCGAGCACCAGTTACGCA	PCR	IDT
11	hsa-miR-21a-5p template w/ tag	GAATCGAGCACCAGTTACGCATTTTGCTCAACATCAGTCTGATAAGCTA	PCR	IDT
12	hsa-miR-192-5p template w/ tag	GAATCGAGCACCAGTTACGCATTTTGCGGCTGTCAATTCATAGGTCAG	PCR	IDT
13	hsa-miR-215-5p template w/ tag	GAATCGAGCACCAGTTACGCATTTTGCGTCTGTCAATTCATAGGTCAT	PCR	IDT
14	SNORD100 fwd	TACATGATGACAACTGGCTCC	PCR	IDT
15	mt-tRNA^{His} fwd	GATTGTGAATCTGACAACAGAGG	PCR	IDT
16	mt-tRNA^{Leu2} fwd	CCATTGGTCTTAGGCCCCA	PCR	IDT
17	mt-tRNA^{Val} fwd	GCTTAACACAAAGCACCCAACT	PCR	IDT
18	mt-tRNA^{Phe} fwd	CCTCCTCAAAGCAATACACTGA	PCR	IDT
19	mt-tRNA^{Leu1} fwd	GAGCCCGGTAATCGCATAAAAC	PCR	IDT
20	mt-tRNA^{His} fwd_w/ T7 promoter sequence	AGGTAATACGACTCACTATAGGGTAAATATAGTTTAACC	PCR	IDT
21	mt-tRNA^{Leu1} fwd_w/ T7 promoter sequence	AGGTAATACGACTCACTATAGGGTTAAGATGGCAGAGCC	PCR	IDT
22	mt-tRNA^{Val} fwd_w/ T7 promoter sequence	AGGTAATACGACTCACTATAGGCAGAGTGTAGCTTAACAC	PCR	IDT
23	mt-tRNA^{His} rev	GGTAAATAAGGGGTCGTAAGC	PCR	IDT
24	mt-tRNA^{Leu1} rev	TGTTAAGAAGAGGAATTGAACC	PCR	IDT
25	mt-tRNA^{Val} rev	TCAGAGCGGTCAAGTTAAG	PCR	IDT
26	mt-tRNA^{His} template	GTAAATATAGTTTAACCAAAACATCAGATTGTGAATCTGACAACAGAGGCTTACGACCCCTTATTTACC	PCR	IDT
27	mt-tRNA^{Leu1} template	GTTAAGATGGCAGAGCCCGGTAATCGCATAAAACTTAAAACTTTACAGTCAGAGGTTCAATTCCTCTTCTTAACA	PCR	IDT
28	mt-tRNA^{Val} template	CAGAGTGTAGCTTAACACAAAGCACCCAACTTACACTTAGGAGATTTCAACTTAACTTGACCGCTCTGA	PCR	IDT
29	mt-tRNA^{His} rev	CCTCTGTTGTCAGATTCACAATC	northern blot	IDT
30	mt-tRNA^{Leu2} rev	TGGGGCCTAAGACCAATGG	northern blot	IDT
